# BODIPY-Doped Nanohoops In and Out of Conjugation

**DOI:** 10.1021/acs.orglett.5c01301

**Published:** 2025-05-05

**Authors:** Sebastian H. Röttger, Pia A. Mader, Heinrich F. von Köller, Peter G. Jones, Daniel B. Werz

**Affiliations:** † DFG Cluster of Excellence livMatS@FIT and Institute of Organic Chemistry, University of Freiburg, 79104 Freiburg, Germany; ‡ Institute of Organic Chemistry, University of Freiburg, 79104 Freiburg, Germany; § Institute of Organic Chemistry, Justus Liebig University Giessen, 35392 Giessen, Germany; ∥ Institute of Inorganic and Analytical Chemistry, Technical University of Braunschweig, 38106 Braunschweig, Germany

## Abstract

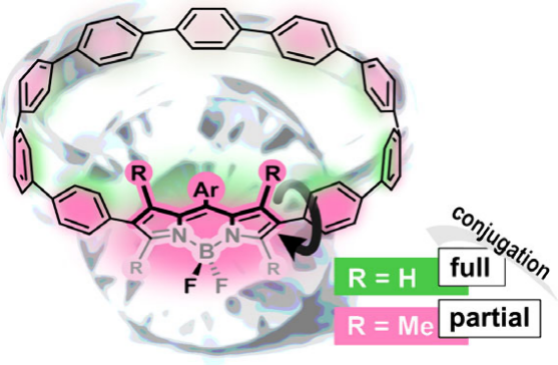

By combining the well-known motifs of BODIPY dyes and cycloparaphenylenes, novel nanohoop derivatives were accessible via established procedures. Absorption and emission spectra showed overall bathochromic shifts, and their photophysical behavior can be tuned by introducing steric demand to modulate the conjugation throughout the system. ^19^F NMR spectra underline distinct differences in the conformations, and (TD)DFT calculations provide a deeper insight into the geometry, photophysical behavior, and influence of steric demand.

Carbon nanohoops, first reported by Jasti and Bertozzi^1^ with a series of [*n*]cycloparaphenylenes (CPPs) (Figure 1A, top), have become a continuously growing branch of synthetic organic material science; they exhibit structural versatility and unique properties.^2^ Their synthesis is well-established^3^ and manipulation of their properties has been achieved via various methods, e.g. doping the ring with one *meta*-substituted benzene unit to amplify fluorescence^4^ and/or an alkyne linker (Figure 1A, middle), allowing postfunctionalization via click chemistry.^5^ Extensions to different dimerization modes have also been reported.^3d,6^ The incorporation of disruptive units, such as dibenzopentalene influences photophysical characteristics even further, leading to antiaromaticity.^7^ In 2020, an isoelectronic B–N-doped example^8^ and a benzothiadiazole derivative,^9^ resulting in a strong red-shift (Figure 1A, bottom), were first reported. BODIPYs and their derivatives have found constant use in the field of dyes because of their synthetic accessibility, versatile tunability and outstanding photophysical properties.^10^ As well as strong attenuation coefficients and high fluorescence quantum yields, bathochromic shifts are another key goal commonly achieved by extending the π-system, e.g. via oligomerization.^11^ Low energy excitations play an important role when it comes to medicinal applications such as imaging or photodynamic therapy to enable excitation at lower frequencies.^12^ The pyrrole periphery of the BODIPY motif has proven to be a versatile site for extending the π-system, e.g., through electrophilic substitution or cross-coupling reactions. (Figure 1B, top).^13^ Depending on the steric hindrance introduced at the BODIPY core (e.g., methyl groups), the photophysical properties of the conjugate can be tuned by adjusting the overall conjugation (Figure 1B).^14^

**Figure 1:**
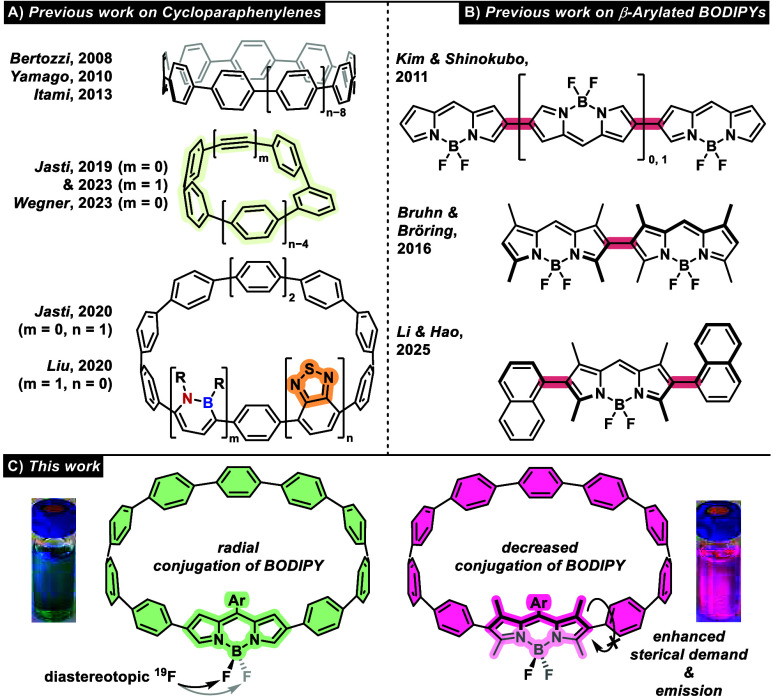
Previous work on (A) cycloparaphenylenes and (B) arylated BODIPYs (*meso* substituents have been omitted for clarity). (C) This work.

Herein we present two cyclic CPP-BODIPY conjugates with modulated electronic properties. Since similar systems have been recently reported with various ring sizes^15^ and different substitution patterns,^16^ we decided to focus on varying the BODIPY core and to study the influence of higher substitution to force the removal of the dye motif from the conjugated system. Incorporation of a 1,3,5,7-tetramethyl-substituted BODIPY core was contrasted with an unsubstituted variant displaying minimal steric hindrance.^13,14a^ The synthetic access was realized via literature procedures. First, a borylated 9-membered CPP building block (BB) **2**(8,17) and appropriate β,β-diiodinated BODIPYs **1a** and **1b** were converted via Suzuki–Miyaura coupling using Pd(OAc)_2_ as precatalyst, SPhos as ligand and K_3_PO_4_ as base in a solvent mixture of 1,4-dioxane and water (15:1) at 80 °C to provide cyclized intermediates **3** (confirmed by HRMS, see Figures S35 and S36). Subsequent reductive aromatization thereof with *in situ* generated H_2_SnCl_4_ in THF at ambient temperature (Scheme 1)^3f,7b^ gave BODIPY-doped nanohoops **4a** as a dark green solid in 13% yield and **4b** as a magenta solid in 8% yield, via a two-step one-pot procedure. The iodinated BODIPYs were obtained via a literature-known method.^18^ Corresponding bromides proved to be incompatible with the coupling protocol. Synthesis of the 9-membered CPP BB **2** was performed using literature methods.^8,17^

**Scheme 1:**
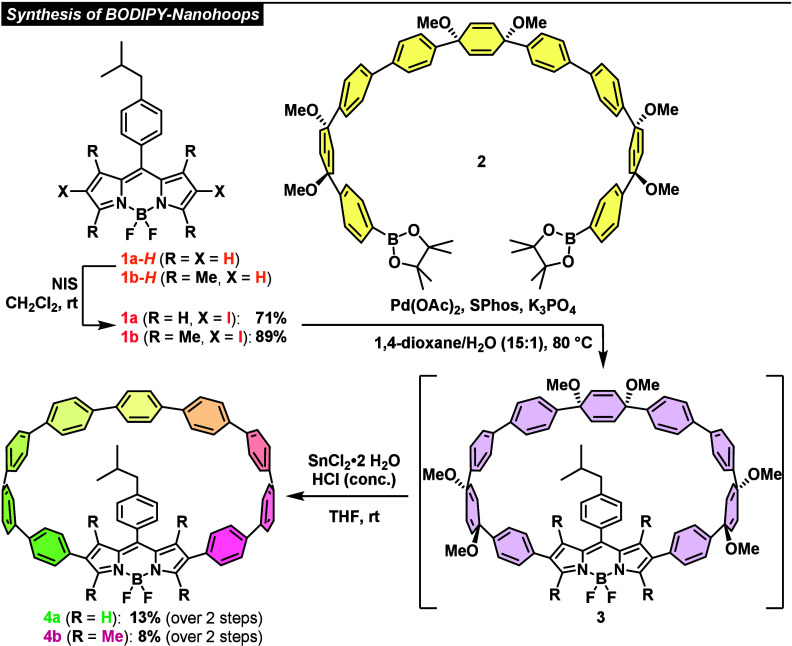
Synthetic Route toward BODIPY-Doped CPPs

The absorption profiles of BODIPY-CPP hybrids **4a**–**b** both consist of two major regions, which correspond to the sum of both chromophoric moieties (Figure 2). The main absorption regions of **4a** and **4b** (275–420 nm) can be assigned to the CPP part and align similarly for both, whereas the second band (519–754 nm for **4a** and 463–646 nm for **4b**) appears to be the typical S_1_ ← S_0_ transition of the BODIPY unit, bathochromically shifted, however, as result of the linkage to the CPP backbone. As anticipated, because of decreased π-orbital overlap with the curved π-system of the hoop, caused by the steric demand of the four methyl groups, the red-shift of **4b** (Δλ_max_^A^ = 32 nm) is not as marked as that of its unsubstituted congener **4a** (Δλ_max_^A^ = 96 nm). In addition, the absorption band of **4a** is broadened, resulting in an attenuation coefficient approximately half that of **4b** (Figure 2). We assume that the stronger conjugation with the CPP backbone enhances access to CPP-dominated vibrational modes, thereby enabling a diversified vibronic coupling. Furthermore, the bathochromic shift of **4a** compared to **4b** is significantly stronger than the negligible shift of **1a-*****H*** versus **1b-*****H***, highlighting the influence of the β-substitution on the π-conjugation to the backbone. **4a** shows a negligible emission at 710 nm in CH_2_Cl_2_, whereas **4b** displays a visible fluorescence (λ_max_^F^ = 613 nm), which is presumably the result of a more autonomous BODIPY core suppressing geometrical relaxation processes of the excited state along both the *meso* and the equatorial axis.^19^ This interpretation is further supported by the lower Stokes shift of **4b** compared to **4a**. Emission spectra show an anticipated decrease in intensity at increasing temperatures, both for **4a** and **4b** (Figure S48).

**Figure 2:**
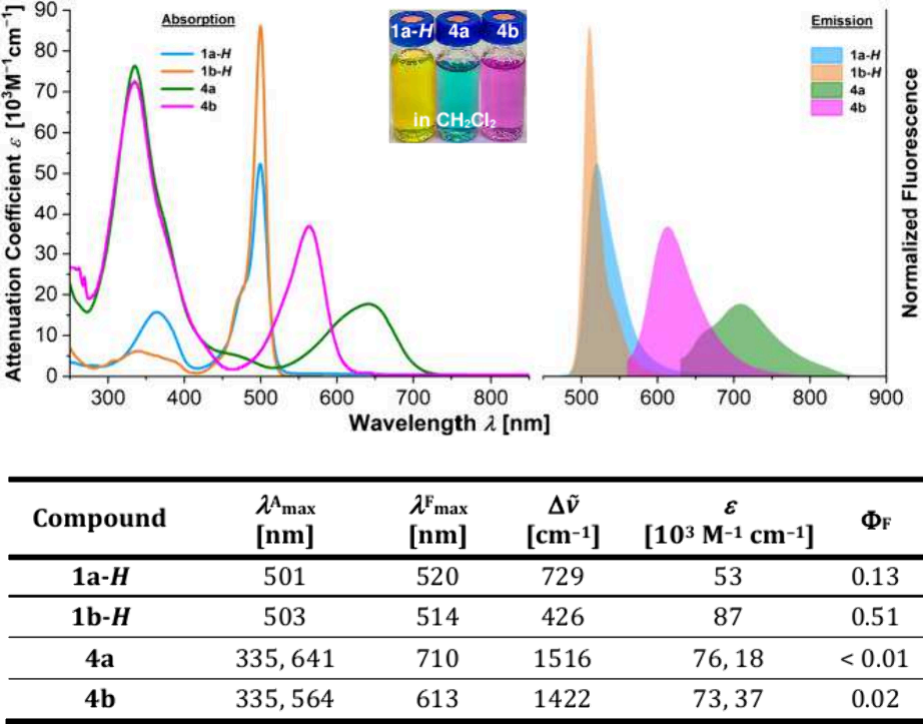
Absorption and emission spectra (top) and data (bottom) of BODIPY-doped nanohoops and their precursors. Absorption and emission spectra were recorded in solutions of CH_2_Cl_2_ at room temperature. Further spectroscopic data are given in Table S1.

Interestingly, the different conformations were also observable in the ^19^F NMR spectra (659 MHz). In CDCl_3_ at room temperature, **4a** consists of two clearly distinguishable signals at −142 and −163 ppm, corresponding to one of each fluorine nucleus showing not only the typical ^1^*J*_F–B_ coupling (∼24–30 Hz) but also ^2^*J*_F–B–F_ coupling (∼92–94 Hz) (see the Supporting Information). This signal splitting results from their different chemical environments concomitant with the loss of the mirror plane along the BODIPY scaffold. Similar observations have already been reported for *meso*-(*o*-aryl) substituents.^20^ In contrast, **4b** does not show this defined signal splitting at room temperature and instead exhibits a single broad signal (−141 to −148 ppm). However, decreasing the temperature led to a splitting into two broad signals (Figure S31). Thus, we investigated further temperatures to possibly find a coalescence point and also decided to use PhMe-*d*_8_ instead of CDCl_3_ to access higher temperatures. For **4b**, the ^19^F coalescence temperature is *T*_c_ = 280 K (Figure S32), whereas only slight changes of chemical shifts were observed and thus no ^19^F coalescence was found in PhMe-*d*_8_ for **4a** between 235 and 370 K (Figure S34), underlining the generally favored conformation in conjugation with the CPP moiety. According to a variation of the Eyring equation (eq S1),^21^ the free activation enthalpy of the ^19^F coalescence of **4b** was calculated to be Δ*G*_280_^⧧^ = 46.8 kJ/mol [Δν = 4.78 kHz (659 MHz measuring frequency)]. An excerpt of the temperature measurements, comparing both specimens around the coalescence temperature of **4b**, is shown in Figure 3.

**Figure 3:**
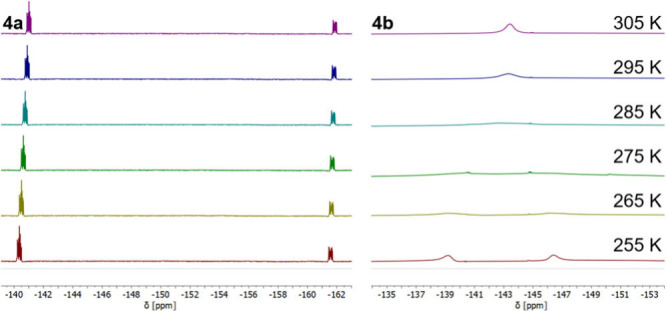
^19^F NMR (659 MHz, PhMe-*d*_8_) spectra of **4a** (left) and **4b** (right) from 255 to 305 K in steps of 10 K.

DFT calculations were performed on compounds **4a** and **4b** to elucidate their structural and electronic characteristics. Geometry optimizations revealed a substantial increase in the dihedral angle between the BODIPY core and the adjacent phenyl ring of the CPP backbone for **4a** (17°) compared to **4b** (44°), accompanied by a corresponding rise in strain energy from 51.6 kcal/mol (**4a**) to 55.3 kcal/mol (**4b**). Despite differing π-conjugational overlap between both species, frontier orbital analysis (Figure 4) reveals predominant localization at the BODIPY unit for both systems, however, with increased contributions from the CPP backbone. The increased conjugation in **4a** correlates with a decreased HOMO–LUMO gap (4.11 eV vs 4.46 eV). Lower-lying orbitals showed less BODIPY participation, being principally distributed across phenylene subunits.

**Figure 4:**
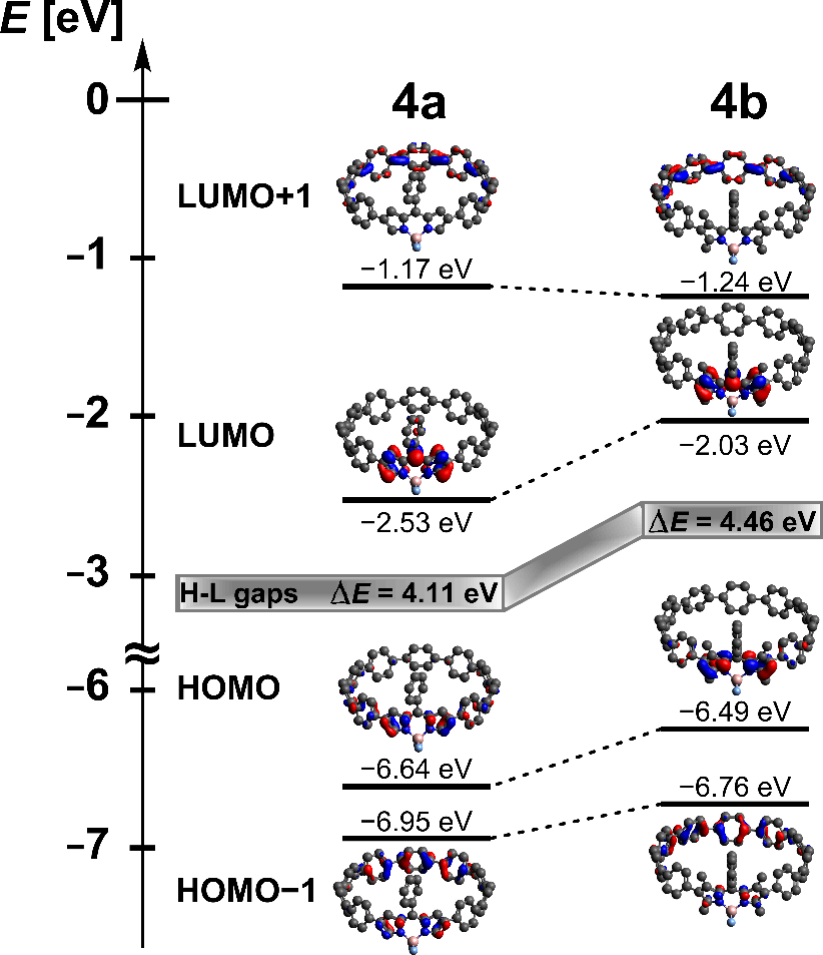
Calculated energies and pictorial presentation of frontier orbitals of BODIPY-doped nanohoops **4a** and **4b**. The *iso*-butyl groups of the *meso* substituents were replaced by hydrogens, all of which have been omitted for simplification. Geometry (DFT): M06-2X/def2-TZVP. All computations *in vacuo*.

TDDFT simulations assign the first excited states S_1_ (λ_max,exp_ = 641 nm for **4a**, 564 nm for **4b**) to a HOMO→LUMO excitation. Plotting the charge density difference (CDD) clearly reveals charge transfer (CT) characteristics for the S_2_ states of **4a** and **4b** (Figures S57 and S58). Both CT transitions indicate an electronically enriched BODIPY core at the cost of the CPP periphery.

In conclusion, we have successfully incorporated two distinct BODIPY units into a cyclic cycloparaphenylene backbone giving rise to two hybrid superstructures with differing photophysical properties. By using the β-position of BODIPYs for linkage, their steric demand was leveraged to construct either a fully conjugated cyclic BODIPY-CPP hybrid (**4a**) or a congener with heterogeneous coupling conditions and interrupted conjugation (**4b**). While the increased conjugation in **4a** resulted in a red shift extending to the edge of the visible spectrum (700 nm), the autonomous BODIPY unit in **4b** retained the fluorescence of the hybrid superstructure. Temperature-dependent ^19^F NMR analyses supported the optical spectroscopic findings indicating a rigidly inserted BODIPY unit in **4a** whereas in **4b**, it remains rotationally flexible and autonomous at room temperature. Both congeners exhibit typical S_1_ states centered on the BODIPY units and S_2_ states characterized by charge transfer (CT) properties.

## Data Availability

The data underlying this study are available in the published article and its Supporting Information.

## Supporting Information Available

Detailed experimental procedures, analytical, X-ray crystallographic, and absorption and emission data, computational details, and ^1^H, ^13^C, ^19^F, and ^11^B NMR spectra of all new compounds (PDF)
