# Supplementary material for: BODIPY-Doped Nanohoops In and Out of Conjugation
Source: Org Lett. 2025 May 5;27(19):4969–73. doi: 10.1021/acs.orglett.5c01301 (PMC12090208; doi:10.1021/acs.orglett.5c01301)
Supplement: Supplementary file 1 — ol5c01301_si_001.pdf [file ol5c01301_si_001.pdf]

Supporting Information

for

BODIPY-doped Nanohoops In and Out of Conjugation

Sebastian H. Röttger,[a,b] Pia A. Mader,[c] Heinrich F. von Köller,[b] Peter G. Jones[d] and Daniel B. Werz*[a,b]

[a] DFG Cluster of Excellence *liv*MatS @FIT and

[b] Institute of Organic Chemistry, University of Freiburg, Albertstr. 21, 79104 Freiburg, Germany

[c] Institute of Organic Chemistry, Justus Liebig University Giessen, Heinrich-Buff-Ring 17, 35392 Giessen, Germany

[d] Institute of Inorganic and Analytical Chemistry, Technical University of Braunschweig, Hagenring 30, 38106 Braunschweig, Germany

*Corresponding author: daniel.werz@chemie.uni-freiburg.de

Table of Contents

1 General Methods....S1

2 General Procedures....S2

2.1 β,β-Diiodination of BODIPYs (GP1) S2
2.2 Suzuki-Miyaura-Coupling of BODIPYs and 9-Membered Building Blocks (GP2) S3
2.3 Aromatization to BODIPY-doped Nanohoops (GP3).... S4

3 Syntheses....S5

3.1 BODIPY Syntheses.... S5
3.2 Synthesis of Borylated 9-membered Building Block 2.... S11
3.3 Synthesis of BODIPY-doped Nanohoops S12

4 NMR Spectra S14

4.1 Precursors.... S14
4.2 Cyclized Products S25

5 HR Mass Spectra S32

6 IR Spectra....S35

7 Photophysical Properties....S36

7.1 Absorption and Emission Data.... S36
7.2 Absorption and Emission Spectra.... S37

8 Crystal Structure Determinations....S41

9 Computational Studies....S46

9.1 Methods S46
9.2 Results.... S47

10 References S59

1 General Methods

All solvents were distilled before use and stored over molecular sieves unless otherwise stated. Air- and moisture-sensitive reactions were carried out in oven-dried glassware, capped with a rubber septum under atmospheric pressure of argon. Commercially available compounds were used without further purification unless otherwise stated. For all purifications by flash column chromatography (fcc), silica gel *Geduran® Si 60* (*Merck*) or *Silica 60* (*Macherey-Nagel*, both with 40 – 63 µm particle size) was used. For preparative thin layer chromatography (pTLC), pre-coated glass TLC plates (0.25 mm silica gel 60, *SIL G-25 UV_{254}*, *Macherey-Nagel*) were used. Reaction temperatures above room temperature were achieved using a silicon oil bath. To determine the reaction progress and the eluent system for purification via fcc or pTLC, aluminium TLC plates with silica matrix and a fluorescent indicator ($60F_{254}$, *Merck*) were used.
NMR spectra of proton (^{1}H), carbon (^{13}C), fluorine (^{19}F) and boron (^{11}B) were recorded on *AVIII300*, *AVIII400*, *AVIIIHD500* or *DRX500* and *Ascend™700* with a *Prodigy* CryoProbe and *AvanceNEO* console (all from *Bruker*) at room temperature with chemical shifts δ given in ppm using the residual signal from $SiMe_4$ (TMS, 0.03% in $CDCl_3$, *Deutero*) δ = 0.00 ppm as internal reference for ^{1}H and ^{13}C chemical shifts, respectively, unless otherwise stated. The following abbreviations and combinations thereof were used for ^{1}H, ^{13}C, ^{11}B and ^{19}F NMR chemical shifts: s = singlet, d = doublet, q = quartet, n = nonet, m = multiplet.
HRMS (ESI) was carried out on an *Exactive Orbitrap™* (*Thermo Scientific*) instrument.
IR spectra were recorded on a thin film spectrometer *Tensor 27* (*Bruker*) or on a Spectrum *Two FT-IR Spectrometer* (*Perkin Elmer*). The wavenumbers $\tilde{\nu}$ are given in cm^{-1}.
Melting points of solid products were recorded on a *SMP-20* (*Büchi*) or on a *MPM-HV2* (*Schorpp*) melting point meter.
UV-vis spectra were measured on a *Cary 100 Bio photometer* (*Varian*) with temperature control or on a *UV-1900i* (*Shimadzu*).
Emission spectra were measured on a *Cary Eclipse* (*Varian*) fluorescence spectrophotometer or on a *FP-8300 Spectrofluorometer* (*Jasco*). Fluorescence excitation spectra were measured but are not shown. The spectra were shown to be comparable to the UV-vis spectra of the corresponding compound. No significant discrepancy was observed. Measurements at various temperatures were performed on a *Spectrofluorometer FS5* (*Edinburgh Instruments*) with a temperature control module (*SC-26*).
Absolute fluorescence quantum yields Φ_F were determined using a *Spectrofluorometer FS5* (*Edinburgh Instruments*) equipped with an integrating sphere (*SC-30*). The corresponding sample was measured at various dilutions to approach a value without self-quenching contributions.
Literature known compounds were characterized by MS, ^{1}H NMR and melting points.
Exact reaction conditions are given in the following procedures.

2 General Procedures

2.1 β,β-Diiodination of BODIPYs (GP1)

β,β-Diiodination of BODIPYs was conducted according to a modified literature method by Zhao et al.[1]

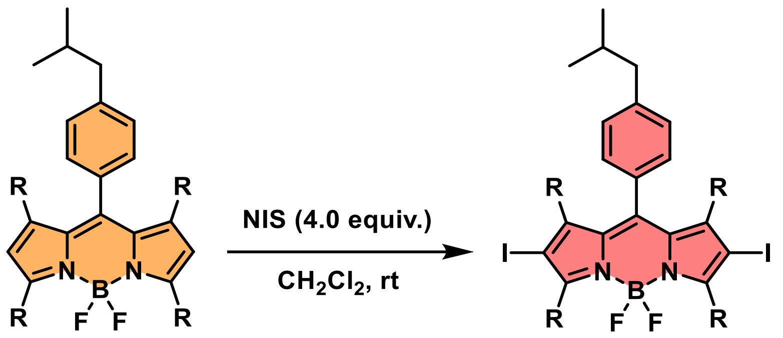

A β-unsubstituted BODIPY (1.0 equiv.) was dissolved in anhydrous CH_2Cl_2 (0.025 M) in an oven-dried round-bottom flask equipped with a rubber septum and a magnetic stirring bar. NIS (4.0 equiv.) was then added at once and the mixture was stirred at room temperature until the starting material and the mono-iodinated intermediate converted (almost) completely. The solvent was subsequently removed under reduced pressure for fcc purification.

2.2 Suzuki-Miyaura-Coupling of BODIPYs and 9-Membered Building Blocks (GP2)

Coupling of β,β-diiodinated BODIPYs and borylated C-shaped 9-membered building block (BB) was conducted according to a modified literature method by Esser et al.[2]:

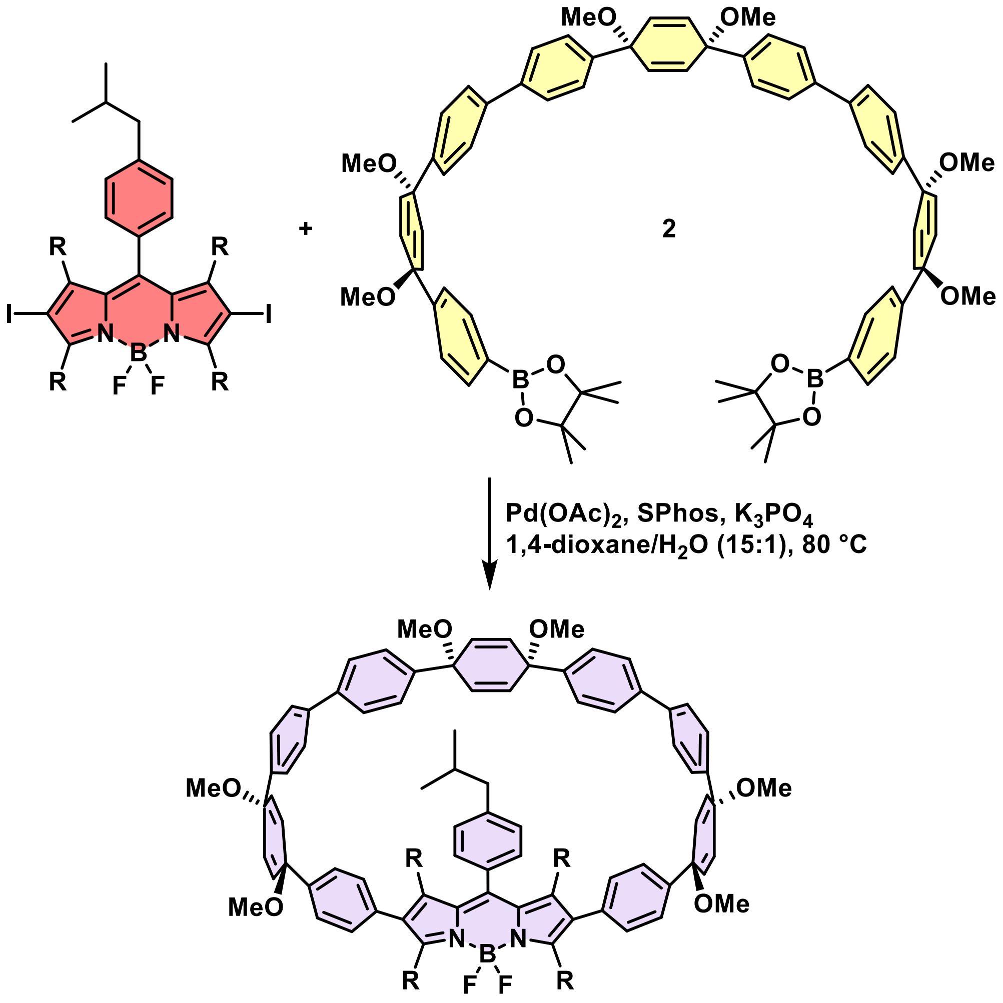

A β,β-diiodinated BODIPY (1.0 equiv.) and 9-membered BB **2** (1.0 equiv.) together with $Pd(OAc)_2$ (50 mol%), SPhos (100 mol%) and K_3PO_4 (10.0 equiv.) were dissolved in degassed 1,4-dioxane (0.0017 M) and water (7 vol.-%) in an oven-dried round-bottom flask equipped with a rubber septum and a magnetic stirring bar. The mixture was stirred at 80 °C until the starting materials converted completely. The solvent was subsequently removed under reduced pressure for a pre-column purification before the subsequent aromatization.

2.3 Aromatization to BODIPY-doped Nanohoops (GP3)

Aromatization of BODIPY-CPP precursors was conducted according to a modified literature method by Esser et al.[2]:

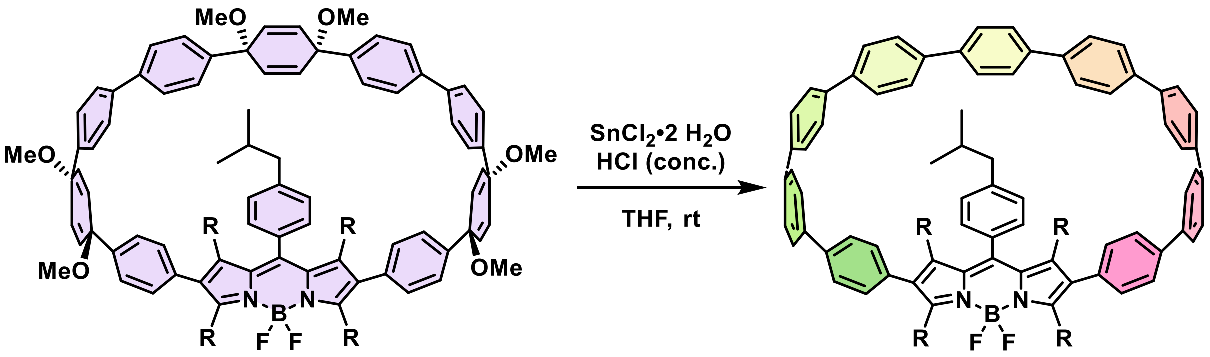

$SnCl_2$•2 H_2O (11.0 equiv.) in an oven-dried round-bottom flask equipped with rubber septum and a magnetic stirring bar was dissolved in anhydrous and unstabilized THF (0.05 M). Then conc. HCl solution (aq., 37%, 20.0 equiv.) was added and the mixture was pre-stirred for 30 min. A BODIPY-[9]CPP precursor dissolved in anhydrous and unstabilized THF (0.013 M) was then added and the mixture was stirred at room temperature until the starting material was consumed. The reaction was quenched by adding KOH (20% aq., 10 mL per mmol). The crude product was extracted with CH_2Cl_2 (3x), dried over anhydrous Na_2SO_4 and filtered. The solvent was subsequently removed under reduced pressure for purification.

Note: Although immediate product formation was observed besides several by-products (in some cases), no significant change of the reaction mixture was observed even after several days.

The following position numbers are used to assign NMR signals:

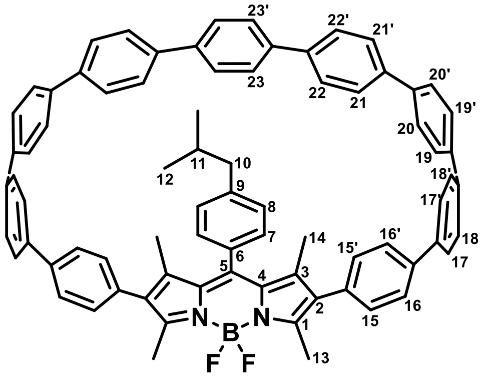

3 Syntheses

3.1 BODIPY Syntheses

1a-*H*

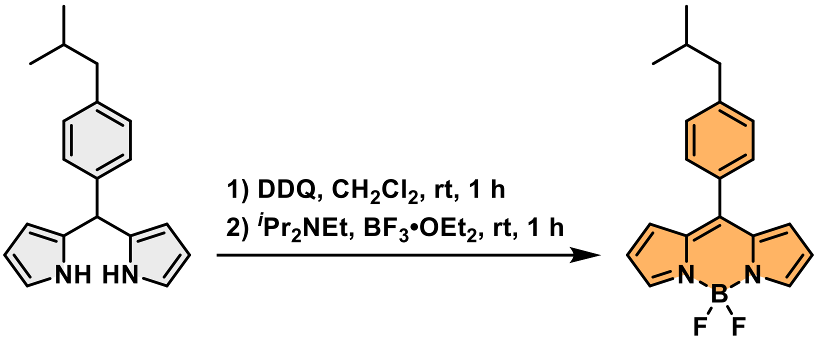

5a **1a-*H***

Dipyrromethane **5a** (1.39 g, 5.0 mmol, 1.0 equiv.) from our previous work[3] in an oven-dried round-bottom flask equipped with a rubber septum and a magnetic stirring bar was dissolved in anhydrous CH_2Cl_2 (230 mL). DDQ (1.36 g, 6.0 mmol, 1.2 equiv.) was then added and the mixture was stirred at room temperature for 1 h. Afterwards, anhydrous $^{i}Pr_2NEt$ (8.5 mL, 50.0 mmol, 10.0 equiv.) was added, followed by BF_3•OEt_2 (48% sol., 7.6 mL, 60.0 mmol, 12.0 equiv.). The reaction was then quenched with saturated aqueous $NaHCO_3$ solution, diluted with EtOAc and subsequently washed with water (3x) and finally brine. The organic phase was dried over anhydrous Na_2SO_4 and filtered. The solvent was removed under reduced pressure. Purification via fcc using *n*-pentane/CH_2Cl_2 = 2:1, then 1:1 and finally 1:2 gave BODIPY **1a-*H*** (1.44 g, 4.44 mmol, 89%) as an orange solid.

^{1}H NMR (400 MHz, $CDCl_3$): δ = 7.93 (d, J = 2.0 Hz, 2H, *H*1), 7.52 – 7.44 (m, 2H, *H*7), 7.33 – 7.28 (m, 2H, *H*8), 6.97 (d, J = 4.2 Hz, 2H, *H*3), 6.54 (dd, J = 4.4, 2.0 Hz, 2H, *H*2), 2.59 (d, J = 7.2 Hz, 2H, *H*10), 1.94 (n, J = 6.7 Hz, 1H, *H*11), 0.97 (d, J = 6.6 Hz, 6H, *H*12).

^{13}C NMR (101 MHz, $CDCl_3$): δ = 147.8 (2C), 145.2, 143.7 (2C), 134.9, 131.5 (2C), 131.3, 130.5 (2C), 129.2 (2C), 118.3 (2C), 45.3, 30.2, 22.4 (2C).

^{19}F NMR (377 MHz, $CDCl_3$): δ = −145.50 (q, $J_{B\text{-}F}$ = 28.7 Hz).

^{11}B NMR (128 MHz, $CDCl_3$): δ = 0.51 (t, $J_{F\text{-}B}$ = 28.9 Hz).

IR (Diamond ATR): $\tilde{\nu}$ [cm^{-1}] = 2958, 2917, 2862, 1537, 1470, 1383, 1257, 1108, 1067, 979, 911, 770, 745.

HRMS (ESI) *m*/*z*: $[M+Na]^+$ calcd. for $C_{19}H_{19}BF_2N_2Na^+$ 347.1502, found 347.1504.

m.p.: 84 – 86 °C

1b-*H*

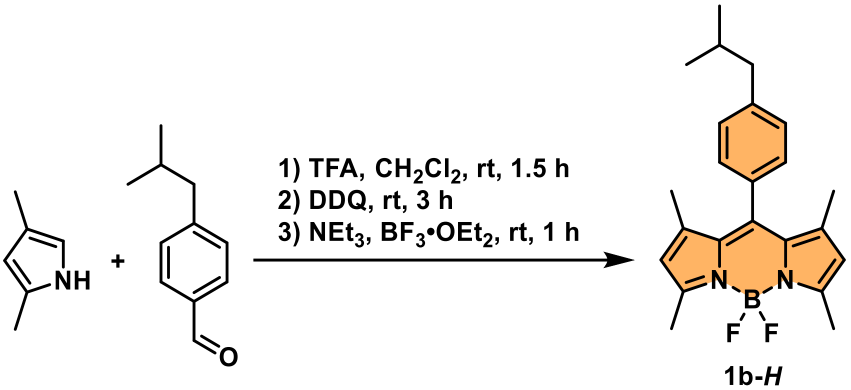

1b-*H*

Synthesis of BODIPY **1b-*H*** was conducted according the method from our previous works[4] with slight modifications:

2,4-Dimethylpyrrole (2.1 mL, 20 mmol, 2.0 equiv.) in an oven-dried round-bottom flask equipped with a rubber septum and a magnetic stirring bar was dissolved in anhydrous CH_2Cl_2 (450 mL). 4-*Iso*-butylbenzaldehyde (1.7 mL, 10 mmol, 1.0 equiv.) was then added, followed by the addition of TFA (0.1 mL, cat.) and the mixture was stirred at room temperature for 1.5 h. DDQ (2.27 g, 10 mmol, 1.0 equiv.) was then added and stirring at room temperature was continued for 3 h. Afterwards, first anhydrous NEt_3 (18.7 mL, 135 mmol, 13.5 equiv.), then BF_3•OEt_2 (48% sol., 20.3 mL, 160 mmol, 16.0 equiv.) were added. The reaction was quenched after one more hour with saturated aqueous $NaHCO_3$ solution, diluted with EtOAc and subsequently washed with water (3x) and finally brine (500 mL each). The organic phase was dried over anhydrous Na_2SO_4 and filtered. The solvent was removed under reduced pressure. Purification via fcc using *n*-pentane/CH_2Cl_2 = 2:1 gave BODIPY **1b-*H*** (1.16 g, 3.05 mmol, 31%) as an orange solid.

^{1}H NMR (300 MHz, $CDCl_3$): δ = 7.27 – 7.22 (m, 2H, *H*7), 7.19 – 7.14 (m, 2H, *H*8), 5.97 (s, 2H, *H*2), 2.58 – 2.51 (m, 8H, *H*14 + *H*2), 1.90 (n, *J* = 6.8 Hz, 1H, *H*11), 1.39 (s, 6H, *H*13), 0.90 (d, *J* = 6.6 Hz, 6H, *H*12).

m.p.: 139 – 142 °C

1a

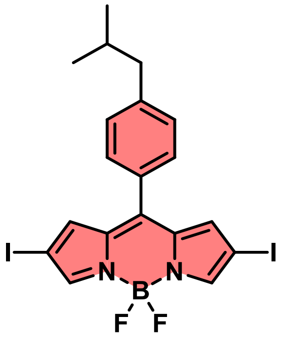

BODIPY **1a-*H*** (1.16 g, 3.58 mmol, 1.0 equiv.) was converted with NIS (3.22 g, 14.31 mmol, 4.0 equiv.) in anhydrous CH_2Cl_2 (120 mL) according to **GP1** over one week to give β,β-diiodinated BODIPY **1a** (1.46 g, 2.53 mmol, 71%) as a fluffy bright red solid after fcc purification using *n*-pentane/PhMe = 5:1, then 4:1 and finally 3:1.

^{1}H NMR (700 MHz, $CDCl_3$): δ = 7.87 (d, J = 1.7 Hz, 2H, *H*1), 7.48 – 7.43 (m, 2H, *H*7), 7.35 – 7.30 (m, 2H, *H*8), 7.13 (d, J = 1.5 Hz, 2H, *H*3), 2.59 (d, J = 7.2 Hz, 2H, *H*10), 1.96 (n, J = 6.5 Hz, 1H, *H*11), 0.98 (d, J = 6.6 Hz, 6H, *H*12).

^{13}C NMR (176 MHz, $CDCl_3$): δ = 148.1 (2C), 146.3, 146.2, 146.2 (2C), 137.7, 136.0 (2C), 130.5 (2C), 130.5 (2C), 129.6 (2C), 45.3, 30.2, 22.4 (2C).

^{19}F NMR (659 MHz, $CDCl_3$): δ = −144.80 (q, $J_{B\text{-}F}$ = 28.1 Hz).

^{11}B NMR (225 MHz, $CDCl_3$): δ = −0.30 (t, $J_{F\text{-}B}$ = 28.1 Hz).

IR (Diamond ATR): $\tilde{\nu}$ [cm^{-1}] = 3115, 2949, 2916, 2861, 2161, 1545, 1464, 1333, 1246, 1102, 1061, 975, 891, 838, 745, 701, 613, 570.

HRMS (ESI) *m/z*: $[M+Na]^+$ calcd. for $C_{19}H_{17}BF_2I_2N_2Na^+$ 598.9434, found 598.9438.

m.p.: 170 – 174 °C

1b

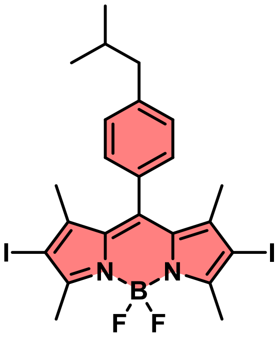

BODIPY **1b-*H*** (761 mg, 2.00 mmol, 1.0 equiv.) from our previous work[4] was converted with NIS (1.80 g, 8.00 mmol, 4.0 equiv.) in anhydrous CH_2Cl_2 (80 mL) according to **GP1** over 2 h to give β,β-diiodinated BODIPY **1b** (1.13 g, 1.79 mmol, 89%) as a red solid with a green luster after fcc purification using *n*-pentane/CH_2Cl_2 = 5:1, then 3:1.

^{1}H NMR (400 MHz, $CDCl_3$): δ = 7.30 – 7.26 (m, 2H, *H*7), 7.17 – 7.09 (m, 2H, *H*8), 2.64 (s, 6H, *H*14), 2.57 (d, *J* = 7.2 Hz, 2H, *H*10), 1.92 (n, *J* = 6.8 Hz, 1H, *H*11), 1.41 (s, 6H, *H*13), 0.91 (d, *J* = 6.6 Hz, 6H, *H*12).

^{13}C NMR (101 MHz, $CDCl_3$): δ = 156.5 (2C), 145.4 (2C), 143.3 (2C), 141.9, 132.0 (2C), 131.5, 130.2 (2C), 127.5 (2C), 85.5, 45.2, 30.2, 22.2 (2C), 16.9 (2C), 16.0 (2C).

^{19}F NMR (377 MHz, $CDCl_3$): δ = −146.07 (q, $J_{B\text{-}F}$ = 32.2 Hz).

^{11}B NMR (128 MHz, $CDCl_3$): δ = 0.80 (t, $J_{F\text{-}B}$ = 32.2 Hz).

IR (Diamond ATR): $\tilde{\nu}$ [cm^{-1}] = 2953, 2919, 2113, 1525, 1394, 1341, 1300, 1172, 1083, 990, 787, 700, 578.

HRMS (ESI) *m/z*: $[M+Na]^+$ calcd. for $C_{23}H_{25}BF_2I_2N_2Na^+$ 655.0060, found 655.0063.

m.p.: 170 – 173 °C

1a-*Br*

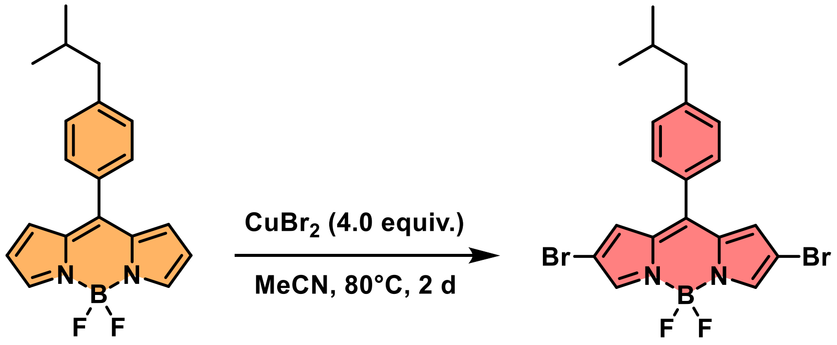

1a-*H* **1a-*Br***

Synthesis of β,β-dibrominated BODIPY **1a-*Br*** was conducted according to a modified literature method by Jiao et al.[5]:

BODIPY **1a-*H*** (1.44 g, 4.44 mmol, 1.0 equiv.) in an oven-dried round-bottom flask equipped with a rubber septum and a magnetic stirring bar was dissolved in anhydrous MeCN (220 mL). Anhydrous $CuBr_2$ (3.97 g, 17.76 mmol, 4.0 equiv.) was added and the reaction mixture was stirred at 80 °C for 2 days and cooled down afterwards since the starting material and mono-brominated intermediate showed (almost) full conversion. The solvent was subsequently re-moved under reduced pressure. Purification via fcc using *n*-pentane/CH_2Cl_2 = 3:1 and finally 2:1 gave β,β-dibrominated BODIPY **1a-*Br*** (1.45 g, 3.01 mmol, 68%) as a red solid.

^{1}H NMR (300 MHz, $CDCl_3$): δ = 7.87 – 7.80 (m, 2H, *H*1), 7.50 – 7.43 (m, 2H, *H*7), 7.37 – 7.30 (m, 2H, *H*8), 7.01 – 6.97 (m, 2H, *H*3), 2.60 (d, *J* = 7.2 Hz, 2H, *H*10), 1.96 (n, *J* = 6.7 Hz, 1H, *H*11), 0.97 (d, *J* = 6.6 Hz, 6H, *H12*).

^{13}C NMR (76 MHz, $CDCl_3$): δ = 147.5, 146.3 (2C), 143.8 (2C), 134.6, 131.7 (2C), 130.5 (2C), 130.4 (2C), 129.6 (2C), 107.0, 45.3, 30.2, 22.4 (2C).

^{19}F NMR (282 MHz, $CDCl_3$): δ = −145.47 (q, $J_{B\text{-}F}$ = 28.0 Hz).

^{11}B NMR (96 MHz, $CDCl_3$): δ = 0.05 (t, $J_{F\text{-}B}$ = 27.9 Hz).

IR (Diamond ATR): $\tilde{\nu}$ [cm^{-1}] = 3120, 2954, 2920, 1609, 1550, 1475, 1376, 1348, 1251, 1093, 1072, 1021, 1003, 987, 932, 910, 837, 797, 746, 708.

HRMS (ESI) *m*/*z*: $[M-F]^+$ calcd. for $C_{19}H_{17}B^{79}Br^{81}BrFN_2^+$ 462.9810, found 462.9813.

m.p.: 182 – 184 °C

1b-*Br*

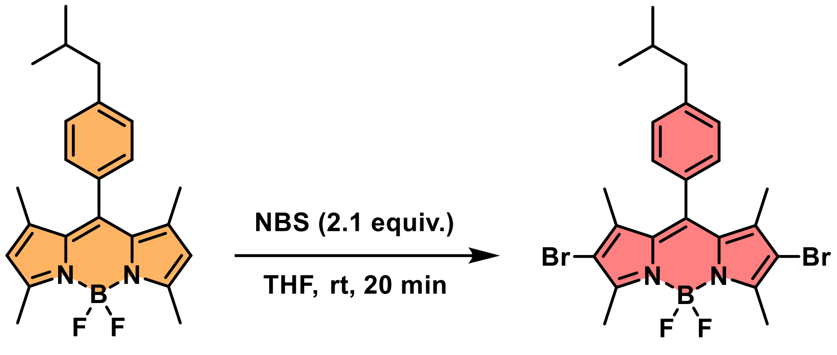

1b-*H* **1b-*Br***

Synthesis of β,β-dibrominated BODIPY **1b-*Br*** was conducted according to a modified literature method by Bañuelos, Peña-Cabrera et al.[6]:

BODIPY **1b-*H*** (1.14 g, 3.00 mmol, 1.0 equiv.) from our previous work[4a,4c] in an oven-dried round-bottom flask equipped with a rubber septum and a magnetic stirring bar was dissolved in anhydrous THF (16 mL). NBS (1.12 g, 6.3 mmol, 2.1 equiv.) in an oven-dried pear-shaped flask was dissolved in anhydrous THF (8 mL) and then added dropwise within 5 min to the first solution under stirring at room temperature. The reaction was quenched with water after 20 min since the starting material and mono-brominated intermediate showed (almost) full conversion. The crude product was washed once again with water and finally brine (25 mL each), dried over anhydrous Na_2SO_4 and filtered. The solvent was subsequently removed under reduced pressure. Purification via fcc using *n*-pentane/CH_2Cl_2 = 5:1, then 3:1 and finally 2:1 gave β,β-dibrominated BODIPY **1b-*Br*** (1.19 g, 2.21 mmol, 74%) as a red crystalline solid.

^{1}H NMR (500 MHz, $CDCl_3$): δ = 7.30 – 7.27 (m, 2H, *H*7), 7.16 – 7.12 (m, 2H, *H*8), 2.60 (s, 6H, *H*14), 2.58 (d, *J* = 7.3 Hz, 2H, *H*10), 1.90 (n, *J* = 6.8 Hz, 1H, *H*11), 1.39 (s, 6H, *H*13), 0.91 (d, *J* = 6.6 Hz, 6H, *H*12).

^{13}C NMR (126 MHz, $CDCl_3$): δ = 153.7 (2C), 143.4 (2C), 142.6, 140.6 (2C), 131.7 (2C), 130.5, 130.1 (2C), 127.5 (2C), 111.7, 45.2, 30.2, 22.2 (2C), 13.6 (2C), 13.6 (2C).

^{19}F NMR (471 MHz, $CDCl_3$): δ = −146.50 (q, $J_{B\text{-}F}$ = 32.5 Hz).

^{11}B NMR (161 MHz, $CDCl_3$): δ = 0.72 (t, $J_{F\text{-}B}$ = 32.2 Hz).

IR (Diamond ATR): $\tilde{\nu}$ [cm^{-1}] = 2961, 2917, 1610, 1533, 1460, 1397, 1347, 1305, 1260, 1173, 1085, 991, 916, 802, 746, 702, 591.

HRMS (ESI) *m/z*: $[M+Na]^+$ calcd. for $C_{23}H_{25}B^{79}Br^{81}BrF_2N_2Na^+$ 561.0317, found 561.0321.

m.p.: 170 – 172 °C

3.2 Synthesis of Borylated 9-membered Building Block 2

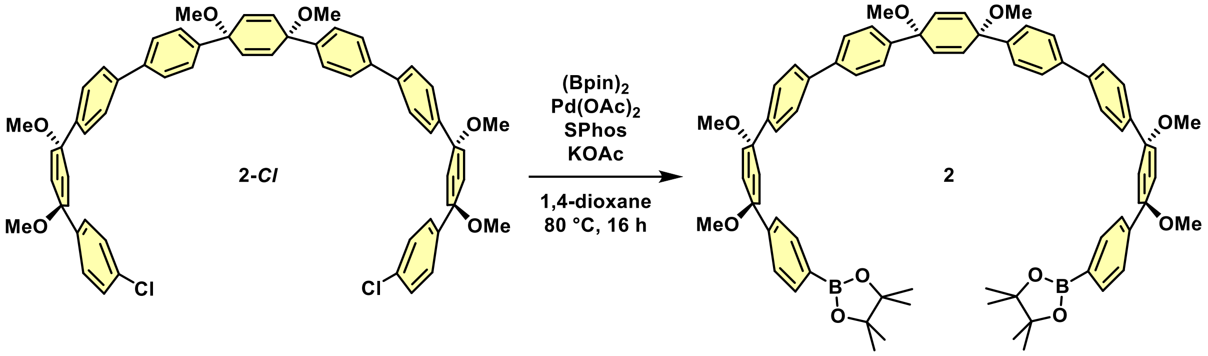

Synthesis of dichlorinated 9-membered BB **2-*Cl*** was conducted according to a modified literature method by Liu et al.[7] using flow reactors.[8]

KOAc (954 mg, 9.72 mmol, 6.0 equiv.) was flame-dried in a round-bottom flask and cooled under nitrogen. Bis(pinacolato)diborane (1.23 g, 4.83 mmol, 3.0 equiv.), dichlorinated 9-membered BB **2-*Cl*** (1.50 g, 1.60 mmol, 1.0 equiv.), $Pd(OAc)_2$ (37 mg, 162 µmol, 10 mol%) and SPhos (174 mg, 416 µmol, 26 mol%) were added to the flask, which was then evacuated and backfilled with nitrogen in three cycles. Anhydrous 1,4-dioxane (36 mL) was added to the flask via a syringe. The reaction mixture was heated to 80 °C and stirred for 16 h. The reaction mixture was then cooled to room temperature. Active carbon was added and the mixture was again stirred for 15 min. Subsequently, the mixture was filtered through a Celite® plug and rinsed with EtOAc. The filtrate was concentrated under reduced pressure. The resulting solid was sonicated with methanol and filtered. The crude product was then filtered through a silica plug using EtOAc and concentrated again to give diborylated 9-membered BB **2** (1.73 g, 1.54 mmol, 96%) as an off-white solid.

^{1}H NMR (300 MHz, $CDCl_3$): δ = 7.80 – 7.72 (m, 4H, aryl B-C-C*H*), 7.60 – 7.37 (m, 20H, aryl *H*), 6.20 – 6.05 (m, 12H, olefinic *H*), 3.48 – 3.41 (m, 18H, OCH_3), 1.31 (s, 24H, Bpin-CH_3).

m.p.: 152 – 155 °C

3.3 Synthesis of BODIPY-doped Nanohoops

4a

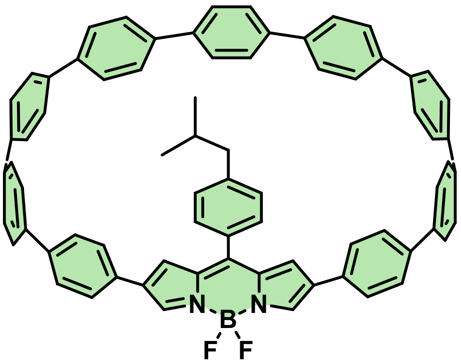

β,β-Diiodinated BODIPY **1a** (29 mg, 50 µmol, 1.0 equiv.) was converted with borylated 9-membered BB **2** (56 mg, 50 µmol, 1.0 equiv.) together with $Pd(OAc)_2$ (5.6 mg, 25 µmol, 50 mol%), SPhos (21 mg, 50 µmol, 100 mol%) and K_3PO_4 (106 mg, 0.5 mmol, 10.0 equiv.) in 1,4-dioxane (30 mL) and water (2 mL) were converted according to **GP2** over 16 h to give BODIPY-[9]CPP precursor **3a** (52 mg) as a purple solid after pre-column purification using CH_2Cl_2/EtOAc = 50:1 (**HRMS** (ESI) *m*/*z*: $[M+H]^+$ calcd. for $C_{79}H_{72}BF_2N_2O_6^+$ 1193.5446, found 1193.5462). Without any further purification, this was then converted together with $SnCl_2$•2 H_2O (124 mg, 550 µmol, 11.0 equiv.) and conc. HCl (aq., 37%, 84 µL, 20.0 equiv.) in THF (10 mL in total) according to **GP3** to give BODIPY-[9]CPP **4a** (6.5 mg, 6.5 µmol, 13% over 2 steps) as a dark green solid after pTLC purification using *n*-pentane/PhMe = 3:1 up to pure PhMe.

^{1}H NMR (700 MHz, $CDCl_3$): δ = 8.15 (s, 2H, *H*1), 7.63 – 7.51 (m, 32H, *H*16/16' – *H*23/23'), 7.49 – 7.46 (m, 2H, *H*7), 7.46 – 7.43 (m, 4H, *H*15/15'), 7.34 – 7.29 (m, 2H, *H*8), 6.90 (s, 2H, *H*3), 2.61 (d, *J* = 7.2 Hz, 2H, *H*10), 1.99 (n, *J* = 6.6 Hz, 1H, *H*11), 1.00 (d, *J* = 6.6 Hz, 6H, *H*12).

^{13}C NMR (176 MHz, $CDCl_3$): δ = 148.2, 145.1, 142.9, 138.6, 138.4, 138.4, 138.3, 138.3, 138.2, 136.8, 131.5, 131.4, 131.2, 130.2, 129.3, 127.5, 127.5, 127.4, 127.4, 127.4, 127.3, 127.1, 126.0, 125.8, 45.4, 30.2, 22.5.

^{19}F NMR (659 MHz, $CDCl_3$): δ = −142.34 (dq, $^2J_{F\text{-}B\text{-}F}$ = 91.7 Hz, $^1J_{F\text{-}B}$ = 29.4 Hz), −163.29 (dq, $^2J_{F\text{-}B\text{-}F}$ = 94.3 Hz, $^1J_{F\text{-}B}$ = 23.8 Hz).

^{11}B NMR (225 MHz, $CDCl_3$): δ = 0.37 – −0.07 (m).

IR (Diamond ATR): $\tilde{\nu}$ [cm^{-1}] = 3025, 2921, 2850, 1716, 1552, 1526, 1483, 1304, 1230, 1181, 1120, 1095, 1058, 981, 934, 909, 807, 745, 716.

HRMS (ESI) *m*/*z*: $[M-F]^+$ calcd. for $C_{73}H_{53}BFN_2^+$ 987.4280, found 987.4272.

m.p.: 183 – 186 °C

4b

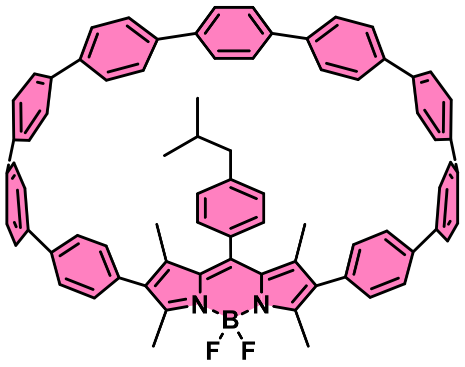

β,β-Diiodinated BODIPY **1b** (316 mg, 500 µmol, 1.0 equiv.) was converted with borylated 9-membered BB **2** (563 mg, 500 µmol, 1.0 equiv.) together with $Pd(OAc)_2$ (56 mg, 250 µmol, 50 mol%), SPhos (205 mg, 500 µmol, 100 mol%) and K_3PO_4 (1.06 g, 5.0 mmol, 10.0 equiv.) in 1,4-dioxane (300 mL) and water (20 mL) were converted according to **GP2** over 16 h to give BODIPY-[9]CPP precursor **3b** (275 mg) as a pink solid after pre-column purification using CH_2Cl_2/EtOAc = 30:1 (**HRMS** (ESI) *m*/*z*: $[M-F]^+$ calcd. for $C_{83}H_{79}BFN_2O_6^+$ 1229.6010, found 1229.5996). Without any further purification, this was then converted together with $SnCl_2$•2 H_2O (1.24 g, 5.5 mmol, 11.0 equiv.) and conc. HCl (aq., 37%, 917 µL, 20.0 equiv.) in THF (180 mL in total) according to **GP3** to give BODIPY-[9]CPP **4b** (45 mg, 42 µmol, 8% over 2 steps) as a magenta solid after pTLC purification using *n*-pentane/CH_2Cl_2 = 2:1.

^{1}H NMR (700 MHz, $CDCl_3$): δ = 7.69 – 7.67 (m, 4H), 7.66 – 7.64 (m, 4H), 7.62 – 7.59 (m, 8H), 7.58 – 7.55 (m, 8H), 7.54 – 7.52 (m, 4H), 7.45 – 7.41 (m, 4H), 7.26 – 7.24 (m, 2H, *H*7), 7.23 – 7.20 (m, 2H, *H*8), 7.14 – 7.10 (m, 4H), 2.54 (d, *J* = 7.3 Hz, 2H, *H*10), 2.37 (s, 6H, *H*14), 1.89 (n, *J* = 6.6 Hz, 1H, *H*11), 1.54 (s, 6H, *H*13), 0.90 (d, *J* = 6.6 Hz, 6H, *H*12).

^{13}C NMR (176 MHz, $CDCl_3$): δ = 155.4, 143.0, 142.9, 139.4, 139.1, 138.8, 138.7, 138.4, 138.4, 138.0, 138.0, 132.5, 132.4, 132.3, 132.2, 130.8, 129.8, 128.2, 128.0, 127.7, 127.6, 127.4, 127.4, 127.2, 127.2, 127.1, 45.3, 30.3, 22.3, 14.2, 14.1.

^{19}F NMR (659 MHz, $CDCl_3$): δ = −141.00 – −148.00 (m).

^{11}B NMR (225 MHz, $CDCl_3$): δ = 1.10 (t, $J_{F\text{-}B}$ = 32.2 Hz).

IR (Diamond ATR): $\tilde{\nu}$ [cm^{-1}] = 3024, 2955, 2922, 2851, 1903, 1724, 1594, 1513, 1485, 1456, 1385, 1311, 1261, 1230, 1166, 1097, 1069, 1000, 808, 736, 704, 687.

HRMS (ESI) *m*/*z*: $[M+H]^+$ calcd. for $C_{77}H_{62}BF_2N_2^+$ 1063.4969, found 1063.4977.

m.p.: 237 – 240 °C

4 NMR Spectra

4.1 Precursors

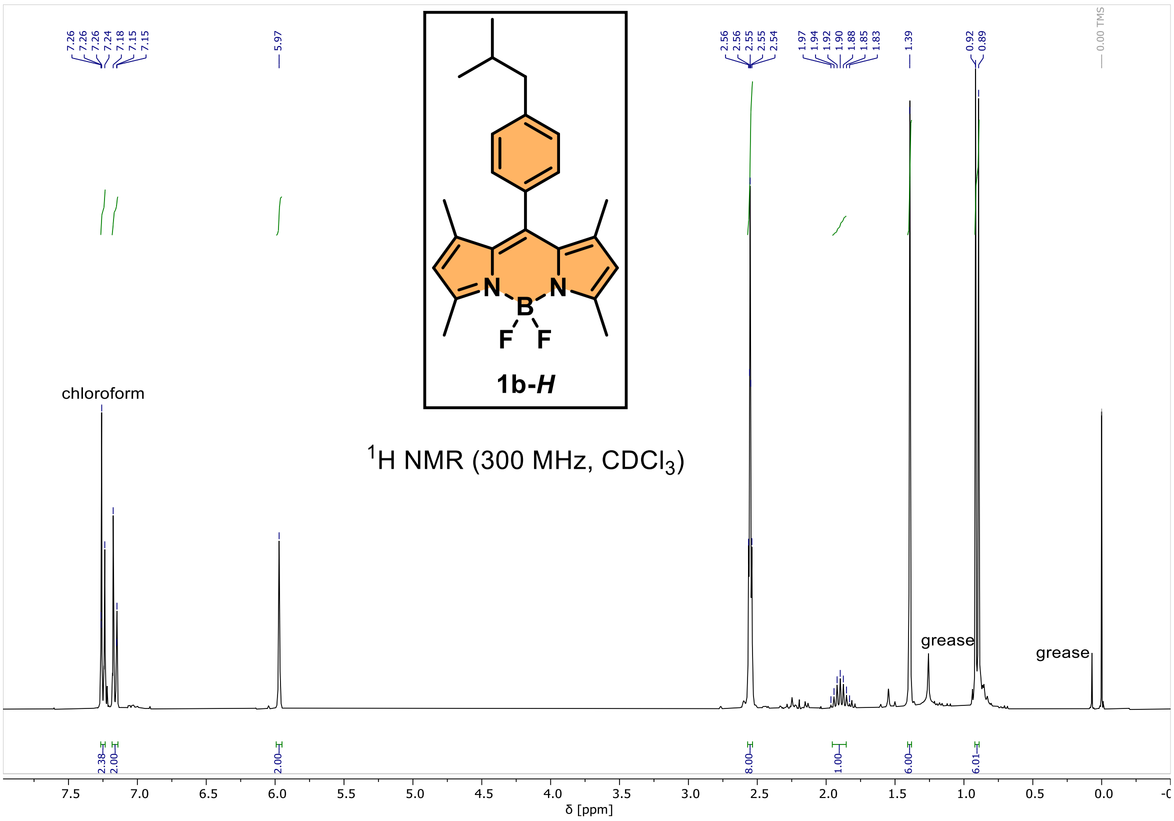

Figure S1: ^{1}H NMR spectrum (300 MHz) of **1b-*H*** in $CDCl_3$ at room temperature.

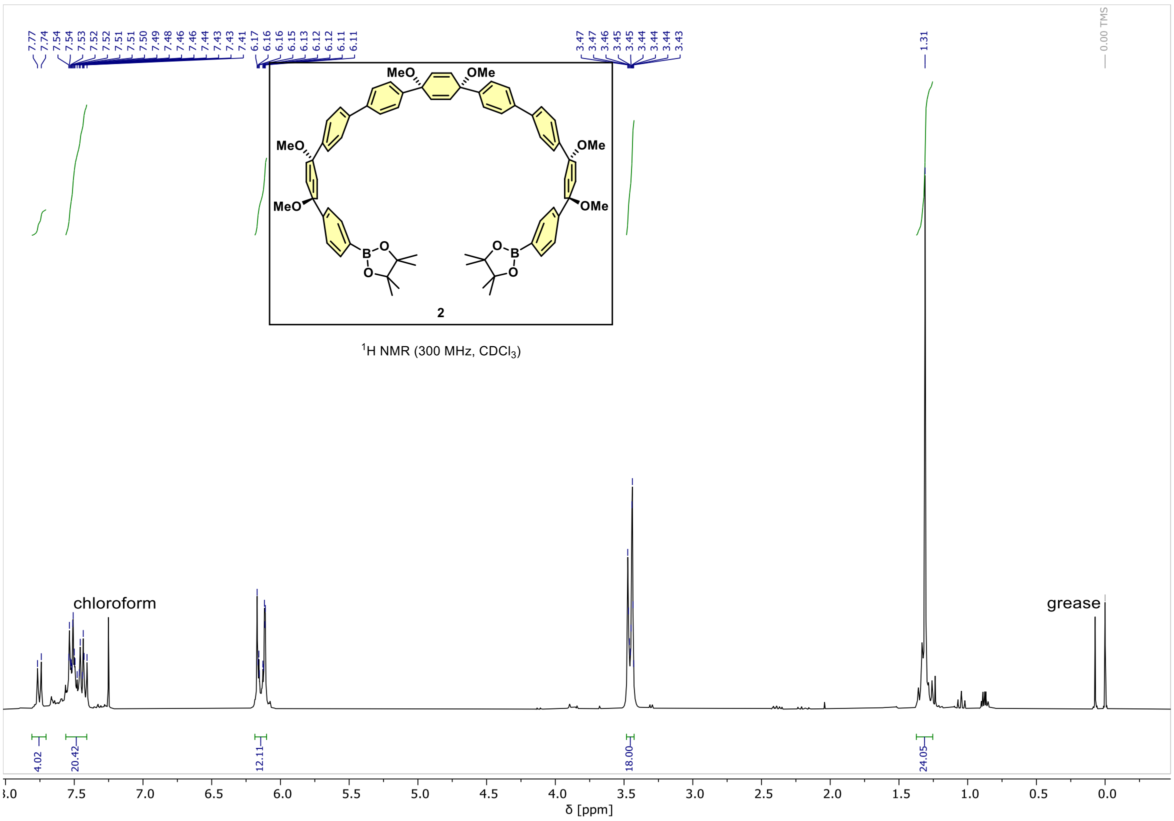

Figure S2: ^{1}H NMR spectrum (300 MHz) of **2** in $CDCl_3$ at room temperature.

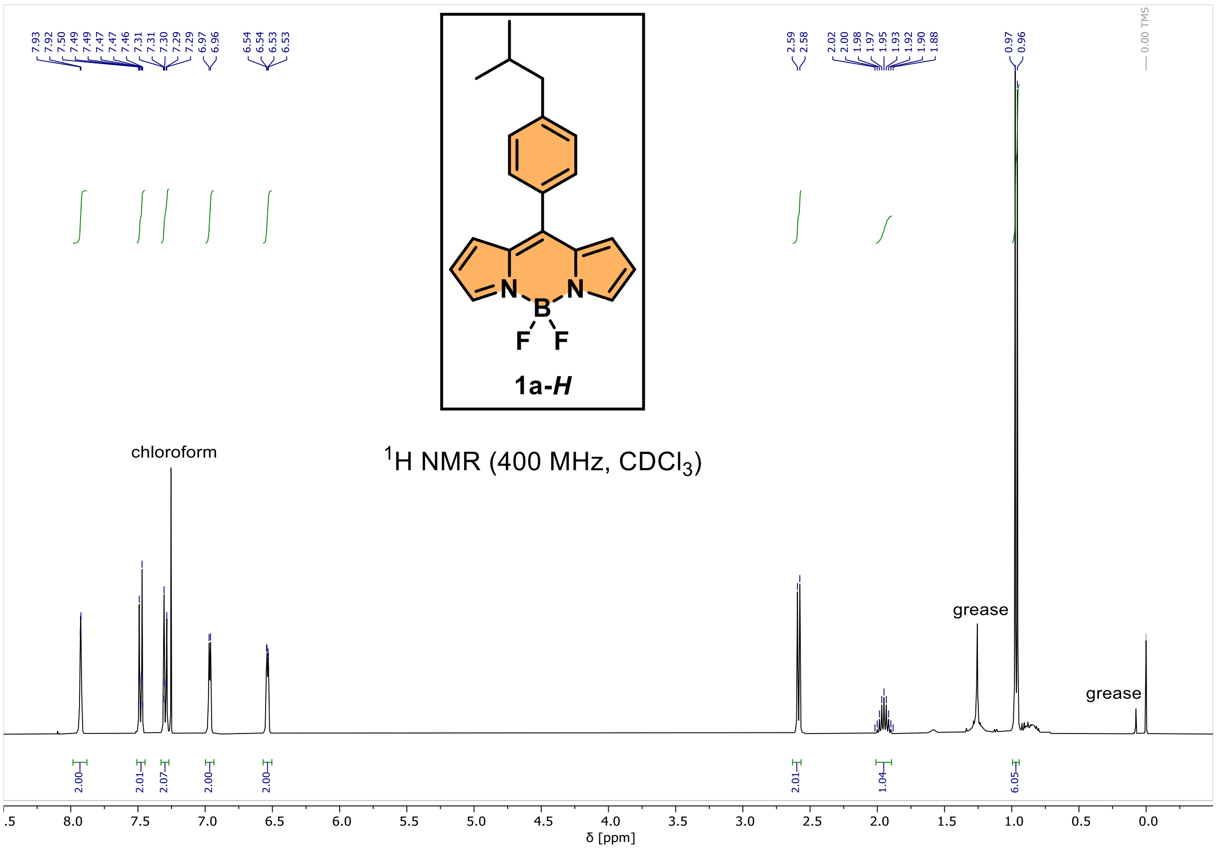

Figure S3: ^{1}H NMR spectrum (400 MHz) of **1a-*H*** in CDCl$_3$ at room temperature.

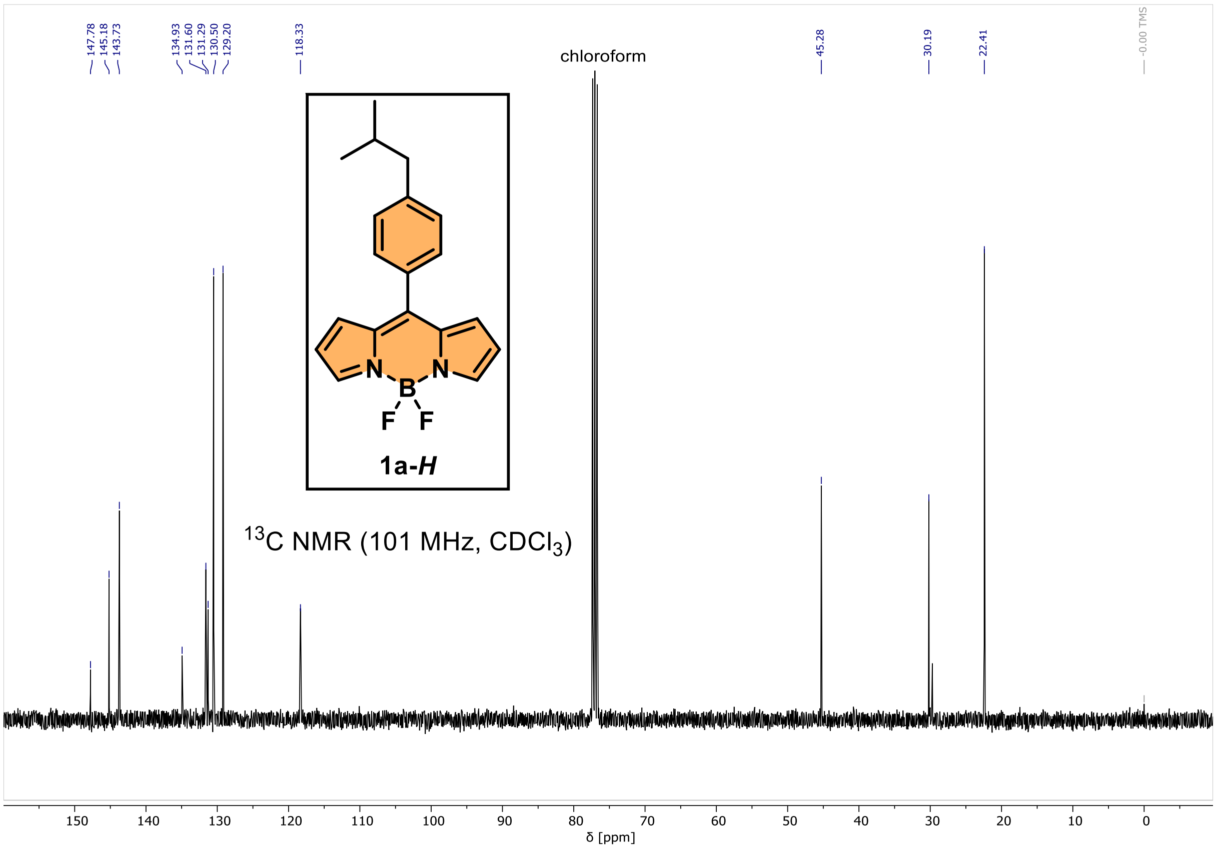

Figure S4: ^{13}C NMR spectrum (101 MHz) of **1a-*H*** in CDCl$_3$ at room temperature.

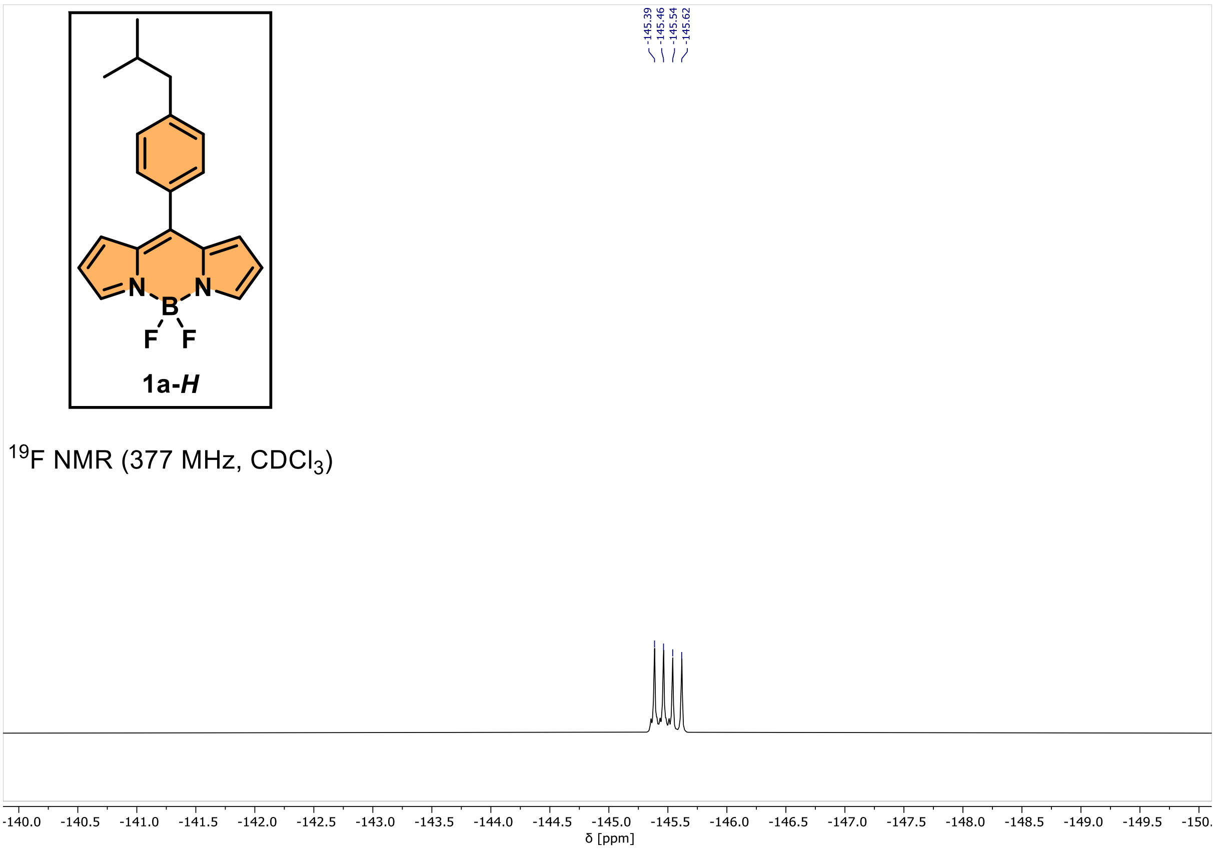

Figure S5: ^{19}F NMR spectrum (377 MHz) of **1a-*H*** in $CDCl_3$ at room temperature.

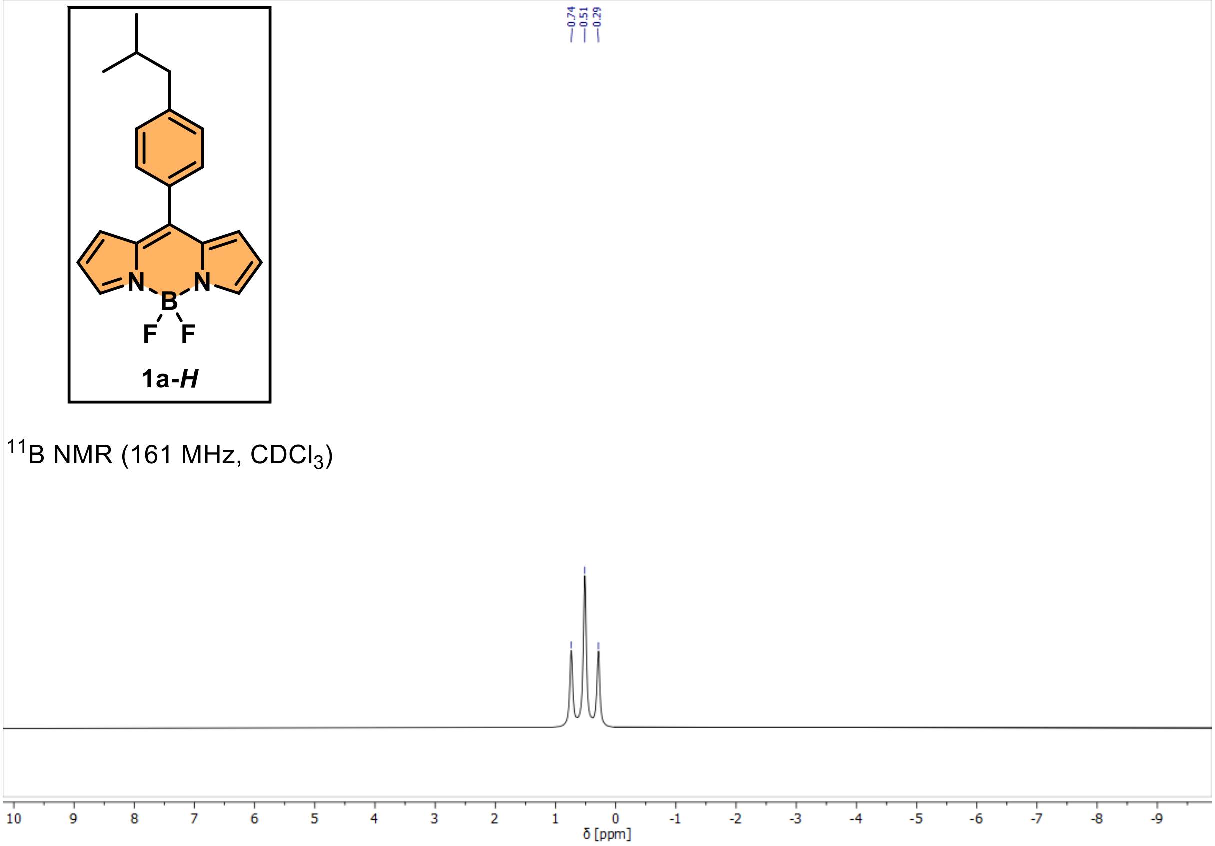

Figure S6: ^{11}B NMR spectrum (161 MHz) of **1a-*H*** in $CDCl_3$ at room temperature.

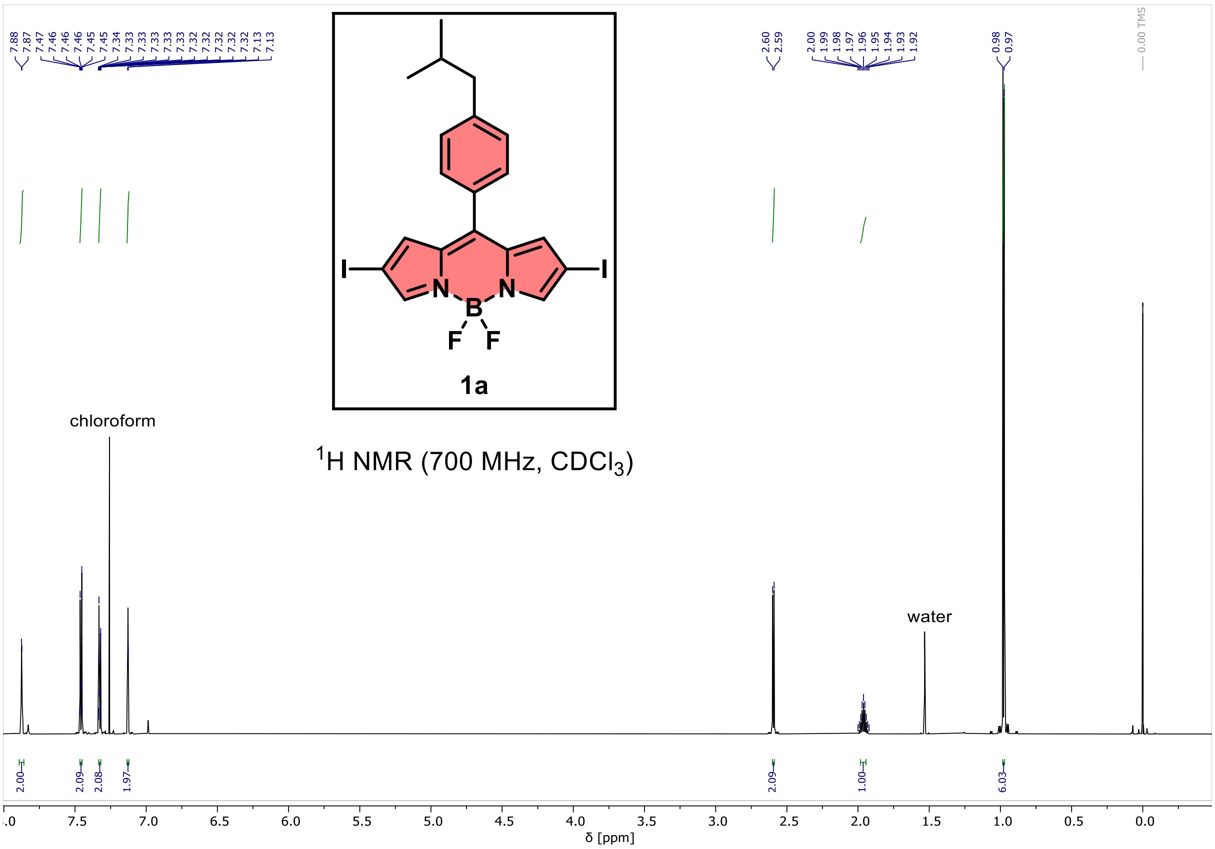

Figure S7: ^{1}H NMR spectrum (700 MHz) of **1a** in $CDCl_3$ at room temperature.

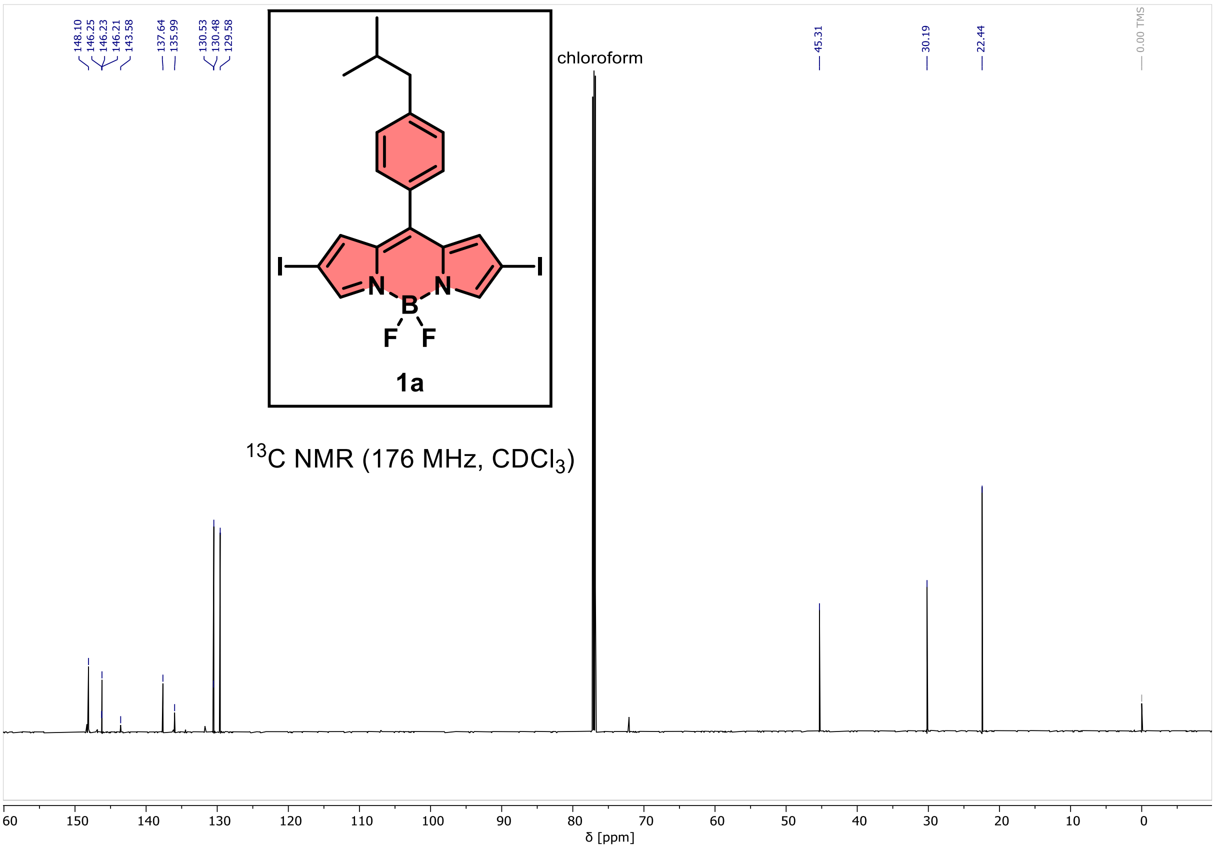

Figure S8: ^{13}C NMR spectrum (176 MHz) of **1a** in $CDCl_3$ at room temperature.

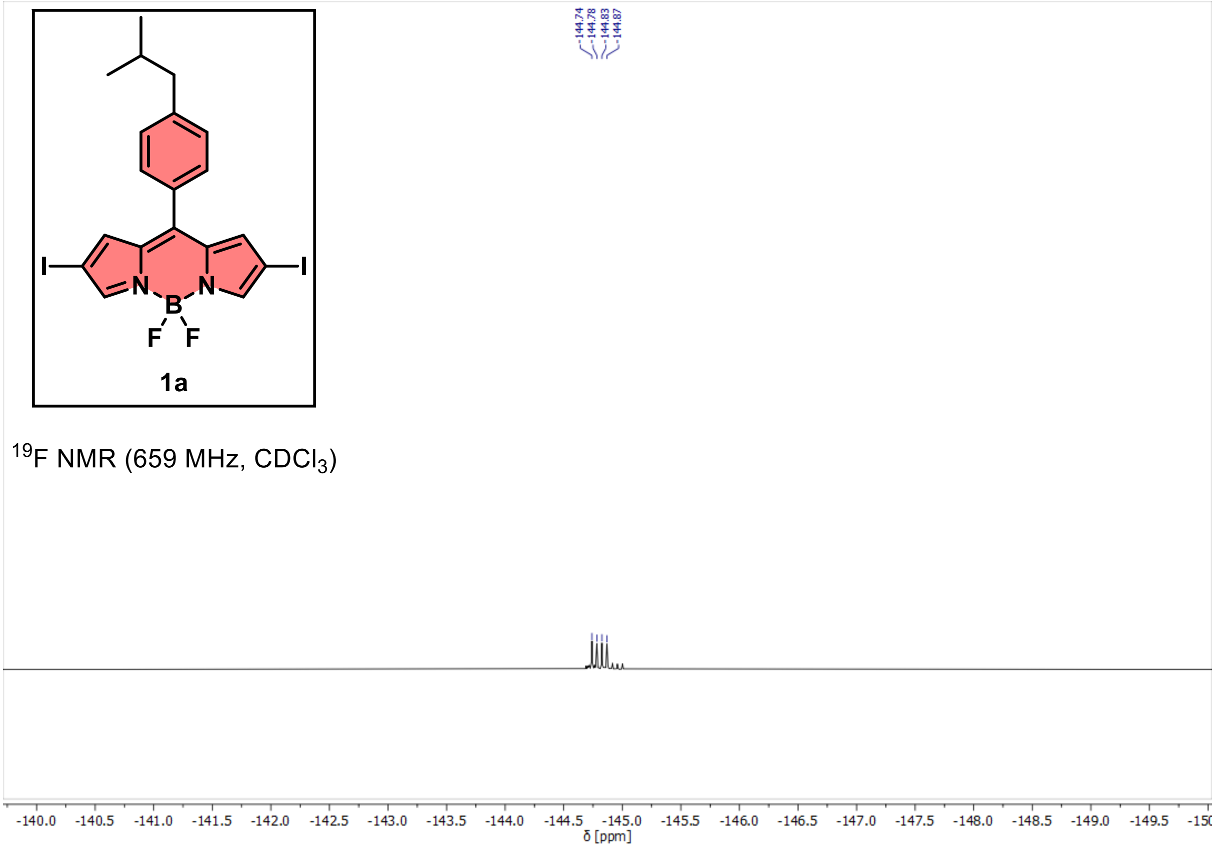

Figure S9: ^{19}F NMR spectrum (659 MHz) of **1a** in $CDCl_3$ at room temperature.

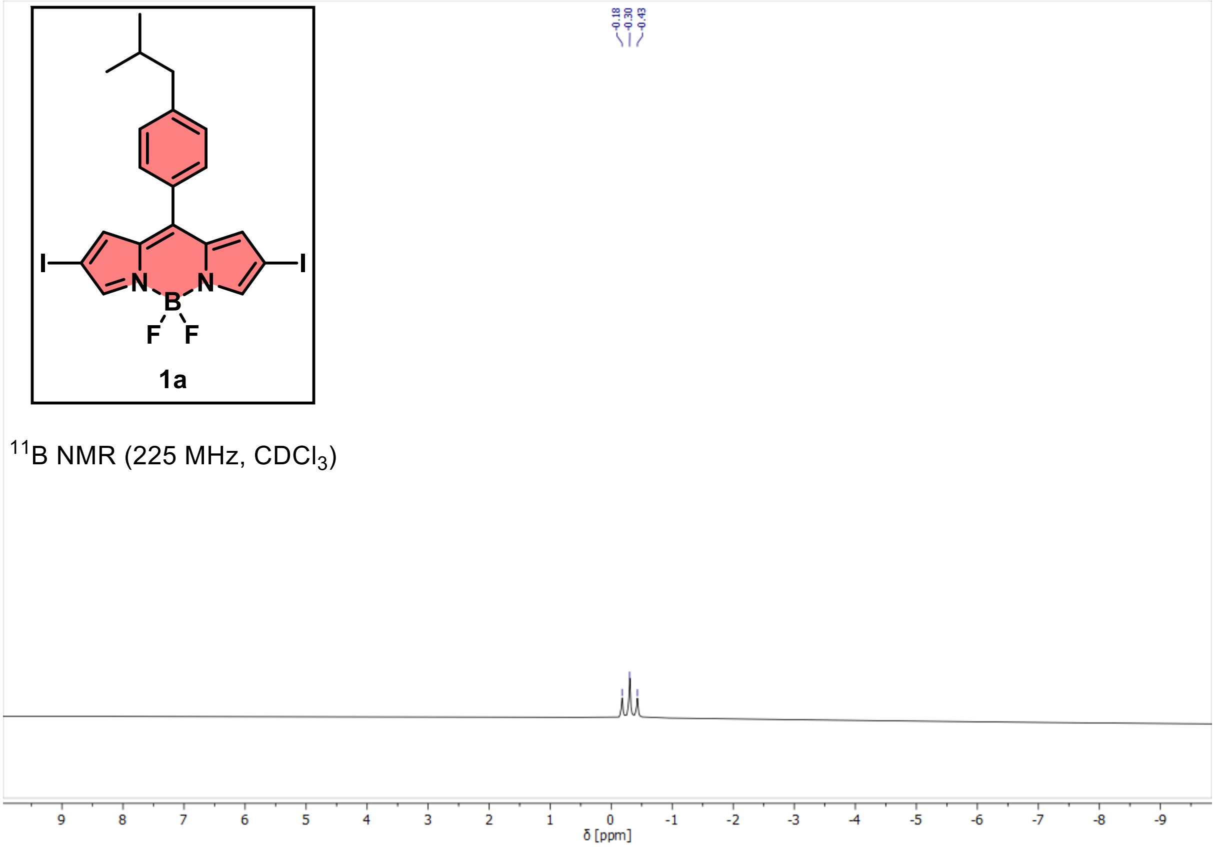

Figure S10: ^{11}B NMR spectrum (225 MHz) of **1a** in $CDCl_3$ at room temperature.

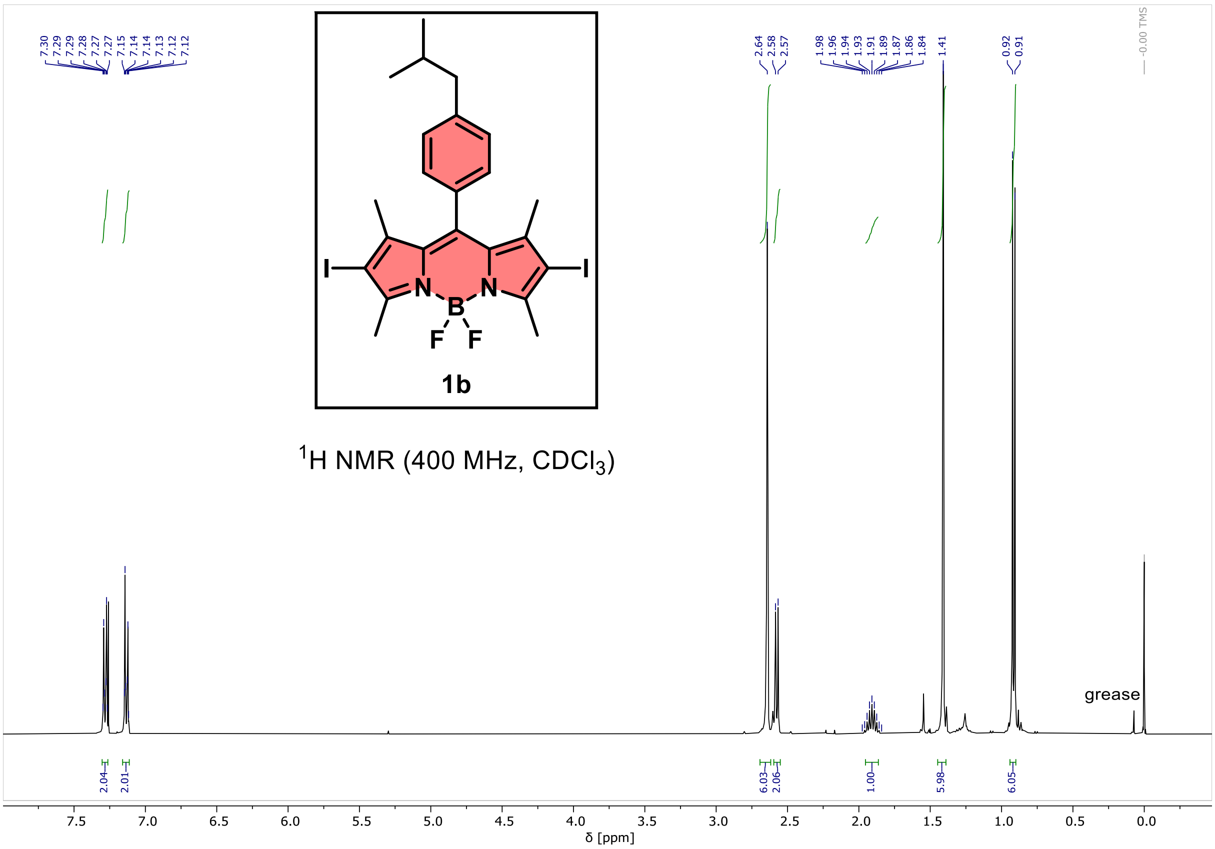

Figure S11: ^{1}H NMR spectrum (400 MHz) of **1b** in $CDCl_3$ at room temperature.

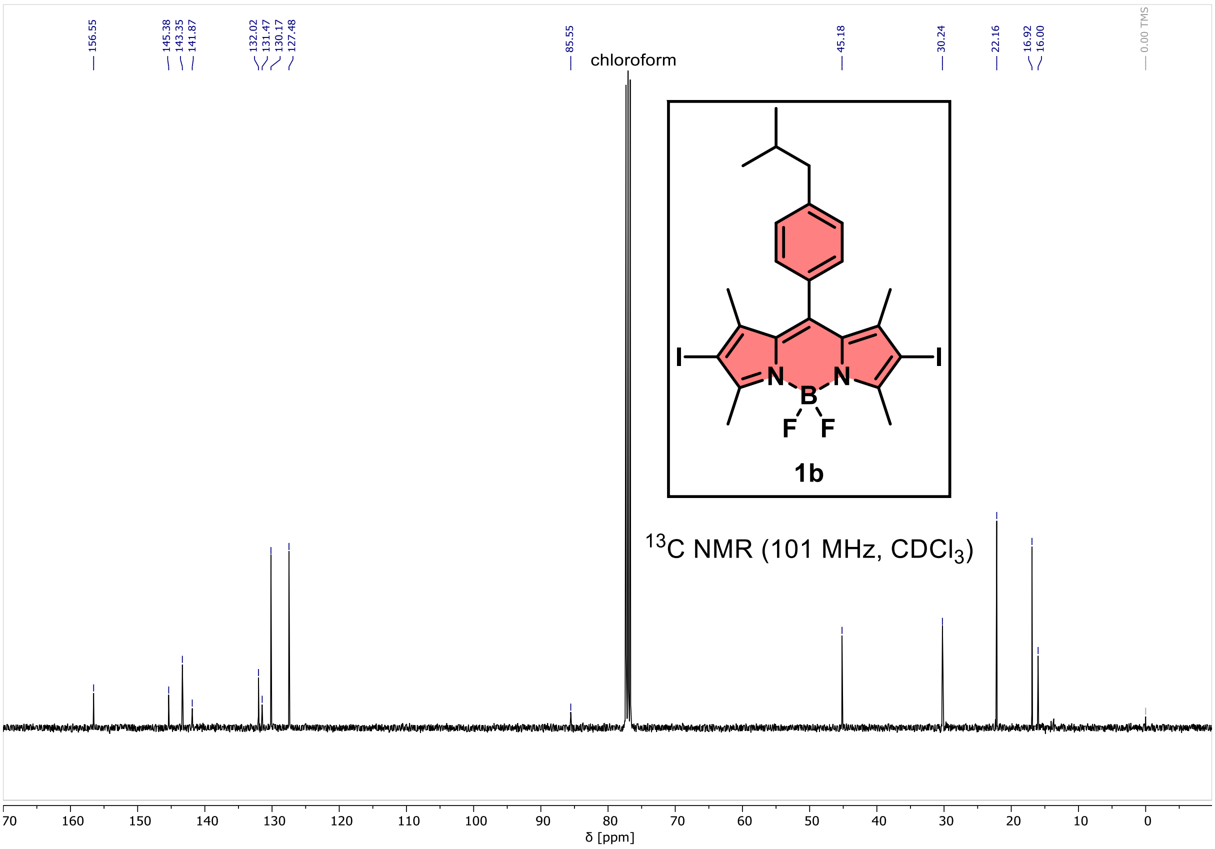

Figure S12: ^{13}C NMR spectrum (101 MHz) of **1b** in $CDCl_3$ at room temperature.

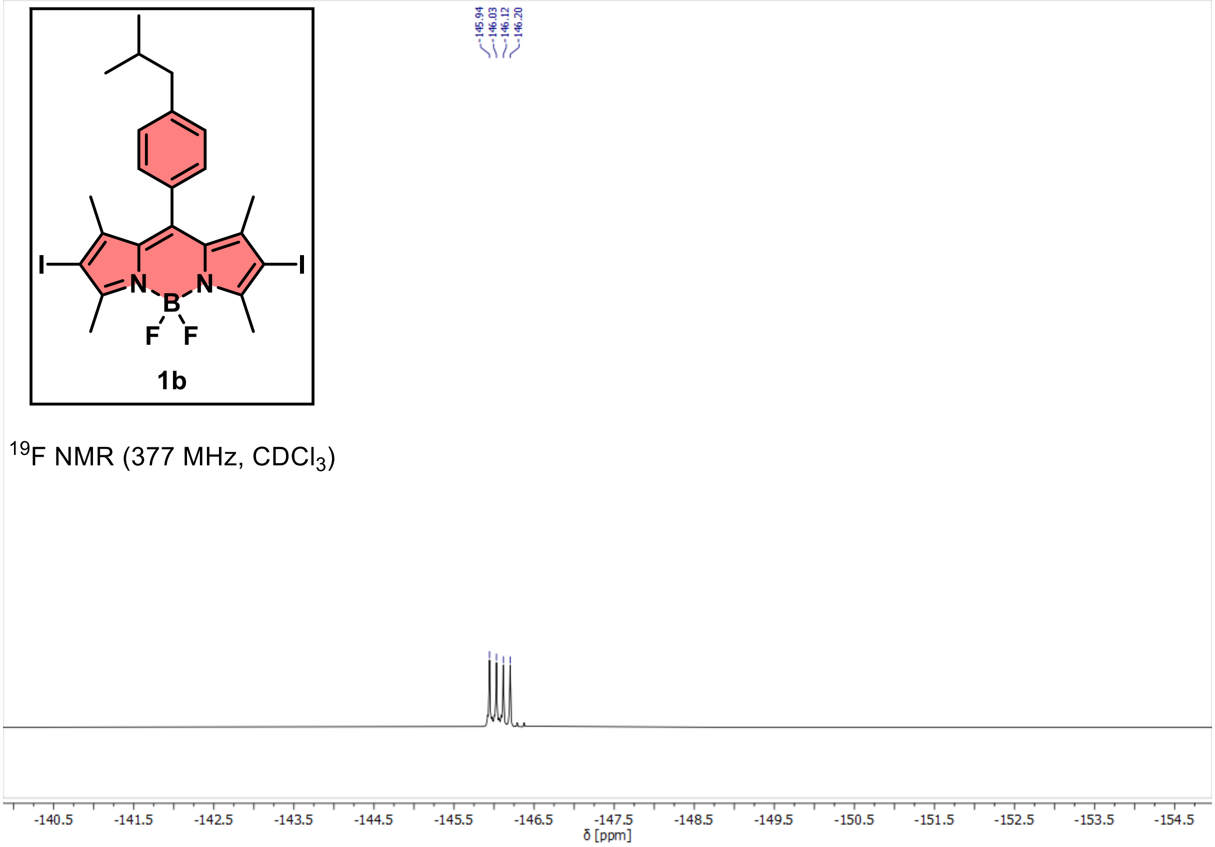

Figure S13: ^{19}F NMR spectrum (377 MHz) of **1b** in $CDCl_3$ at room temperature.

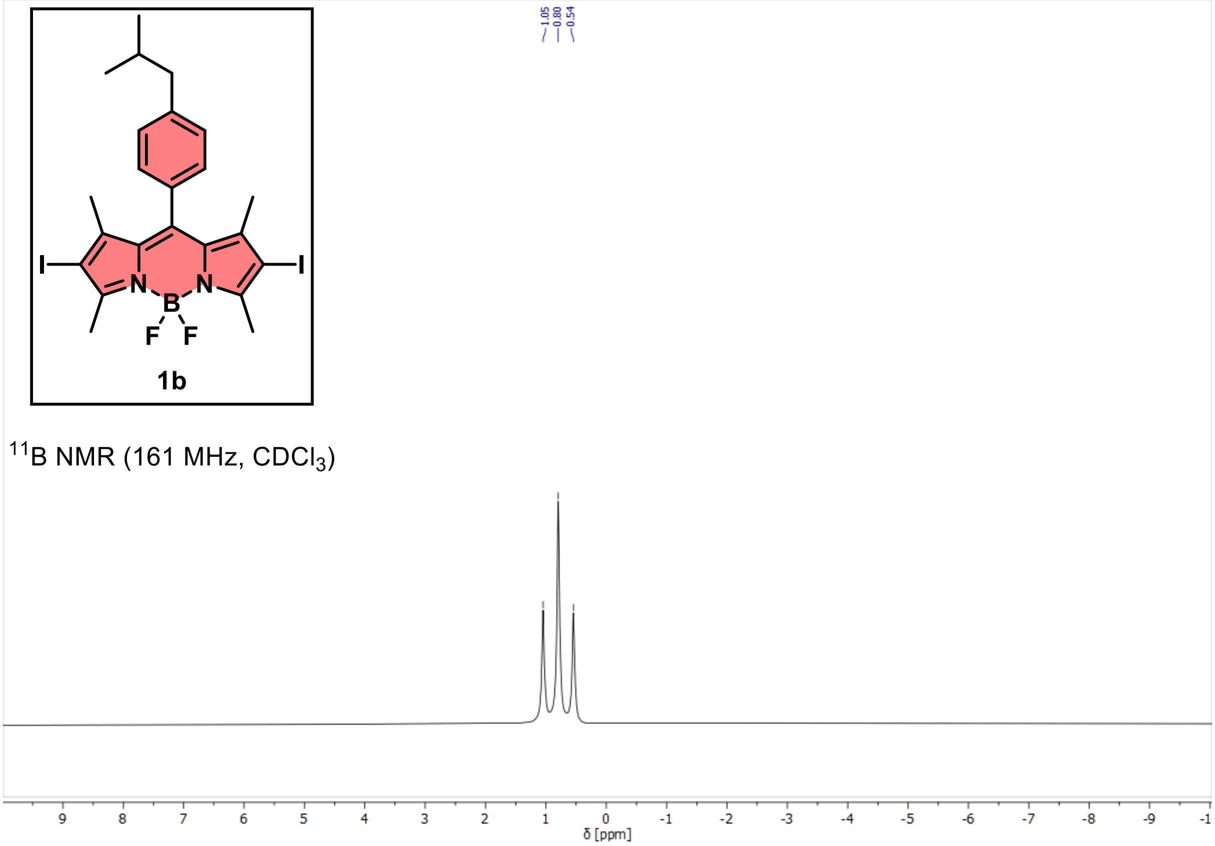

Figure S14: ^{11}B NMR spectrum (161 MHz) of **1b** in $CDCl_3$ at room temperature.

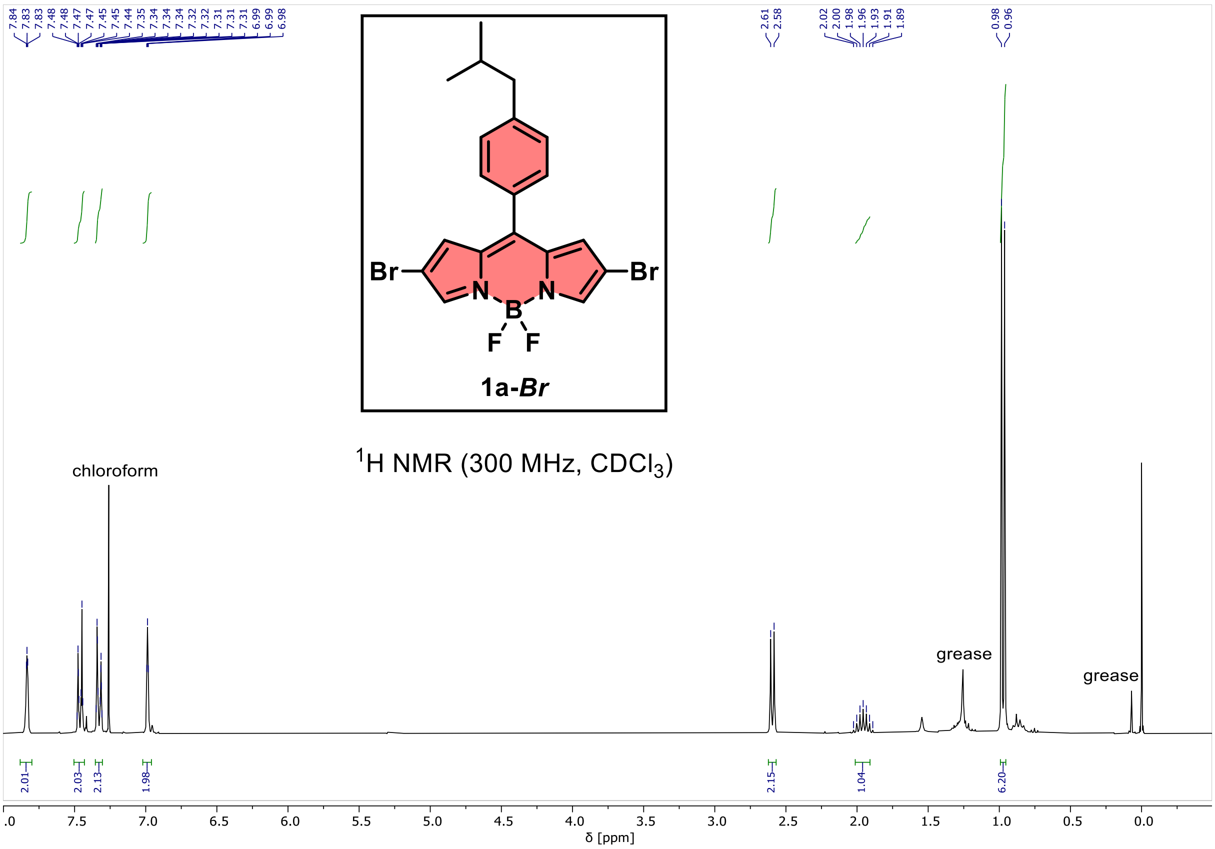

Figure S15: ^{1}H NMR spectrum (300 MHz) of **1a-*Br*** in $CDCl_3$ at room temperature.

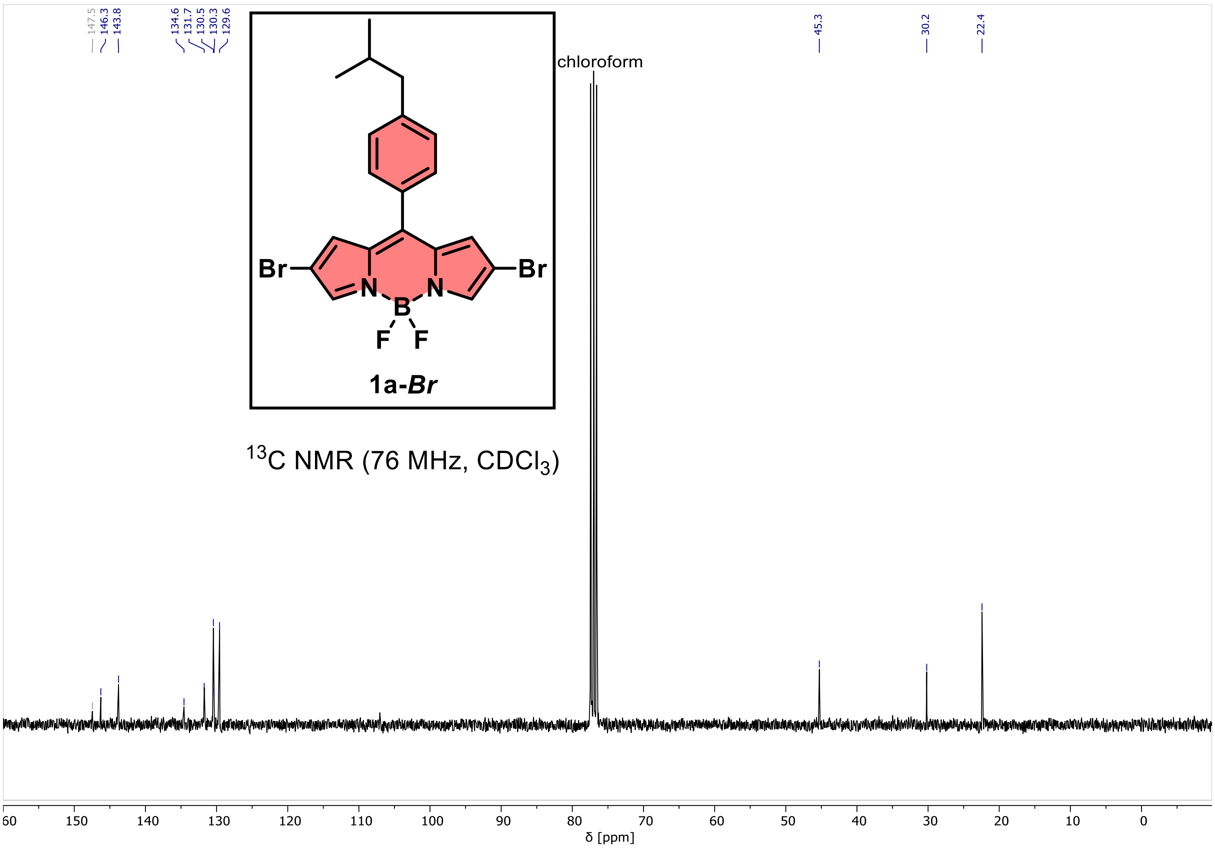

Figure S16: ^{13}C NMR spectrum (76 MHz) of **1a-*Br*** in $CDCl_3$ at room temperature.

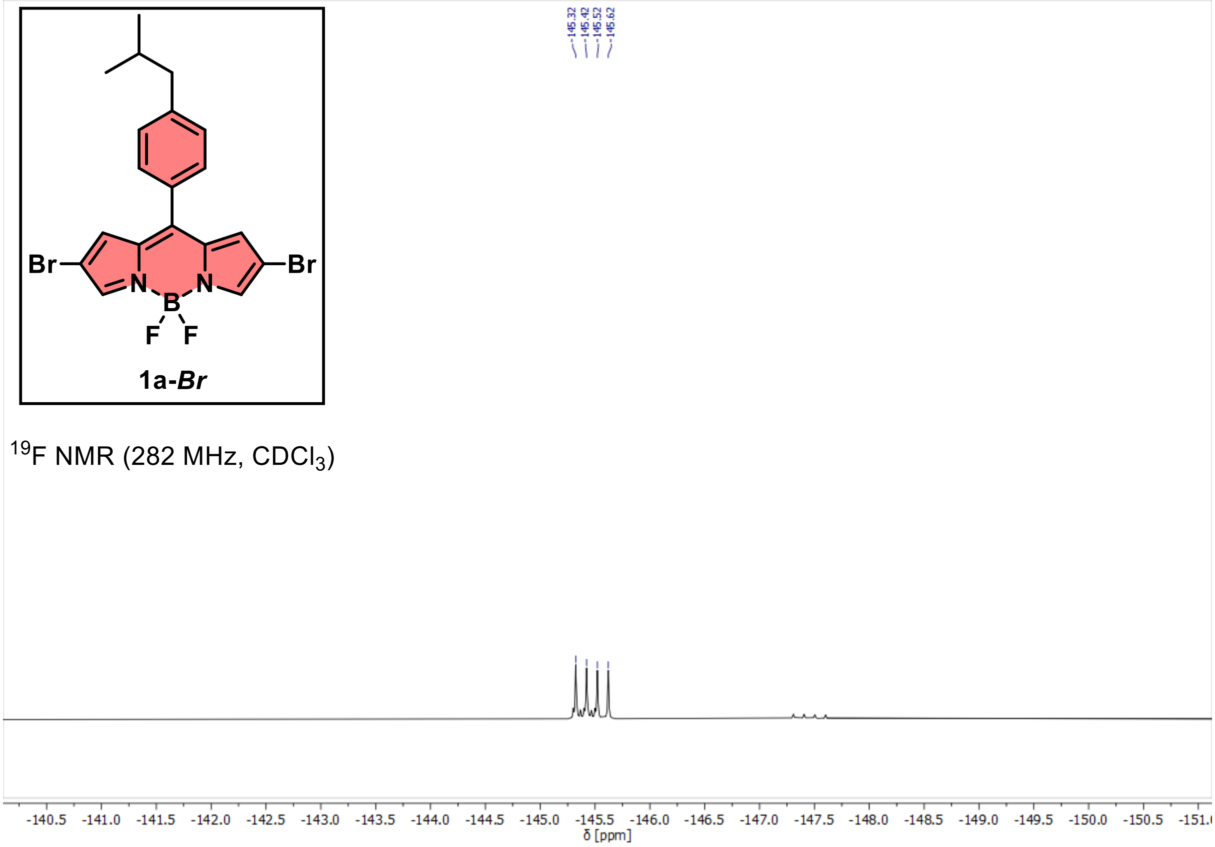

Figure S17: ^{19}F NMR spectrum (282 MHz) of **1a-*Br*** in $CDCl_3$ at room temperature.

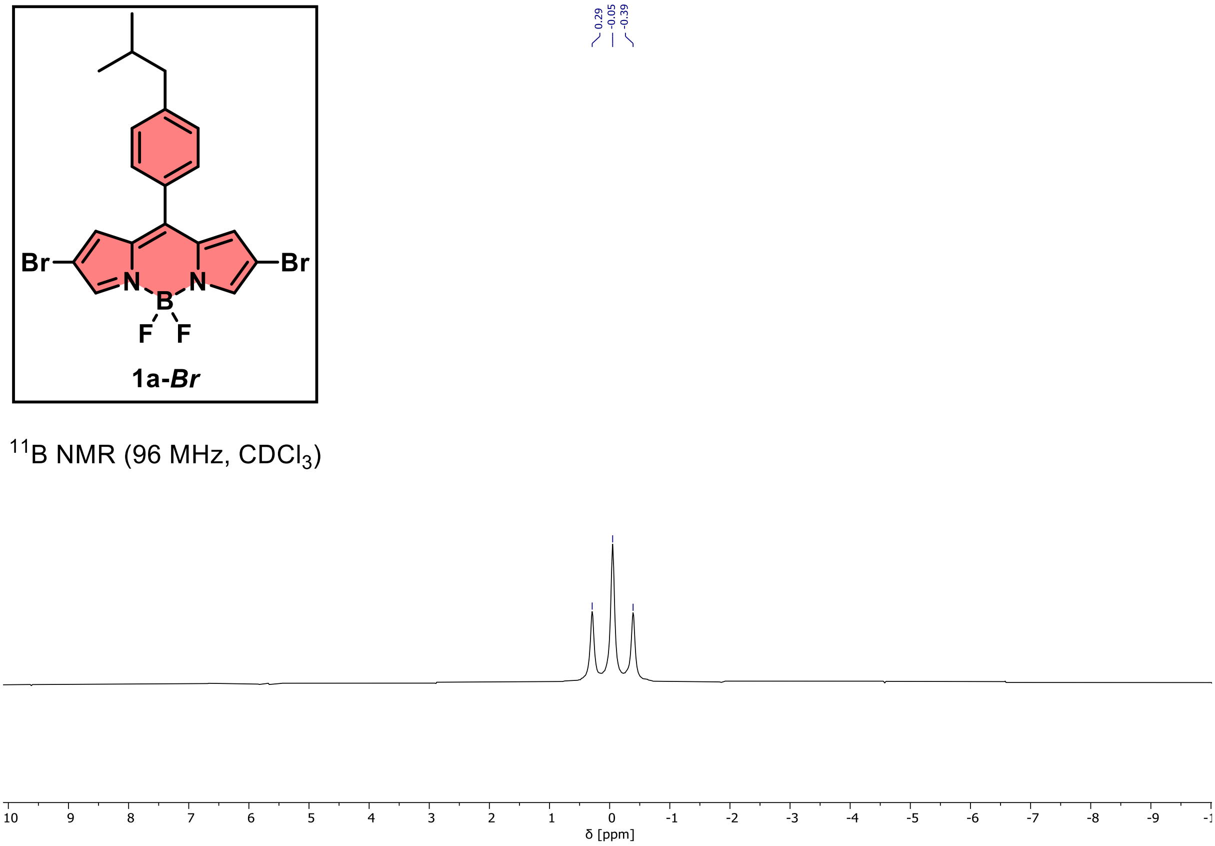

Figure S18: ^{11}B NMR spectrum (96 MHz) of **1a-*Br*** in $CDCl_3$ at room temperature.

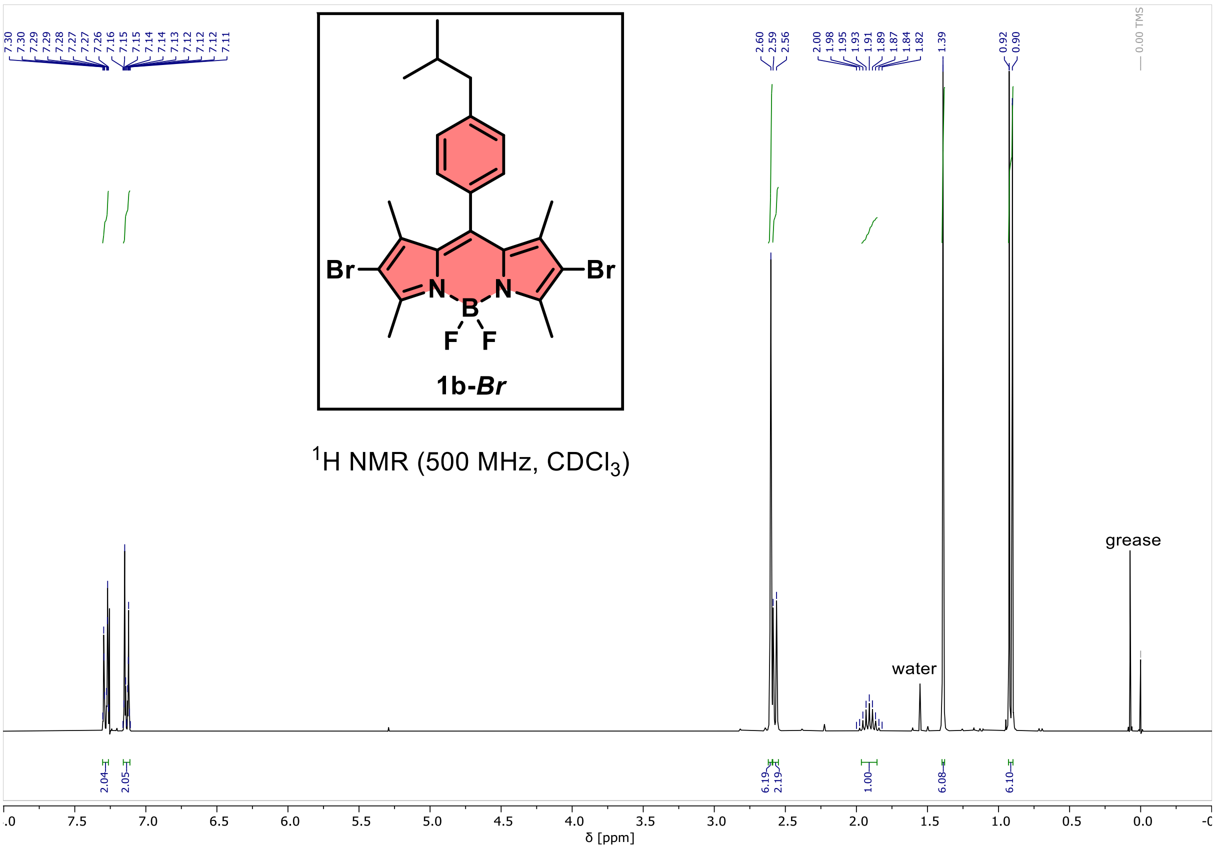

Figure S19: ^{1}H NMR spectrum (500 MHz) of **1b-*Br*** in $CDCl_3$ at room temperature.

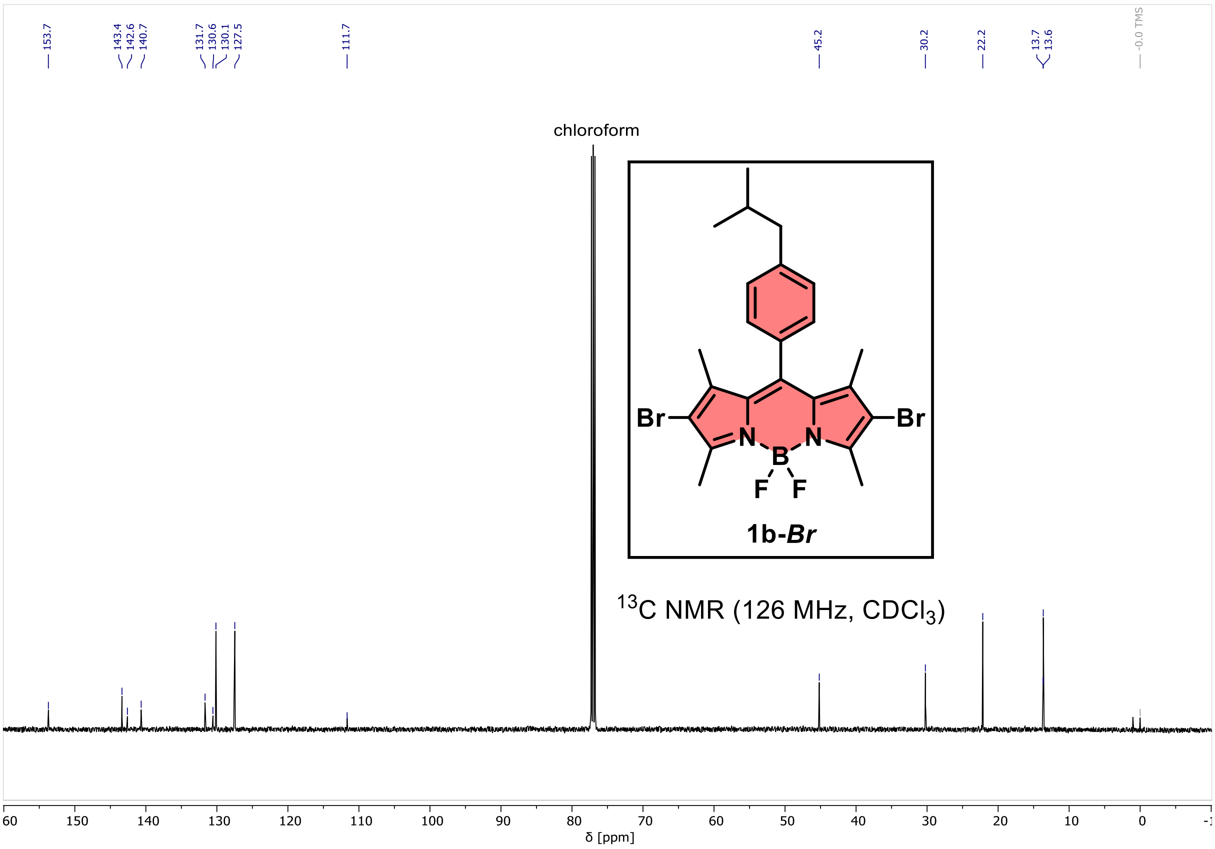

Figure S20: ^{13}C NMR spectrum (126 MHz) of **1b-*Br*** in $CDCl_3$ at room temperature.

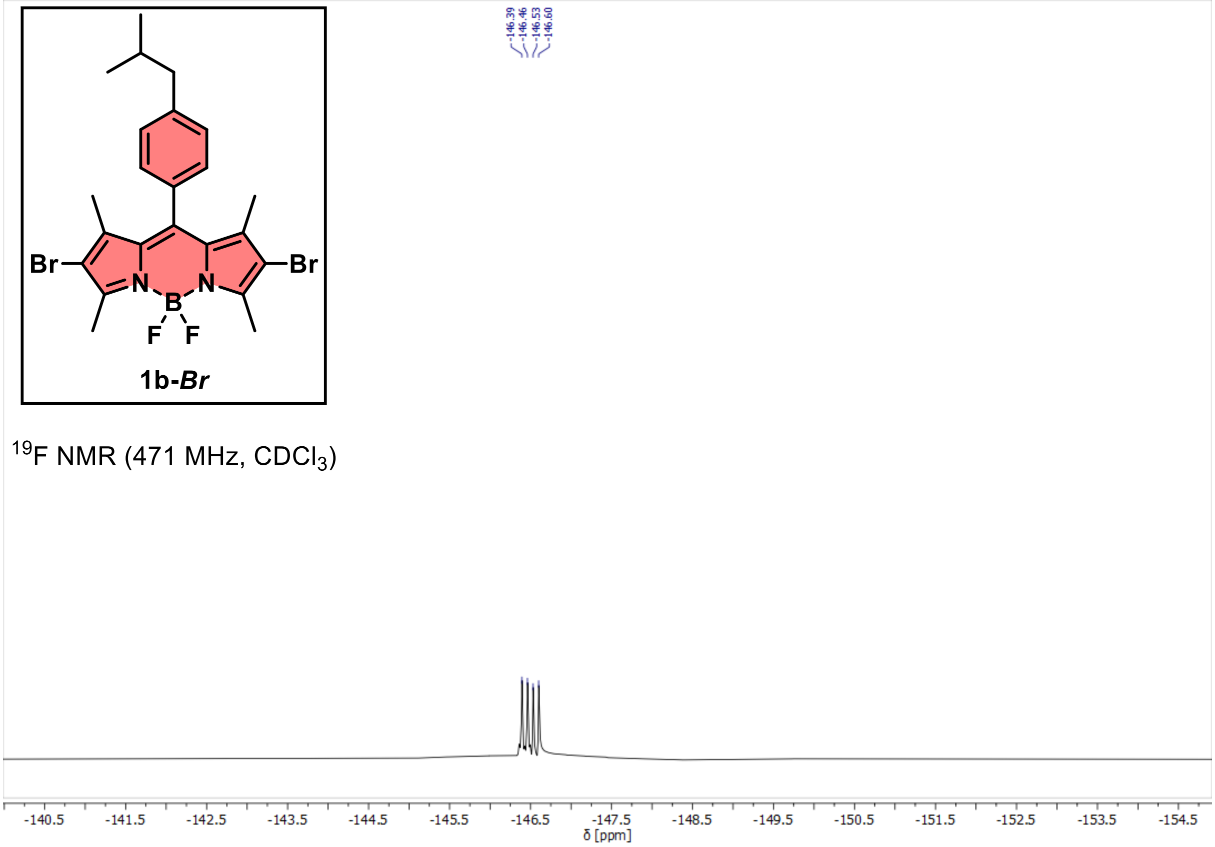

Figure S21: ^{19}F NMR spectrum (471 MHz) of **1b-*Br*** in $CDCl_3$ at room temperature.

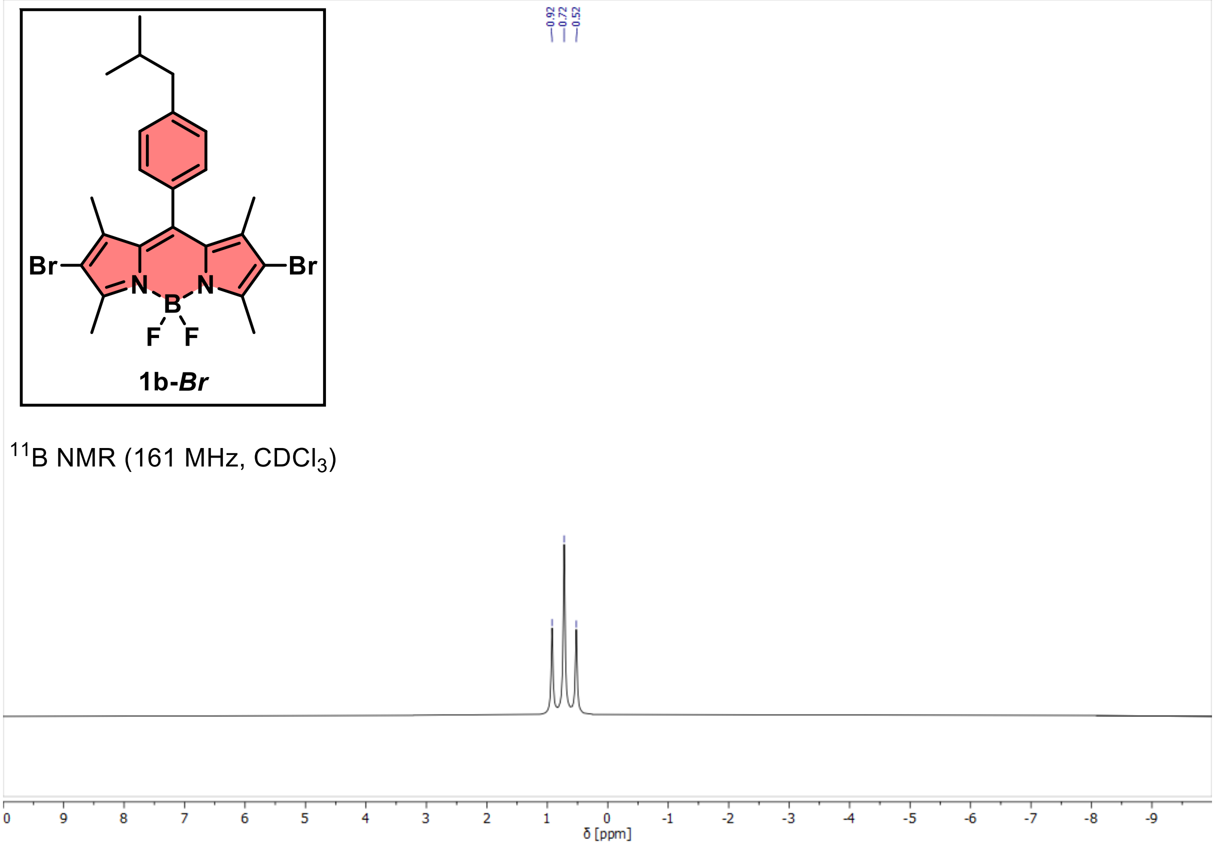

Figure S22: ^{11}B NMR spectrum (161 MHz) of **1b-*Br*** in $CDCl_3$ at room temperature.

4.2 Cyclized Products

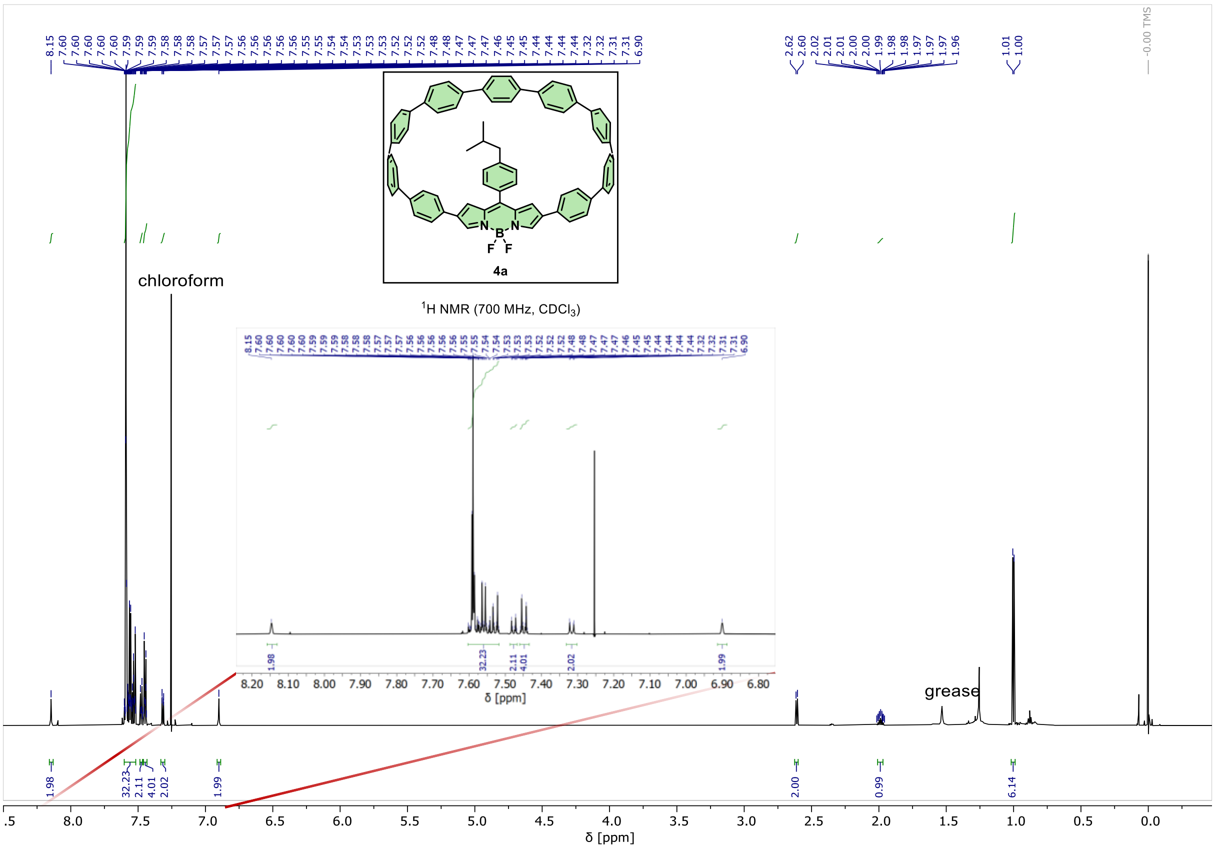

Figure S23: ^{1}H NMR spectrum (700 MHz) of **4a** in $CDCl_3$ at room temperature.

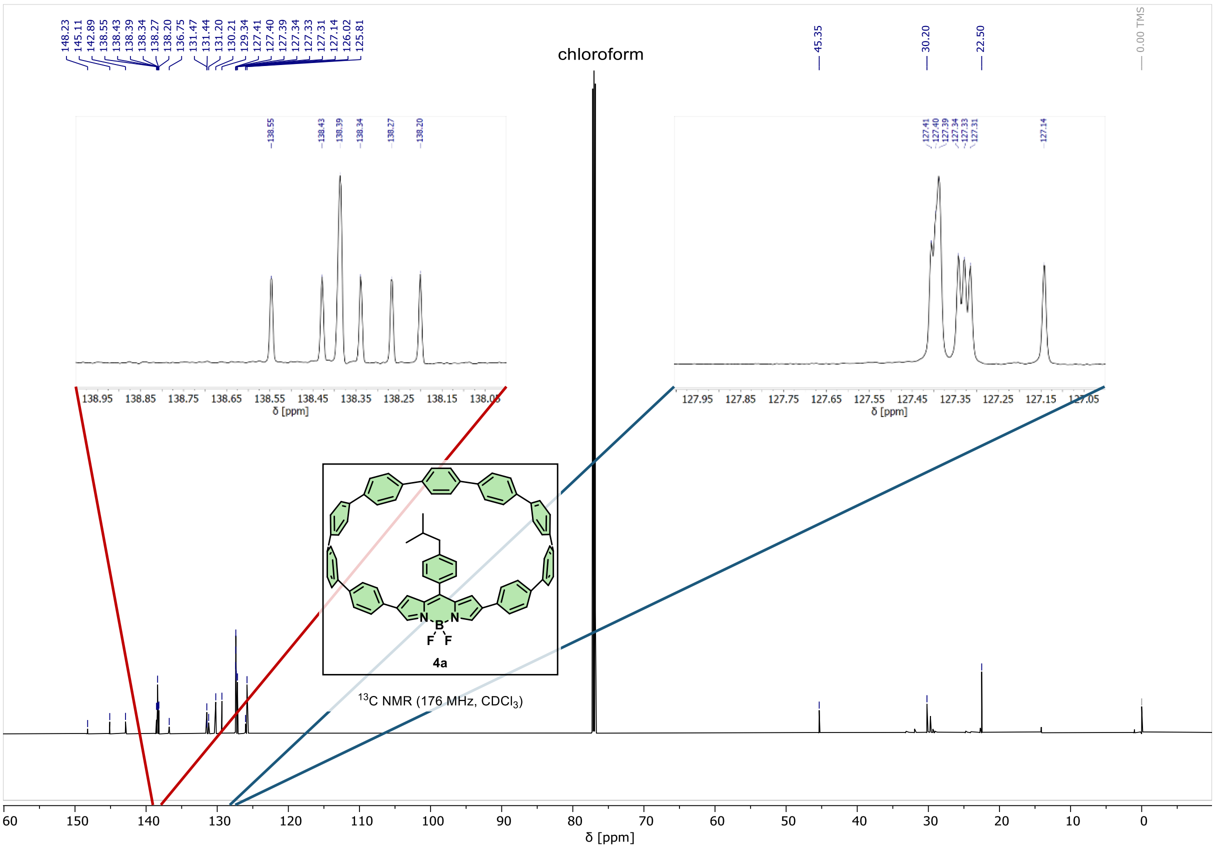

Figure S24: ^{13}C NMR spectrum (176 MHz) of **4a** in $CDCl_3$ at room temperature.

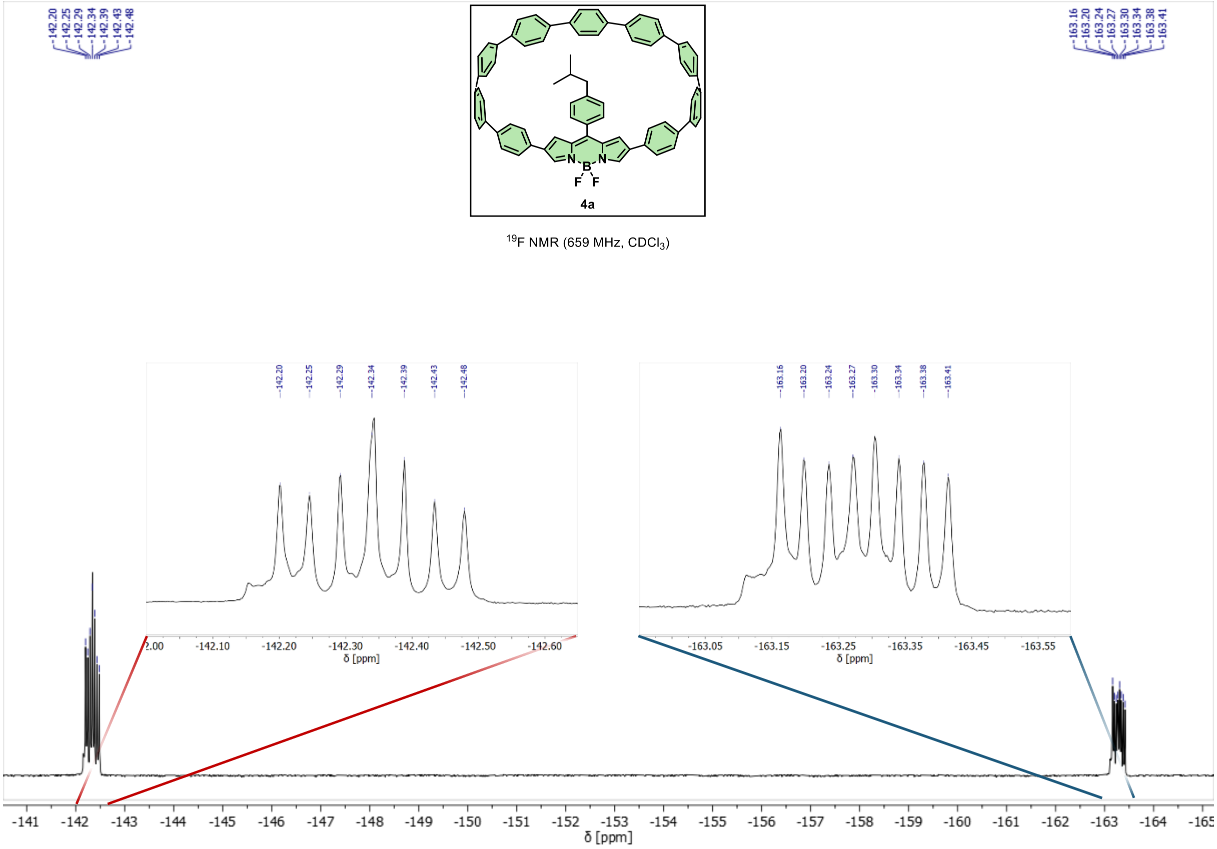

Figure S25: ^{19}F NMR spectrum (659 MHz) of **4a** in $CDCl_3$ at room temperature.

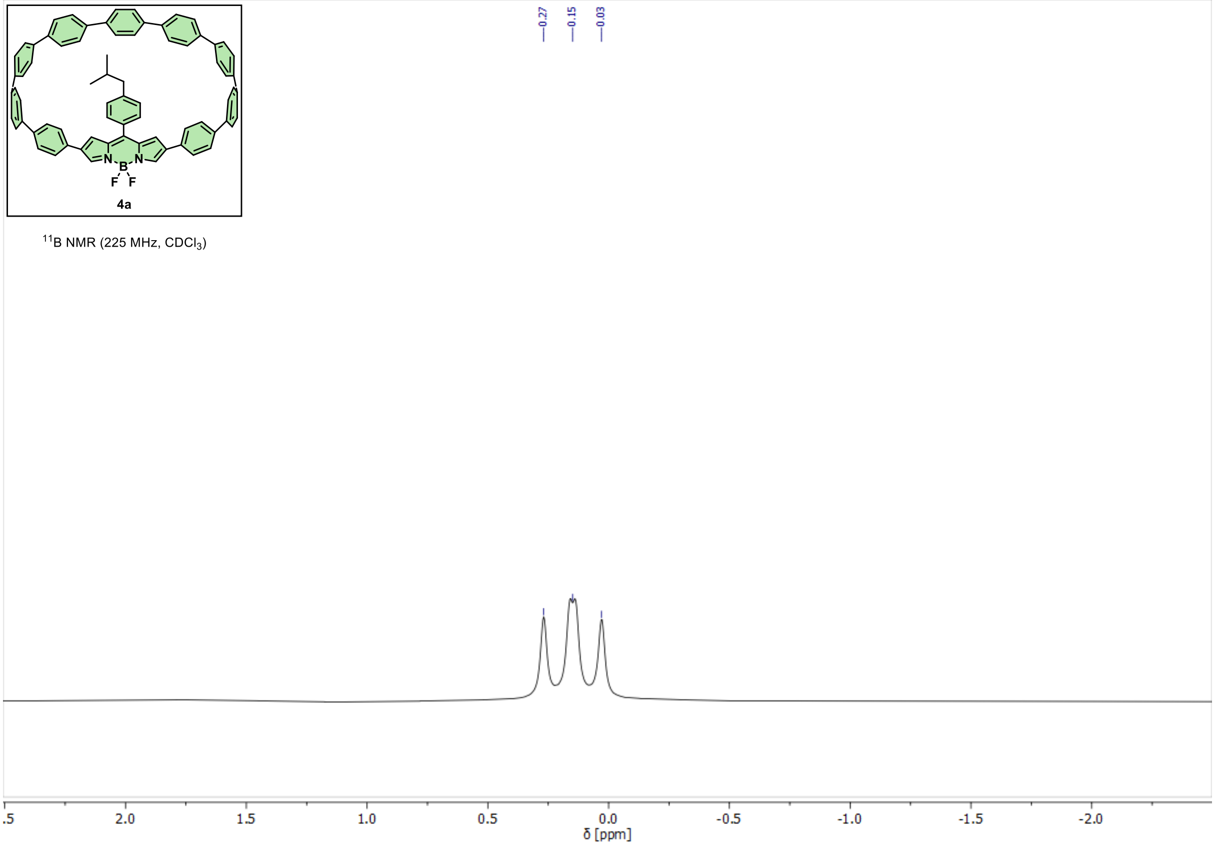

Figure S26: ^{11}F NMR spectrum (225 MHz) of **4a** in $CDCl_3$ at room temperature.

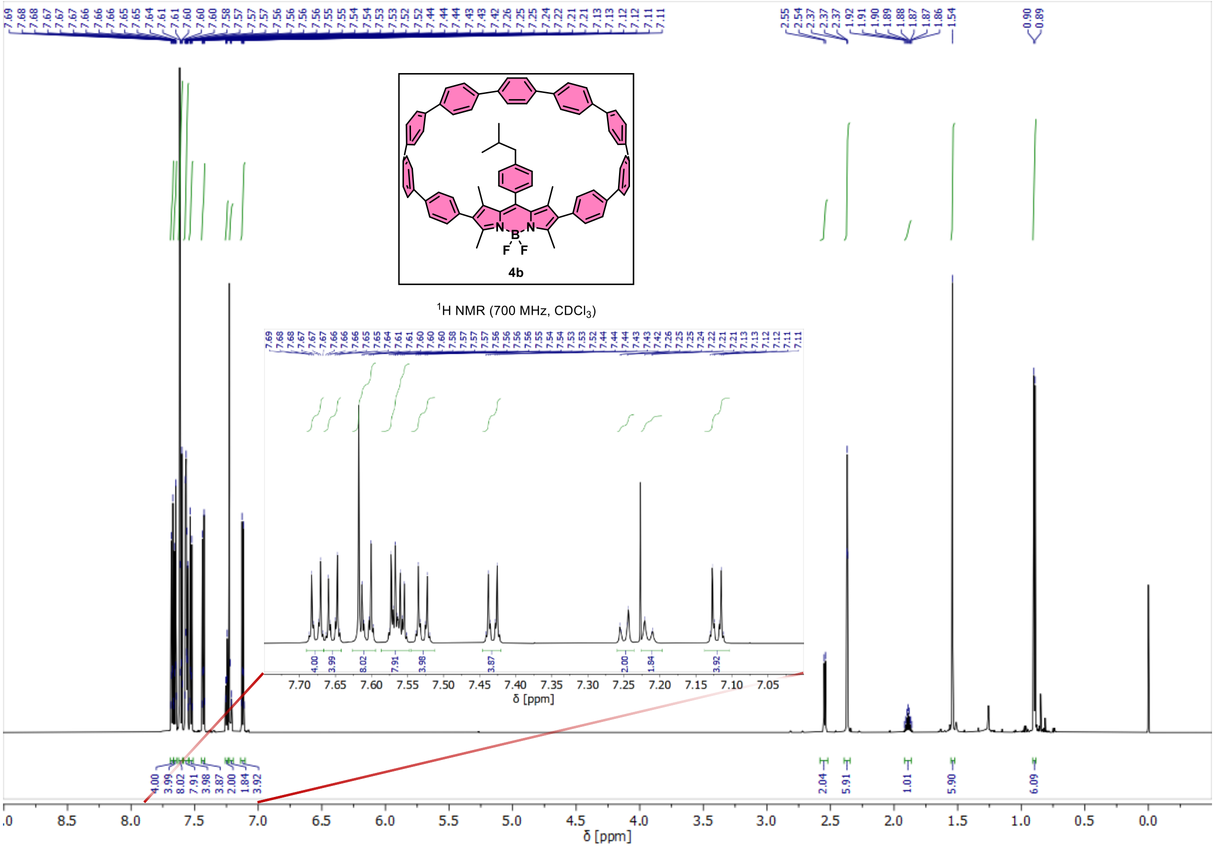

Figure S27: ^{1}H NMR spectrum (700 MHz) of **4b** in $CDCl_3$ at room temperature.

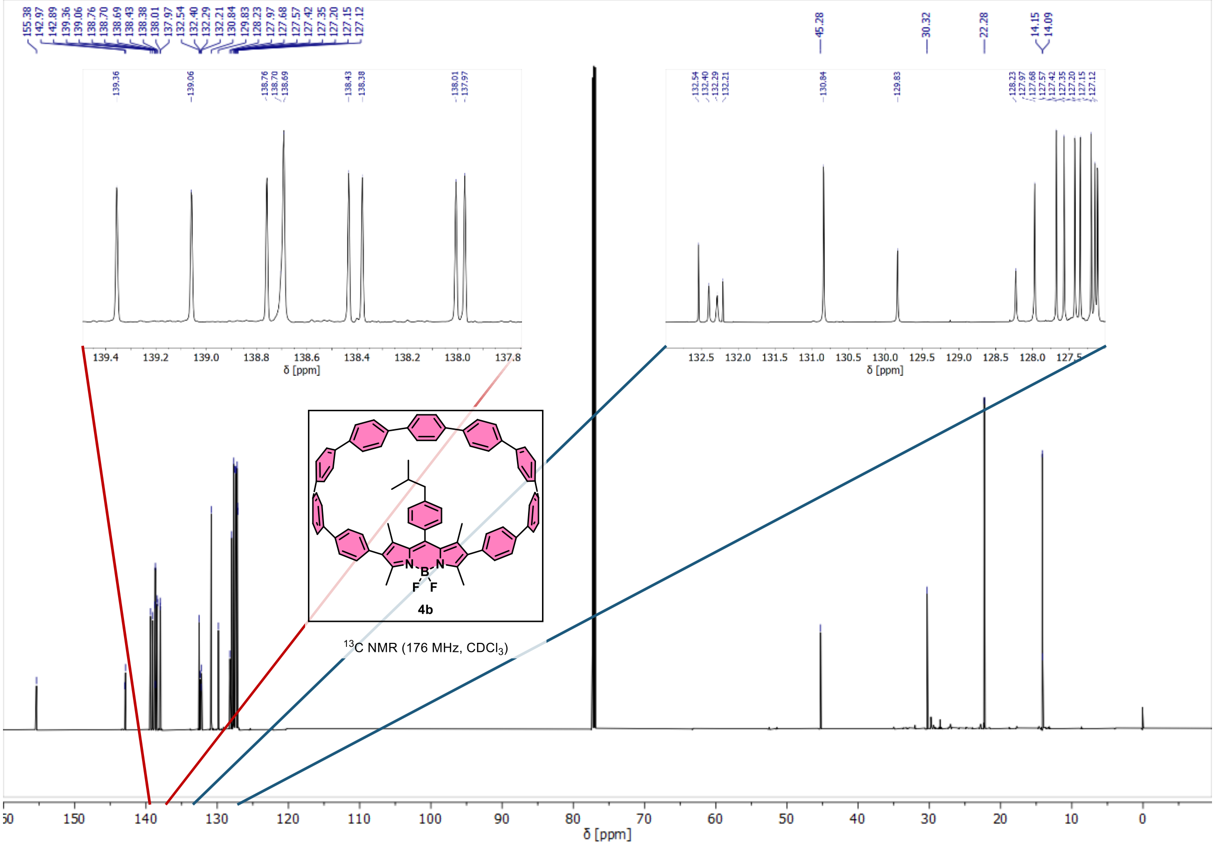

Figure S28: ^{13}C NMR spectrum (176 MHz) of **4b** in $CDCl_3$ at room temperature.

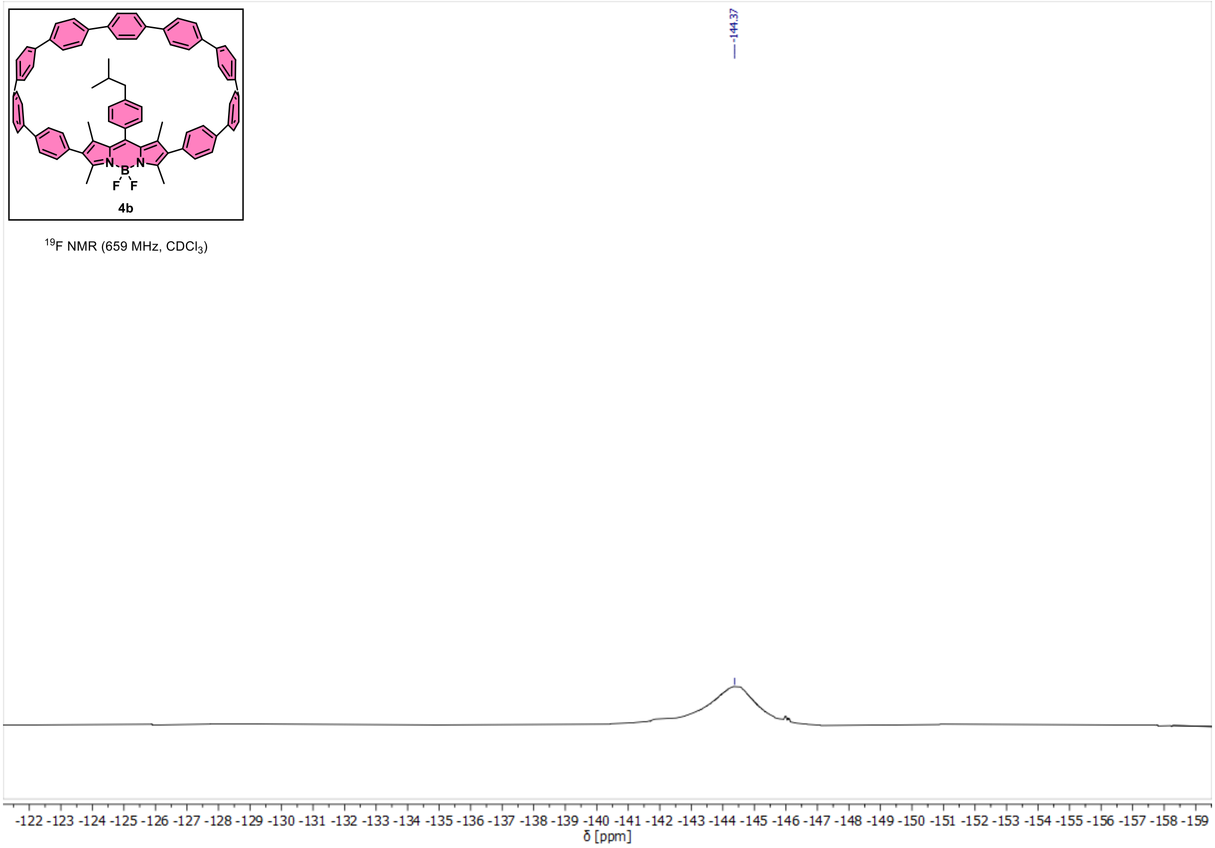

Figure S29: ^{19}F NMR spectrum (659 MHz) of **4b** in $CDCl_3$ at room temperature.

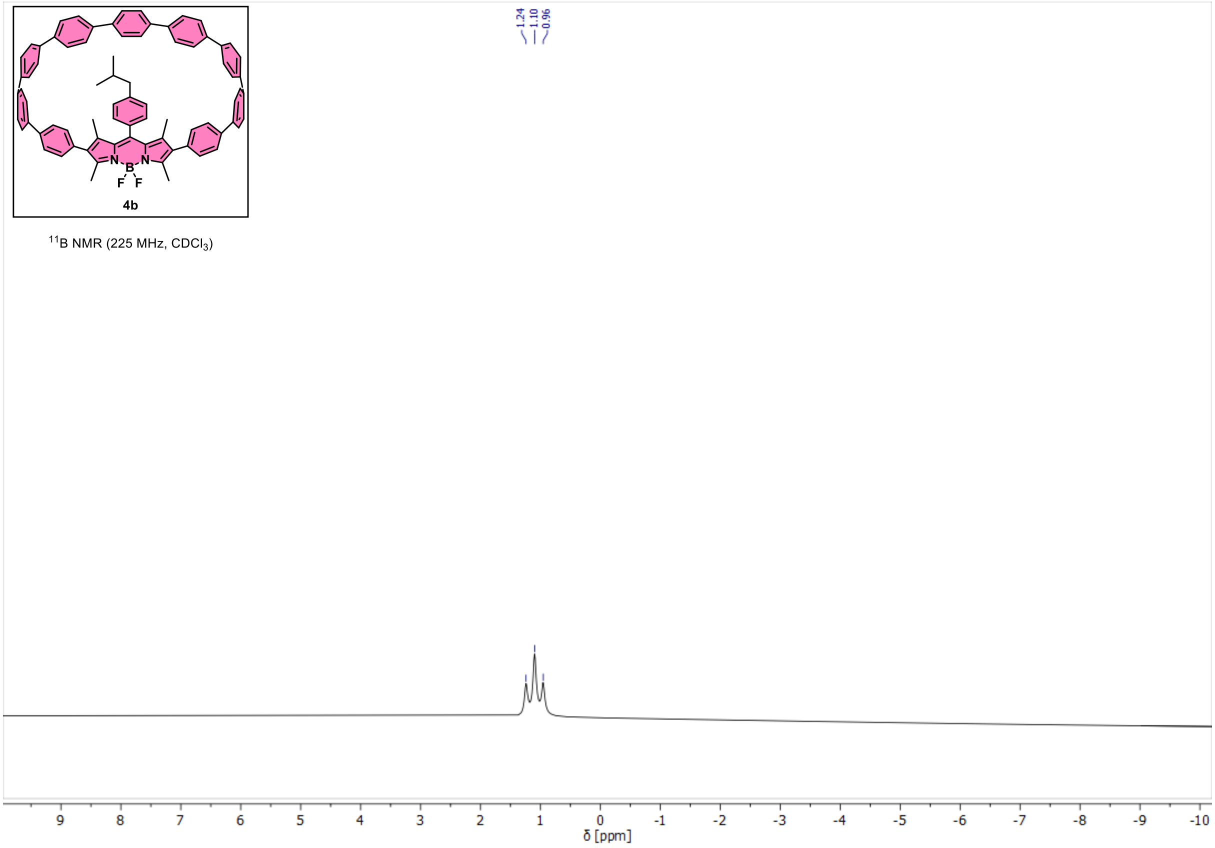

Figure S30: ^{11}B NMR spectrum (225 MHz) of **4b** in $CDCl_3$ at room temperature.

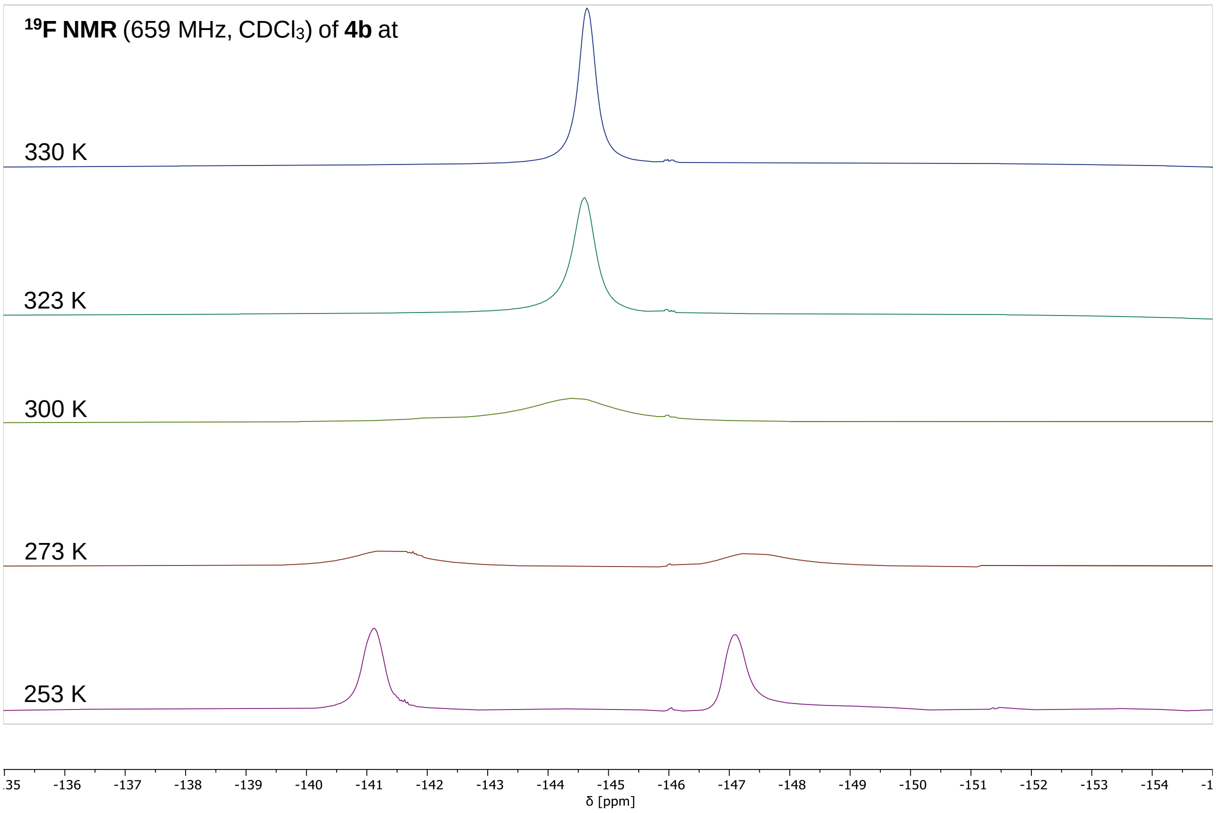

Figure S31: ^{19}F NMR spectrum (659 MHz) of **4b** in $CDCl_3$ at temperatures between 253 K and 330 K.

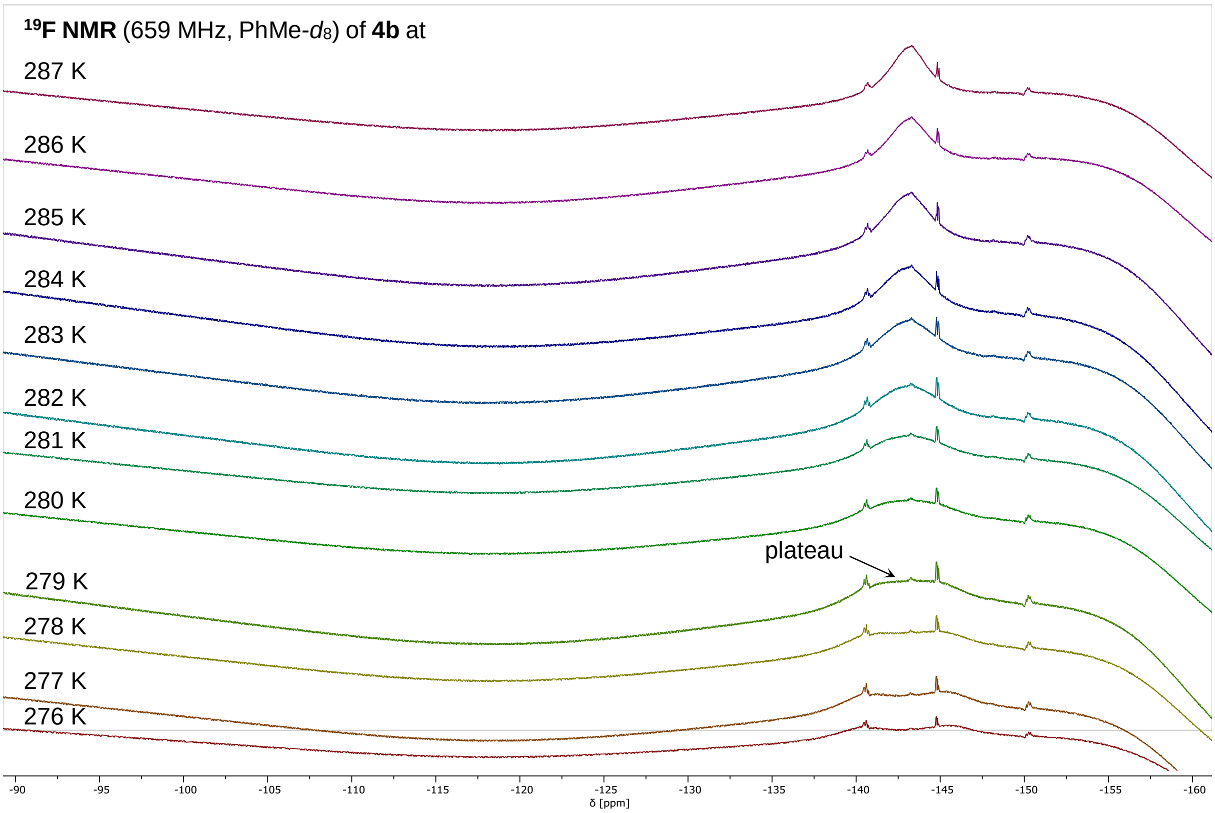

Figure S32: ^{19}F NMR spectrum (659 MHz) of **4b** in PhMe-d_8 at temperatures between 276 K and 287 K in steps of 1 K (coalescence at 280 K).

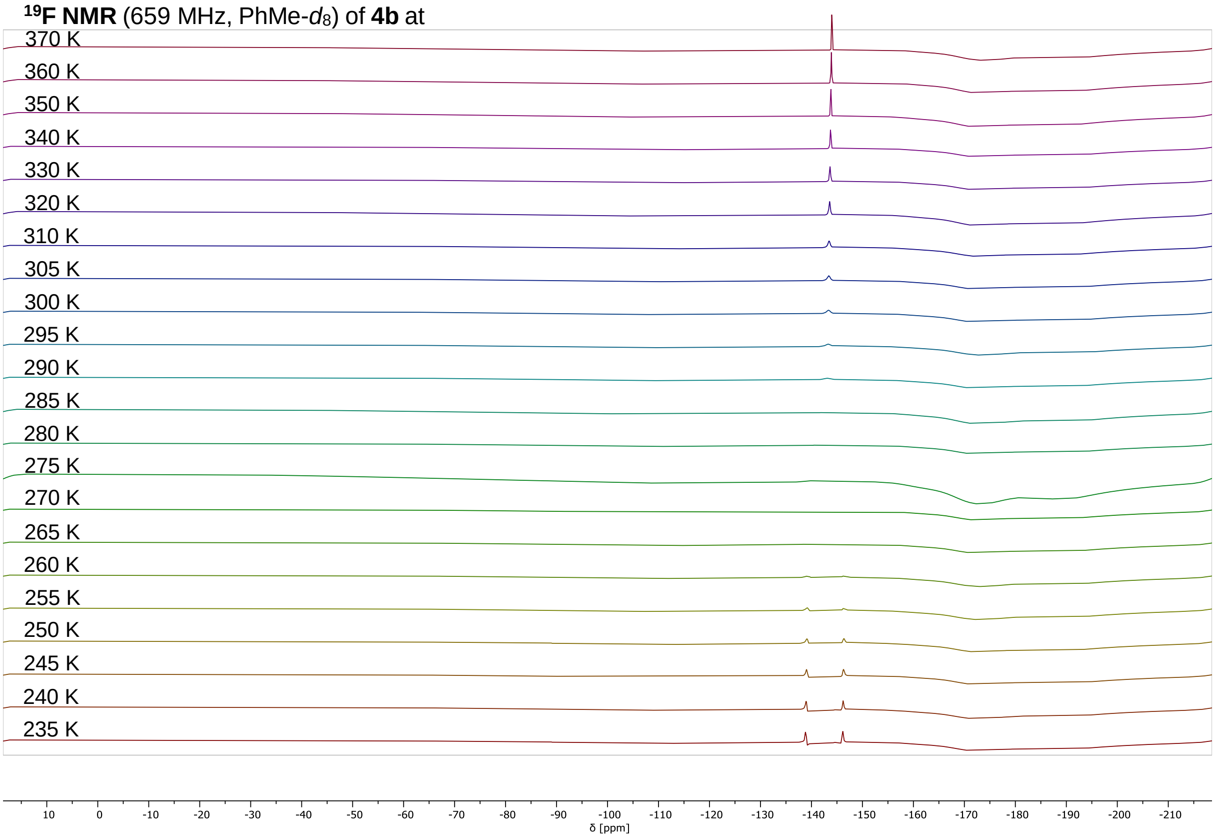

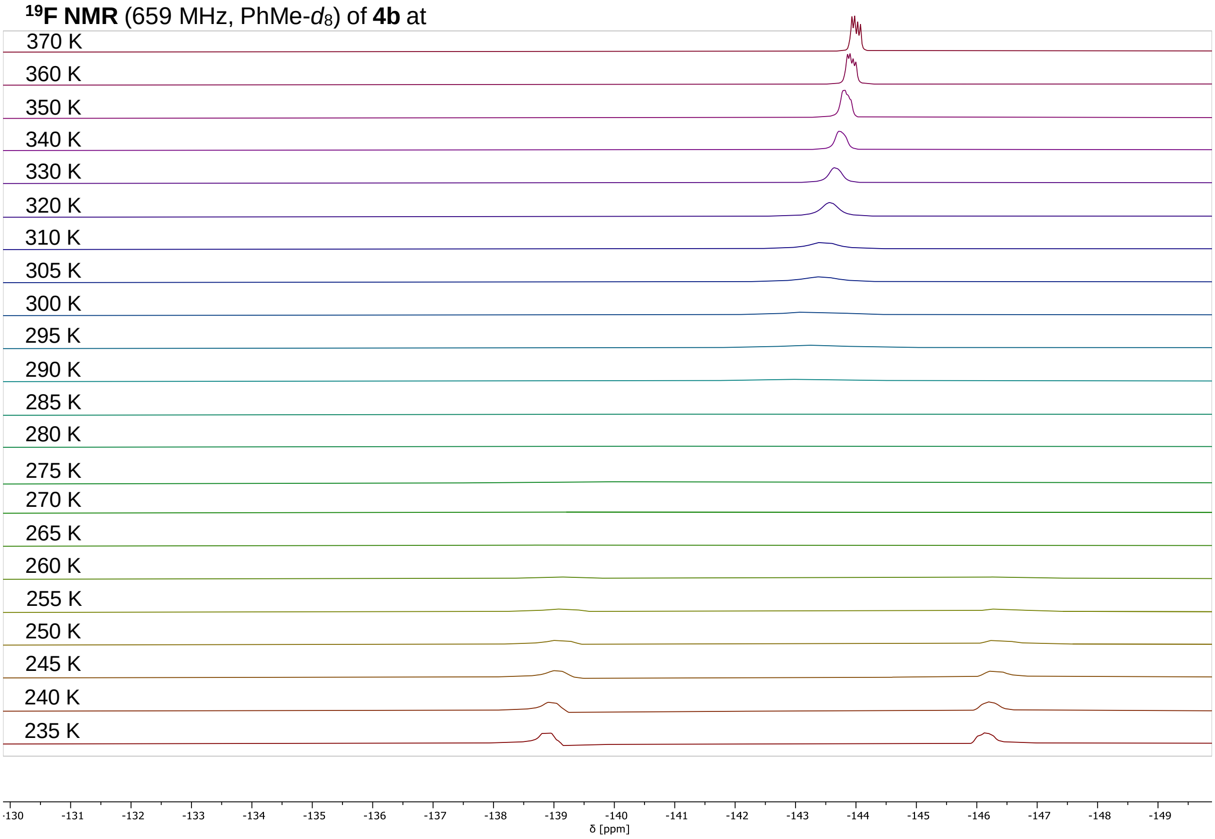

Figure S33: Full (top) and zoomed in (bottom) ^{19}F NMR spectrum (659 MHz) of **4b** in PhMe-d_8 at temperatures between 235 K and 370 K.

$$\Delta G^{\ddagger} = R\ T_c\ [22.96 + \ln(T_c / \Delta \nu)] \qquad \text{Eq. S1}^{[9]}$$

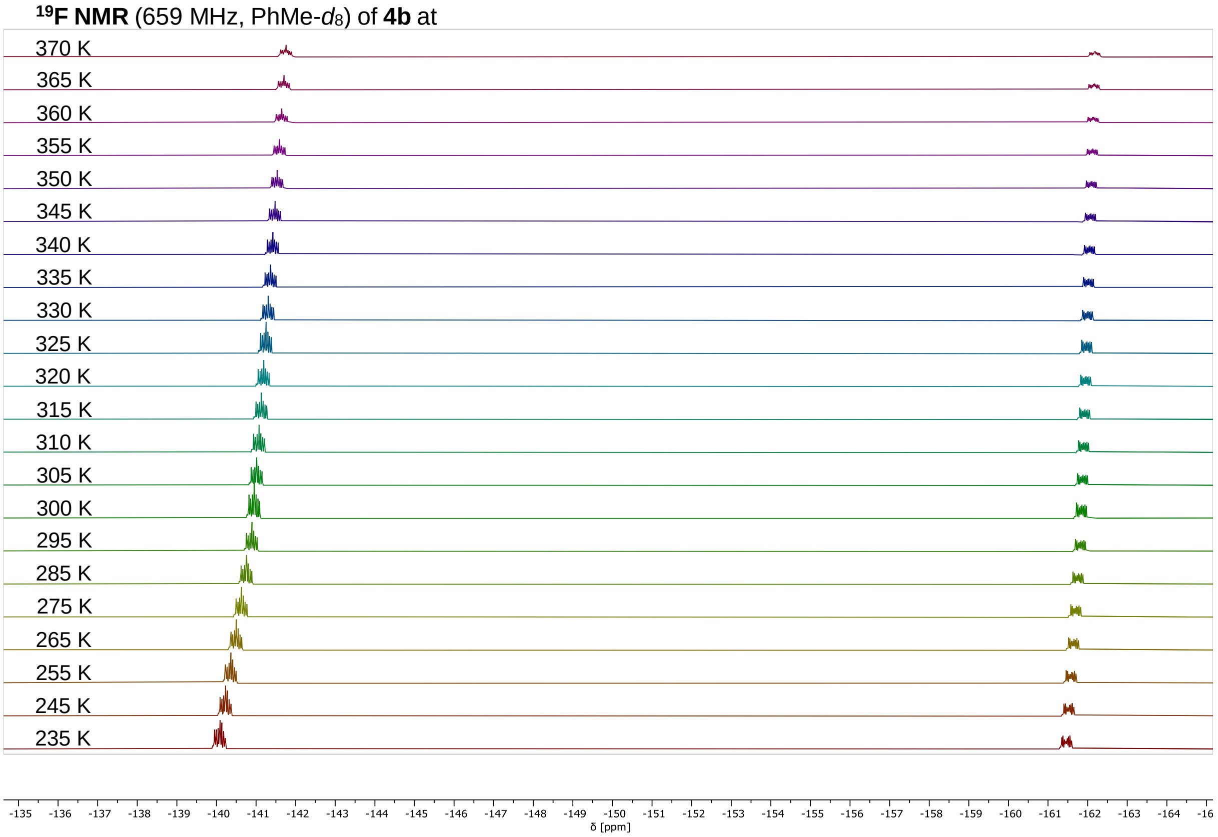

Figure S34: ^{19}F NMR spectrum (659 MHz) of **4a** in PhMe-d_8 at temperatures between 235 K and 370 K.

5 HR Mass Spectra

3a

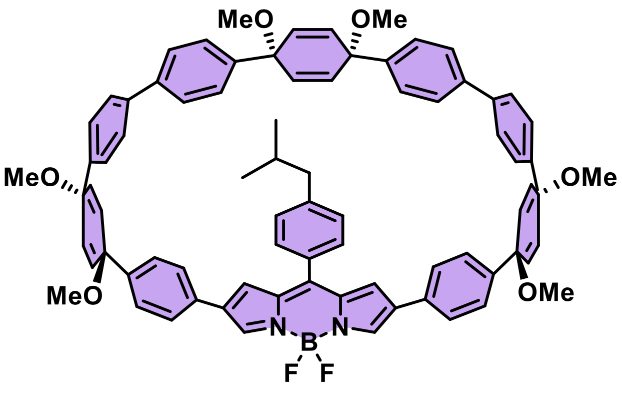

3a

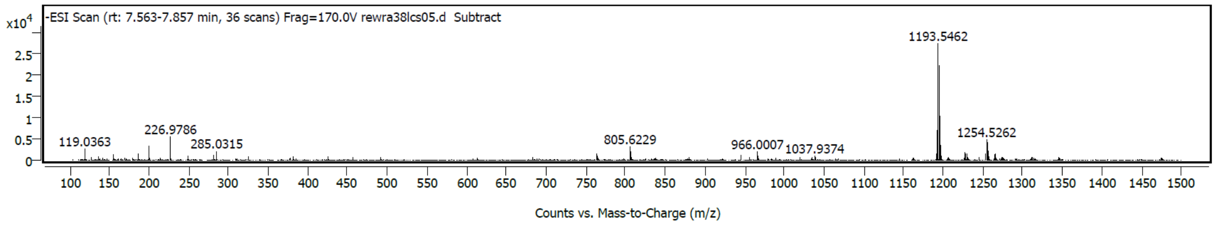

Figure S35: ESI (pos.) HR mass spectrum of **3a**.

3b

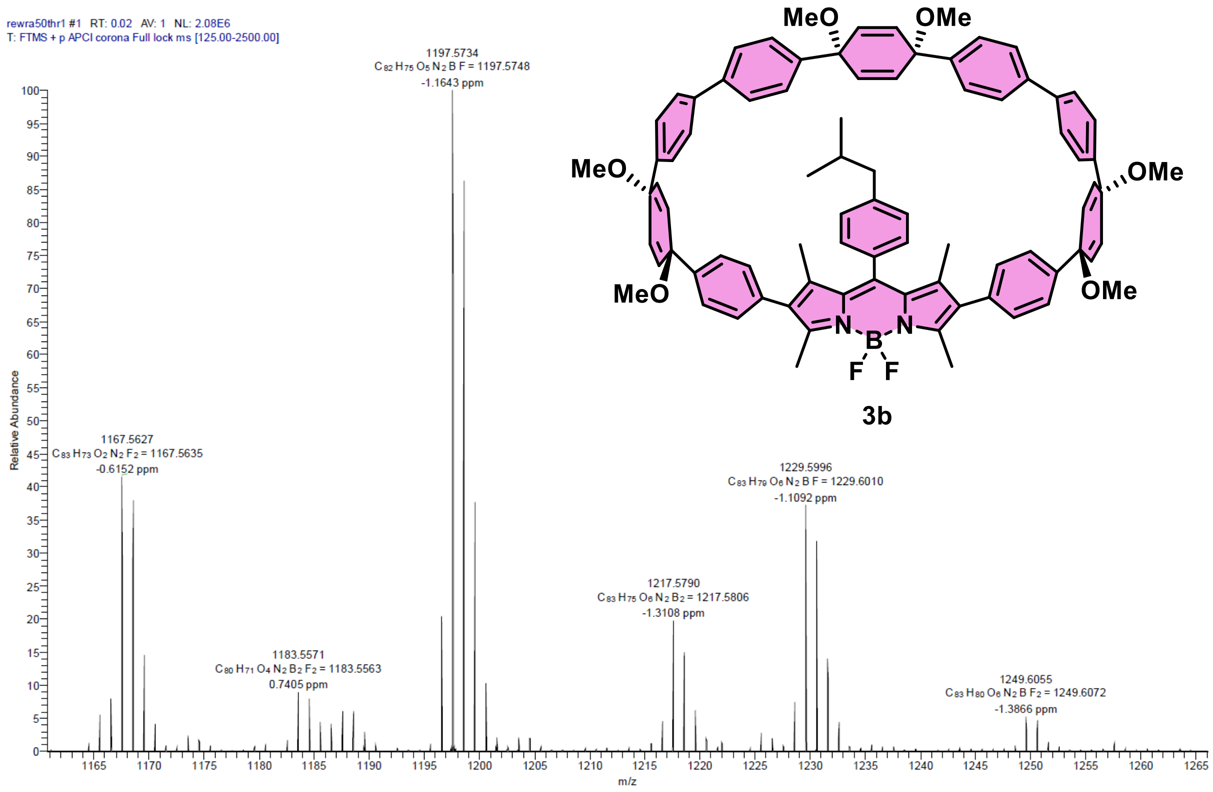

Figure S36: ESI (pos.) HR mass spectrum of **3b**.

4a

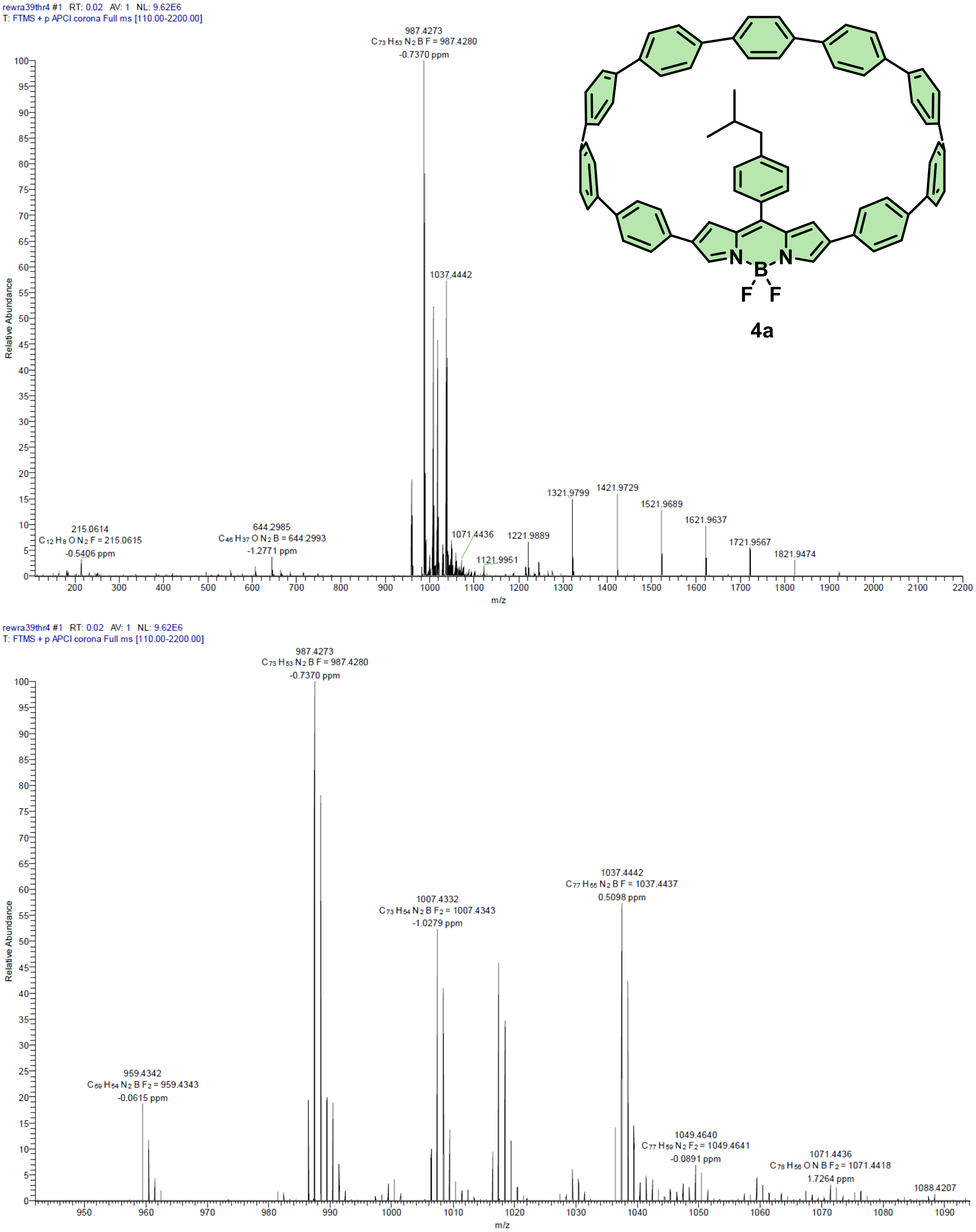

Figure S37: ESI (pos.) HR mass spectrum of **4a** (top: full; bottom: zoom with isotopic distribution pattern).

4b

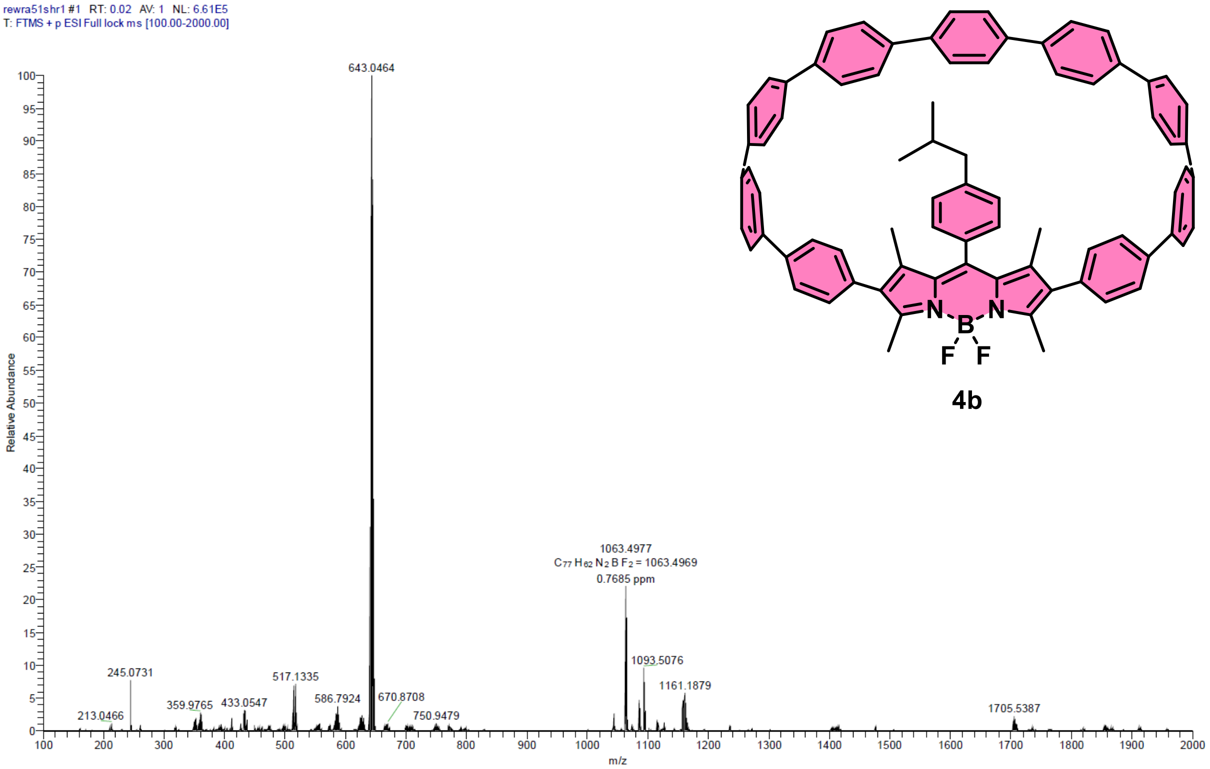

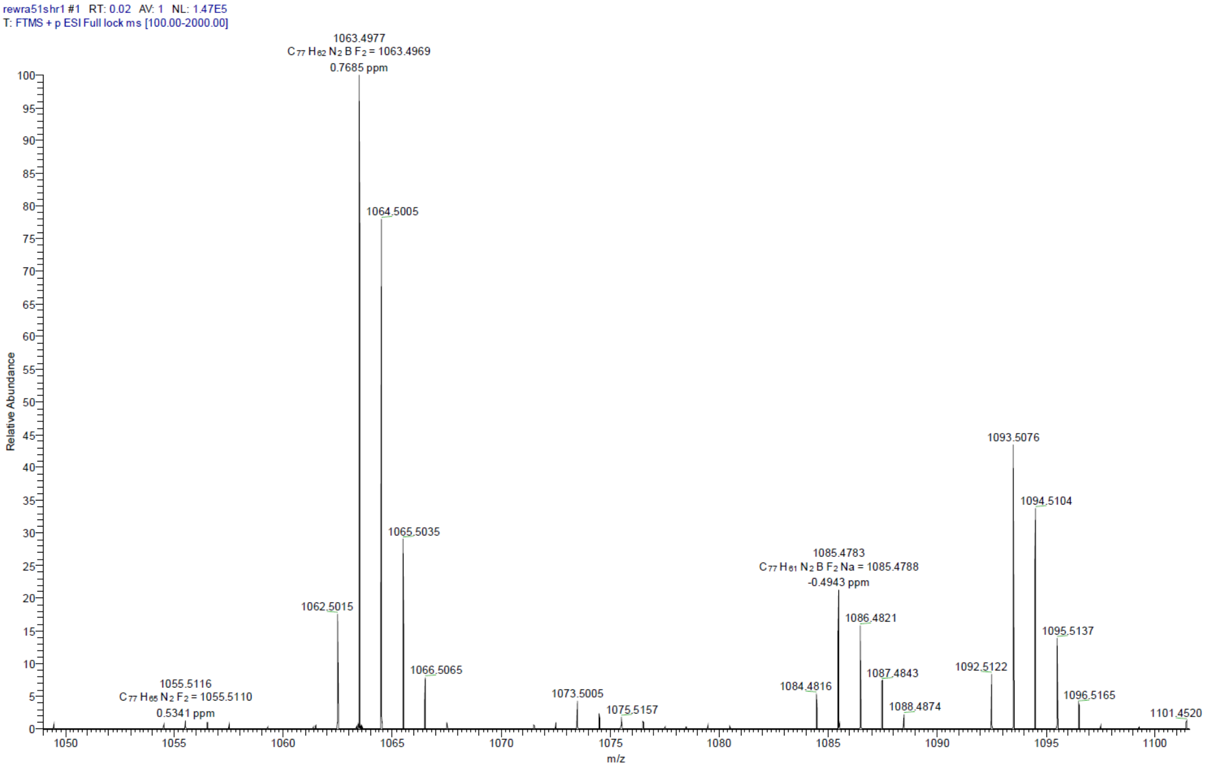

Figure S38: ESI (pos.) HR mass spectrum of **4b** (top: full; bottom: zoom with isotopic distribution pattern).

6 IR Spectra

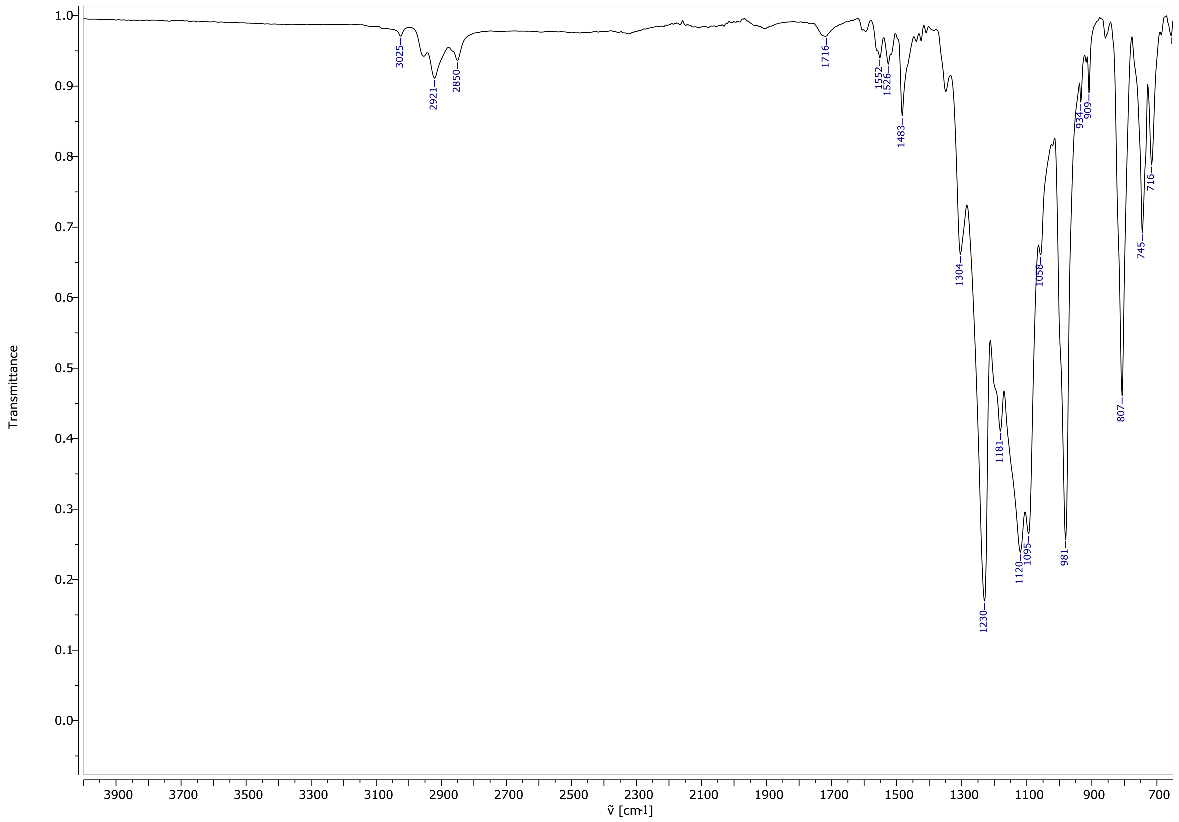

Figure S39: IR spectrum (Diamond ATR) of **4a**.

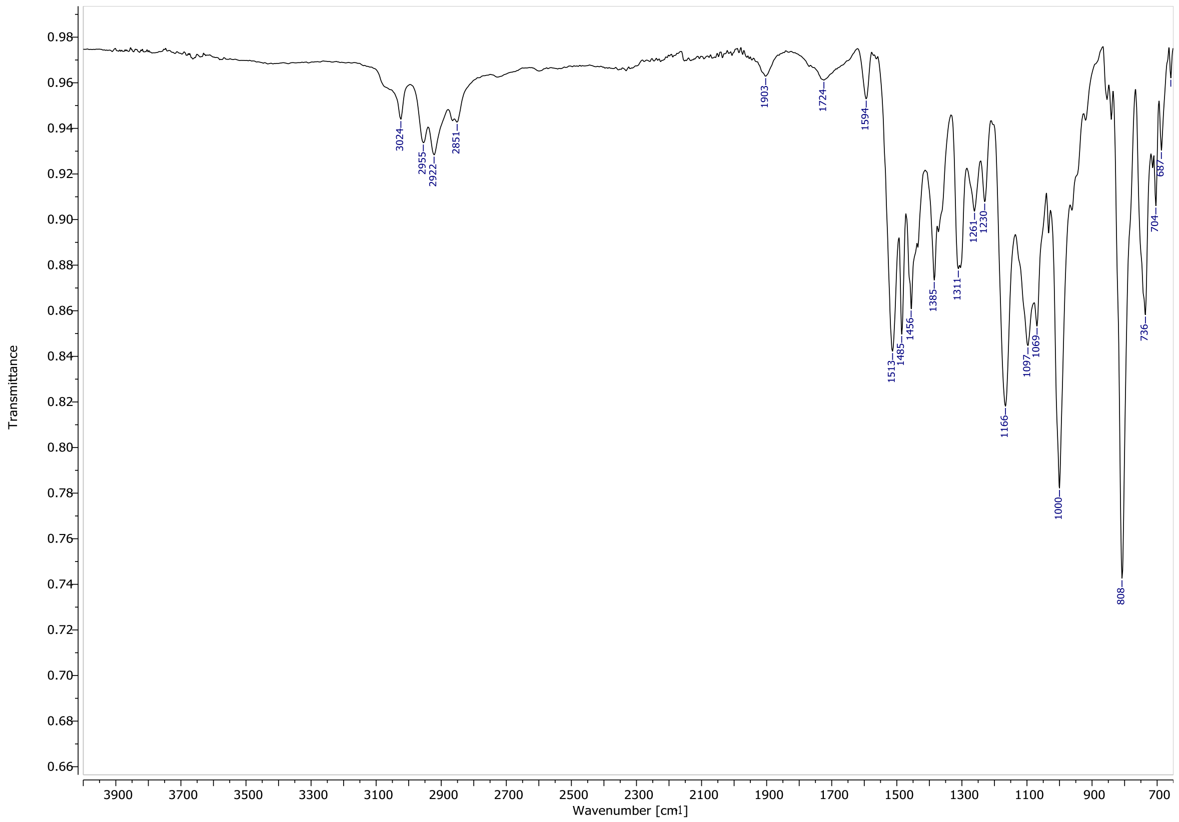

Figure S40: IR spectrum (Diamond ATR) of **4b**.

7 Photophysical Properties

7.1 Absorption and Emission Data

All absorption and emission measurements of new compounds were conducted at room temperature on the aforementioned instruments. For both, λ_{max} values were determined for solutions first in CyH, PhMe (in parenthesis), CH_2Cl_2 (*italicized*, all three spectroscopic grade from *Thermo Scientific*) and finally in degassed THF (**bold**, ROTISOLV®, UV/IR-grade from *Carl Roth*), since both MeOH and MeCN caused solubility issues.

Table S1: Absorption and emission data of prepared compounds.

Compound	λ^{A}_{max} [nm]	λ^{F}_{max} [nm]	$\Delta\tilde{\nu}$ [cm^{-1}]	ε [10^3 M^{-1} cm^{-1}]	Φ_F
**1a**	548<br>(550)<br>*545*<br>**545**	567<br>(574)<br>*568*<br>**570**	611<br>(760)<br>*743*<br>**805**	60<br>(67)<br>*56*<br>**51**	(0.08)<br>*0.07*
**1a-*H***	501<br>(503)<br>*500*<br>**499**	520<br>(525)<br>*521*<br>**523**	729<br>(833)<br>*806*<br>**920**	53<br>(52)<br>*52*<br>**59**	*0.13*
**1b**	(536)<br>*532*<br>**532**	(551)<br>*547*<br>**549**	(508)<br>515<br>**582**	(75)<br>*90*<br>**70**	(0.09)<br>*0.10*
**1b-*H***[4b]	(503)<br>*501*<br>**500**	(514)<br>*511*<br>**510**	(426)<br>*391*<br>**392**	(87)<br>*89*<br>**91**	*0.51*
**4a**	332, 647<br>(338, 653)<br>*335*, *641*<br>**337**, **644**	695<br>(706)<br>*710*<br>**706**	1067<br>(1150)<br>*1516*<br>**1364**	59, 15<br>(57, 15)<br>*76*, *18*<br>**76**, **15**	(< 0.01)<br>< *0.01*
**4b**	332, 564<br>(337, 569)<br>*335*, *564*<br>**335**, **563**	609<br>(615)<br>*613*<br>**612**	1310<br>(1315)<br>1417<br>**1422**	n.d.[a]<br>(61, 34)<br>*73*, *37*<br>**63**, **32**	(0.02)<br>*0.02*

[a] Not detected/determined (due to poor solubility; maximum is normalized to CH_2Cl_2).

7.2 Absorption and Emission Spectra

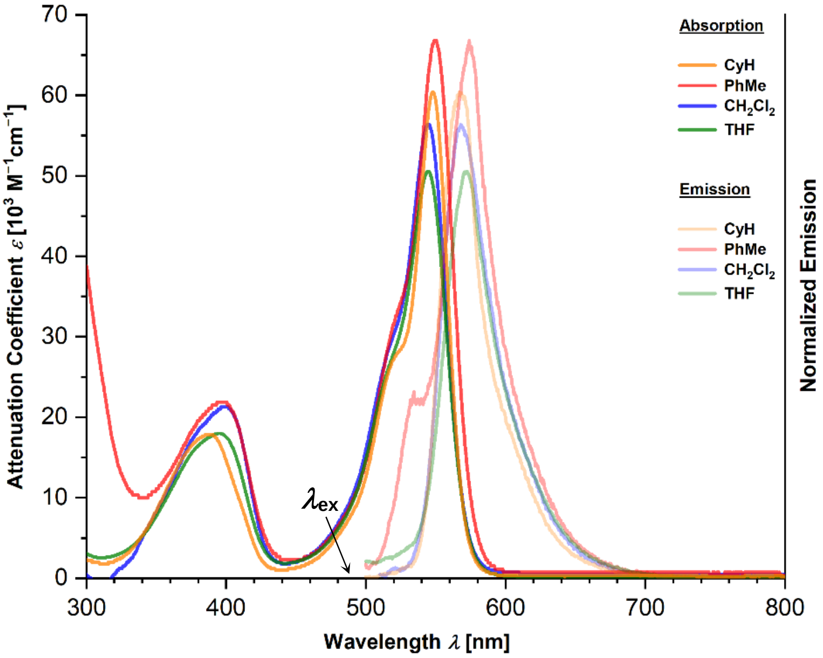

Figure S41: Absorption and emission spectra of compound **1a** at room temperature in CyH, PhMe, CH_2Cl_2 and THF. Emission is normalized (λ_{ex} = 490 nm).

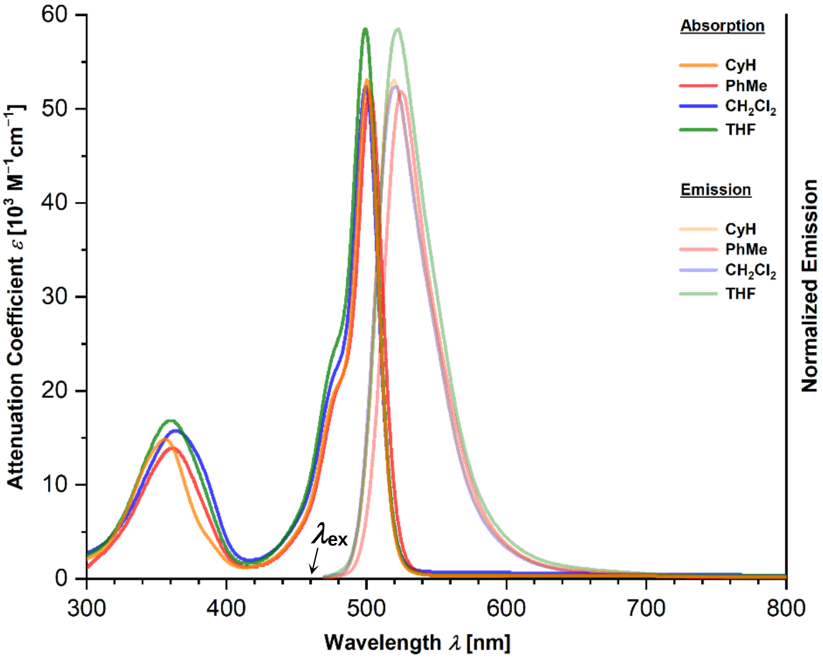

Figure S42: Absorption and emission spectra of compound **1a-*H*** at room temperature in CyH, PhMe, CH_2Cl_2 and THF. Emission is normalized (λ_{ex} = 460 nm).

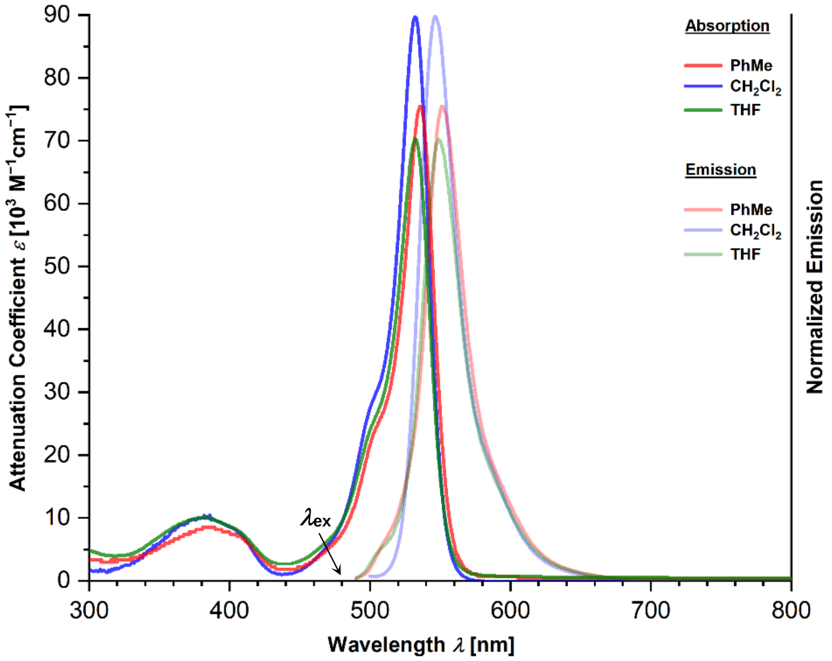

Figure S43: Absorption and emission spectra of compound **1b** at room temperature in PhMe, CH_2Cl_2 and THF. Emission is normalized (λ_{ex} = 480 nm).

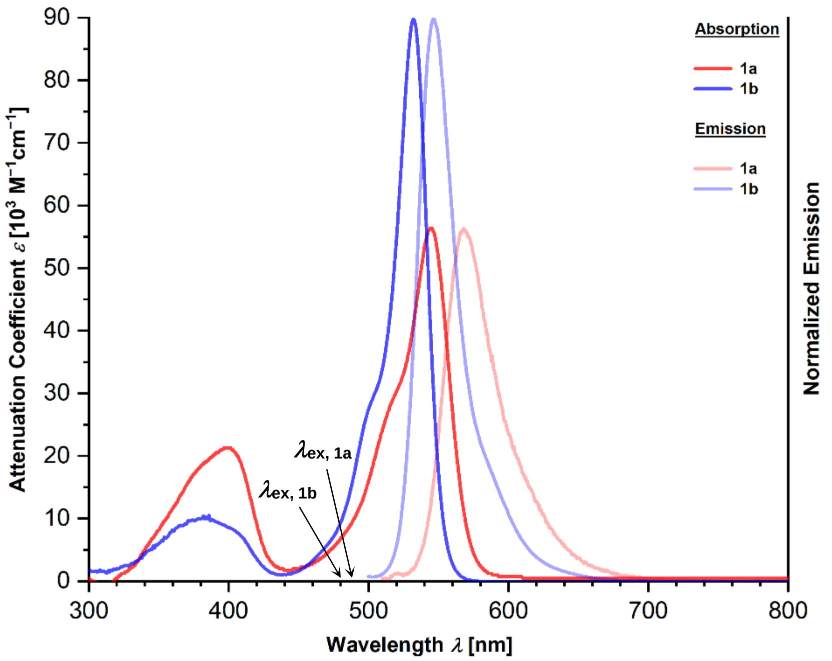

Figure S44: Absorption and emission (normalized) spectra of compounds **1a** and **1b** at room temperature in CH_2Cl_2.

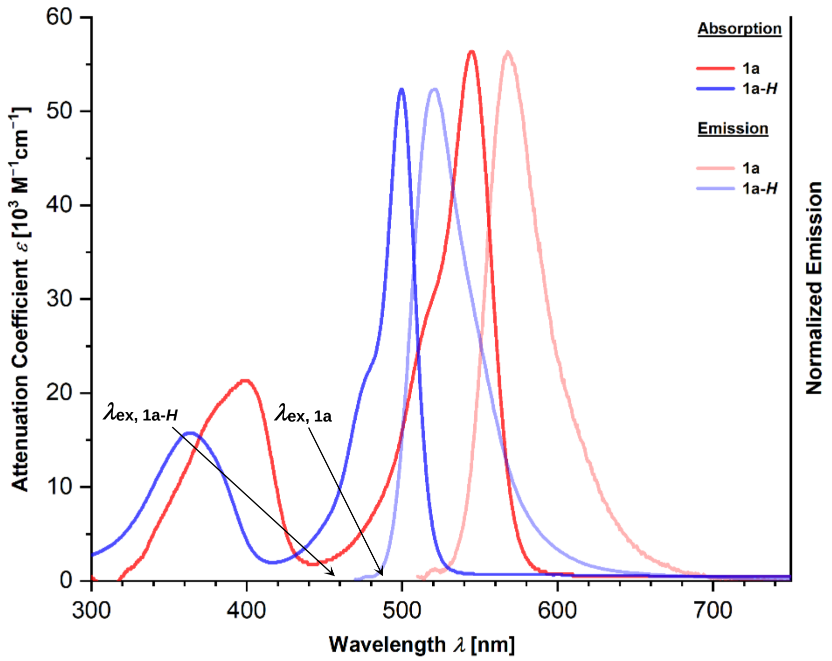

Figure S45: Absorption and emission (normalized) spectra of compounds **1a** and **1a-*H*** at room temperature in CH_2Cl_2.

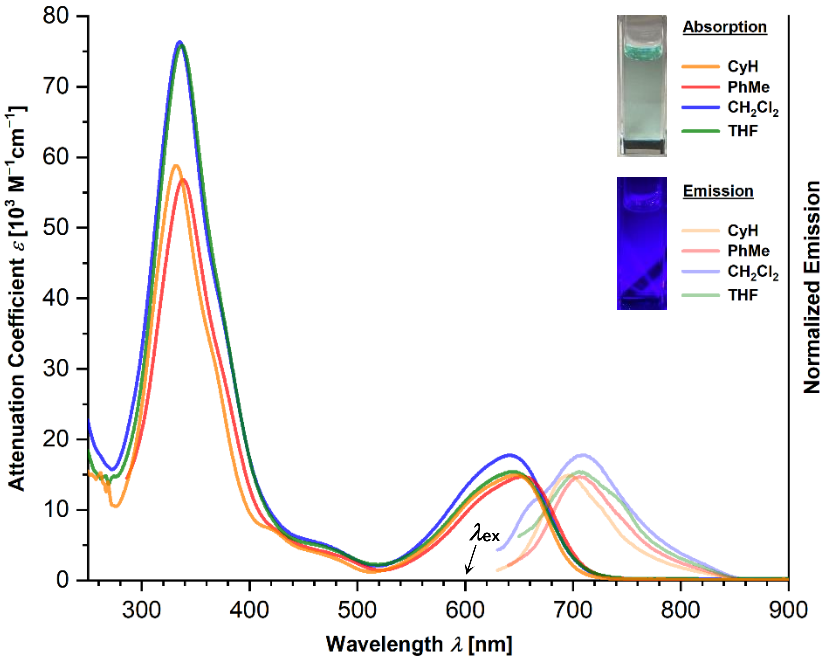

Figure S46: Absorption and emission spectra of compound **4a** at room temperature in CyH, CH_2Cl_2, PhMe and THF. Emission is normalized (λ_{ex} = 600 nm).

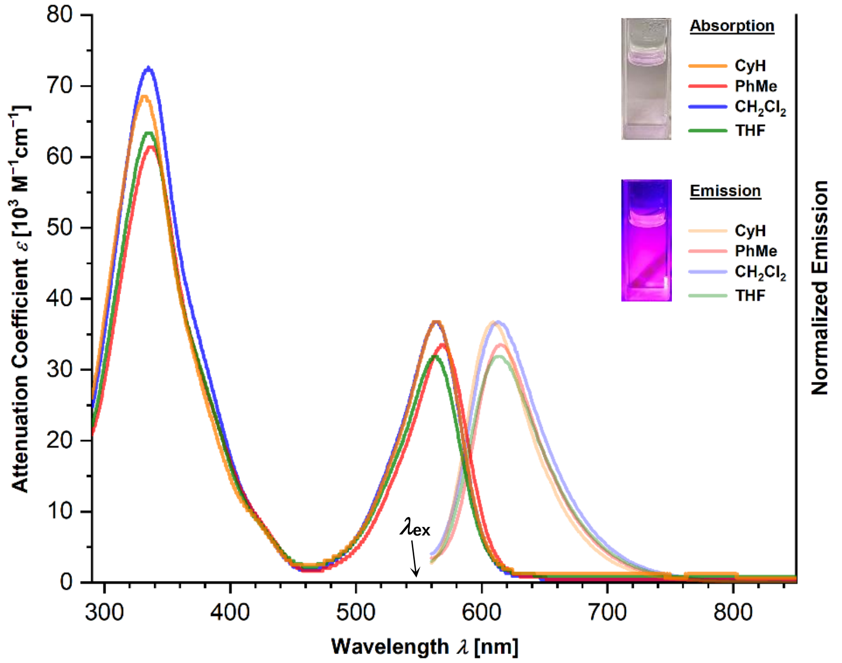

Figure S47: Absorption and emission spectra of compound **4b** at room temperature in CyH, CH_2Cl_2, PhMe and THF. Emission is normalized (λ_{ex} = 550 nm).

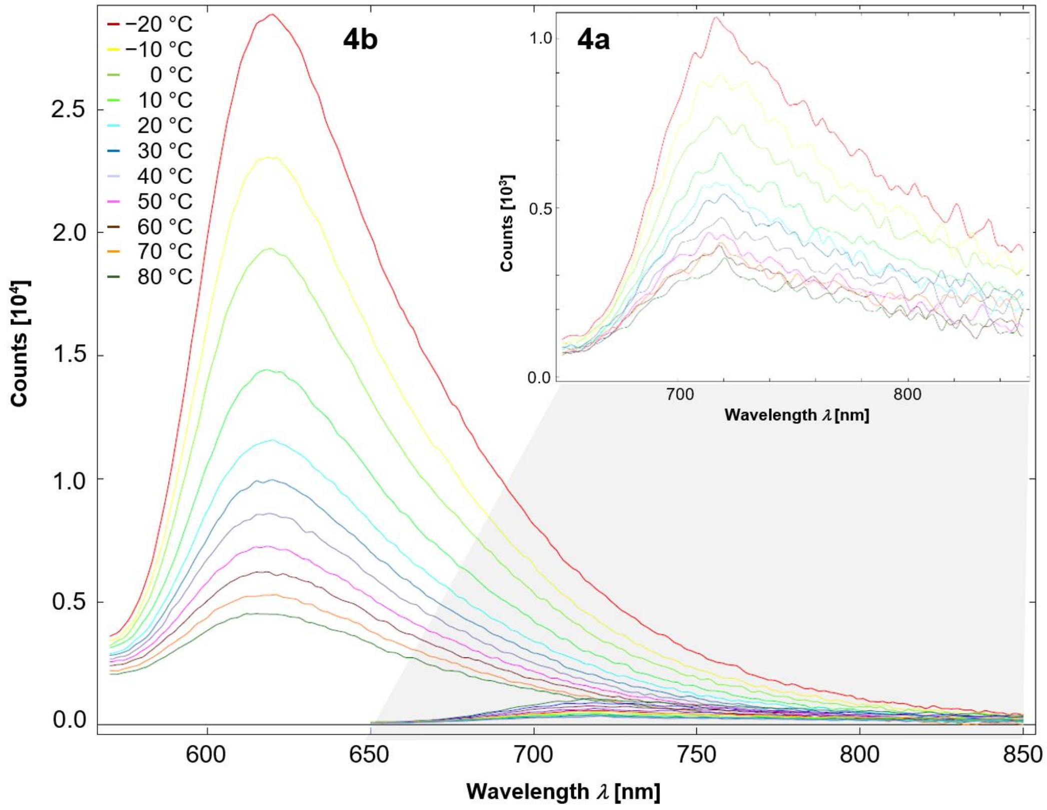

Figure S48: Emission spectra of compounds **4a** (λ_{ex} = 600 nm) and **4b** (λ_{ex} = 550 nm) at temperatures from −20 °C to 80 C (in steps of 10 °C) in PhMe.

8 Crystal Structure Determinations

Crystals were mounted in inert oil on Hampton loops and transferred to the cold gas stream of a Rigaku/OD XtaLAB Synergy diffractometer. Mirror-focussed Mo-$K\alpha$ radiation was employed for the intensity measurements. Absorption corrections were implemented on the basis of multi-scans. All compounds crystallized solvent-free by slow evaporation of dichloromethane at room temperature. Compounds **1b** and **1b-*Br*** are isotypic. The structures were refined anisotropically on F^2 using the program SHELXL-2018.[10] Hydrogen atoms were included using rigid methyl groups or a riding model starting from calculated positions.

Exceptions/special details:

The *iso*-butylphenyl substituent has often proved difficult to refine, as it has a high tendency to disorder. This was the case for all three structures **1b**, **1b-*Br*** and **1b-*H***. In all cases, appropriate restraints/constraints were employed to improve refinement stability, but the dimensions of disordered groups should always be interpreted with caution.

Compound **1b**. The *iso*-butylphenyl substituent (C14 – C23) is disordered over two sites with a common atom C14. The relative occupancies refined to 0.646(4) and 0.354(4). The less-occupied site (atom names with primes) was refined isotropically. The geometry of the less-occupied component at the common atom C14 is not entirely satisfactory, and this atom may also be slightly disordered.

Compound **1b-*Br***. Atoms C15 – C23 are disordered over two sets of positions with occupancies 0.794(4) and 0.206(4). The less-occupied site (atom names with primes) was refined isotropically. Because some of the atoms sites were close to each other, the refinement had a marked tendency to instability. The disorder is shown explicitly in Figure S51.

Compound **1b-*H***. The asymmetric unit contains two independent molecules. One of these is disordered; the atoms C14 – C23 occupy two alternative sets of sites with occupancies 0.534(2) and 0.466(2). Again, the alternative positions of the ring atoms C14 – C19 are very close to each other.

Crystallographic data are summarized in Table S2. Additionally, complete data have been deposited with the Cambridge Crystallographic Data Centre under the numbers CCDC 2424356 – 2424358. Copies of the data can be obtained free of charge from www.ccdc.cam.ac.uk/data_request/cif.

Table S2: Crystallographic data and structure refinement details.

Compound	1b	1b-*Br*	1b-*H*
CCDC number	2424356	2424357	2424358
Formula	$C_{23}H_{25}BF_2I_2N_2$	$C_{23}H_{25}BBr_2F_2N_2$	$C_{23}H_{27}BF_2N_2$
M_r	632.06	538.08	380.27
Cryst. size (mm)	0.17 x 0.17 x 0.03	0.22 x 0.18 x 0.15	0.22 x 0.15 x 0.08
Crystal system	monoclinic	monoclinic	monoclinic
Space group	$P2_1/n$	$P2_1/n$	$P2_1/c$
Temperature (°C)	−173	−173	−173
a (Å)	9.13803(12)	9.0351(2)	21.3594(5)
b (Å)	10.83038(14)	10.4791(3)	11.0993(2)
c (Å)	23.5435(3)	23.3307(6)	18.1814(4)
α (°)	90	90	90
β (°)	95.9596(12)	94.374(2)	107.336(2)
γ (°)	90	90	90
V (Å^3)	2317.47	2202.50	4114.56
Z	4	4	8
D_x (Mg m^{-3})	1.812	1.623	1.228
λ (Å)	0.71073	0.71073	0.71073
μ (mm^{-1})	2.7	3.7	0.08
Transmissions	0.878 – 1.000	0.701 – 1.000	0.887 – 1.000
F(000)	1224	1080	1616
$2\theta_{max}$	77.7	66.3	66.3
Refl. measured	273030	130201	244432
Refl. indep.	12582	8392	15663
R_{int}	0.033	0.039	0.037
Parameters	314	314	610
Restraints	22	85	209
$wR(F^2$, all refl.)	0.069	0.066	0.123
$R(F, >4\sigma(F))$	0.025	0.027	0.041
S	1.13	1.02	1.02
Max. Δp (e Å^{-3})	2.0, −0.5	0.82, −0.90	0.56, −0.27

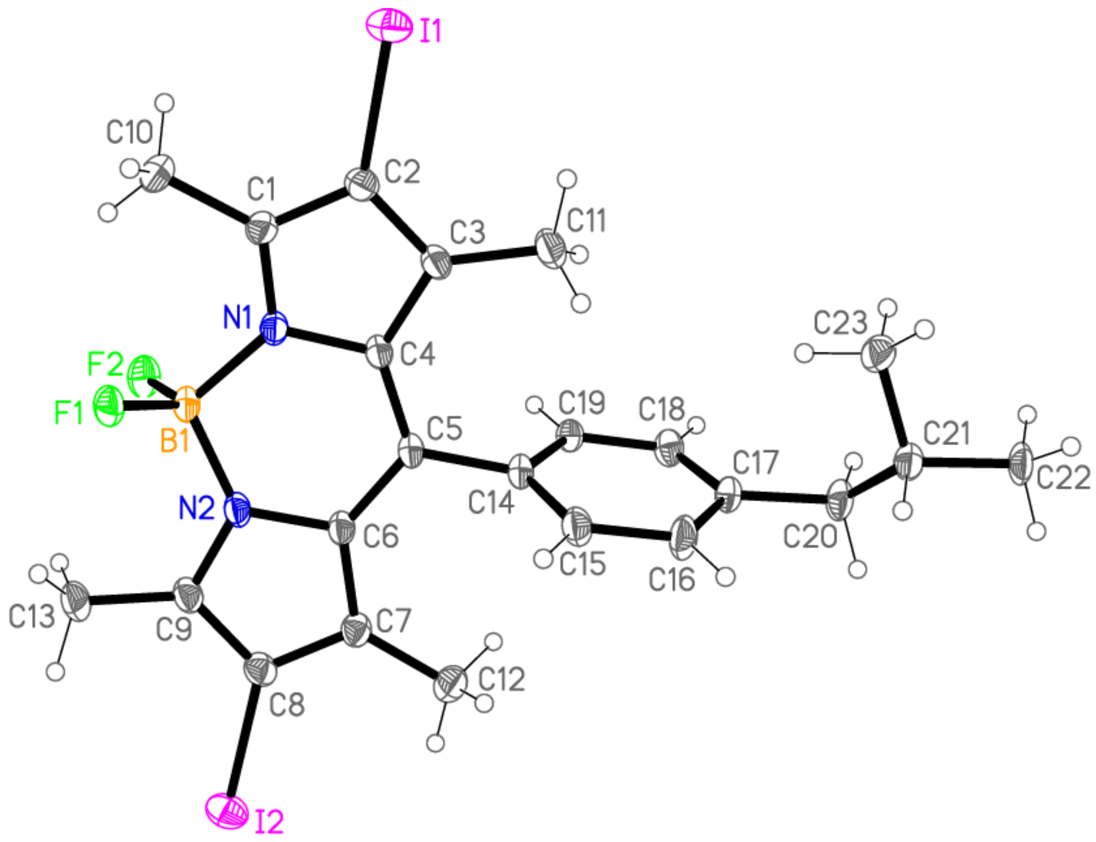

Figure S49: The molecule of compound **1b** in the crystal. Ellipsoids correspond to 50% probability levels. Only the main position of the disordered *iso*-butylphenyl group is shown.

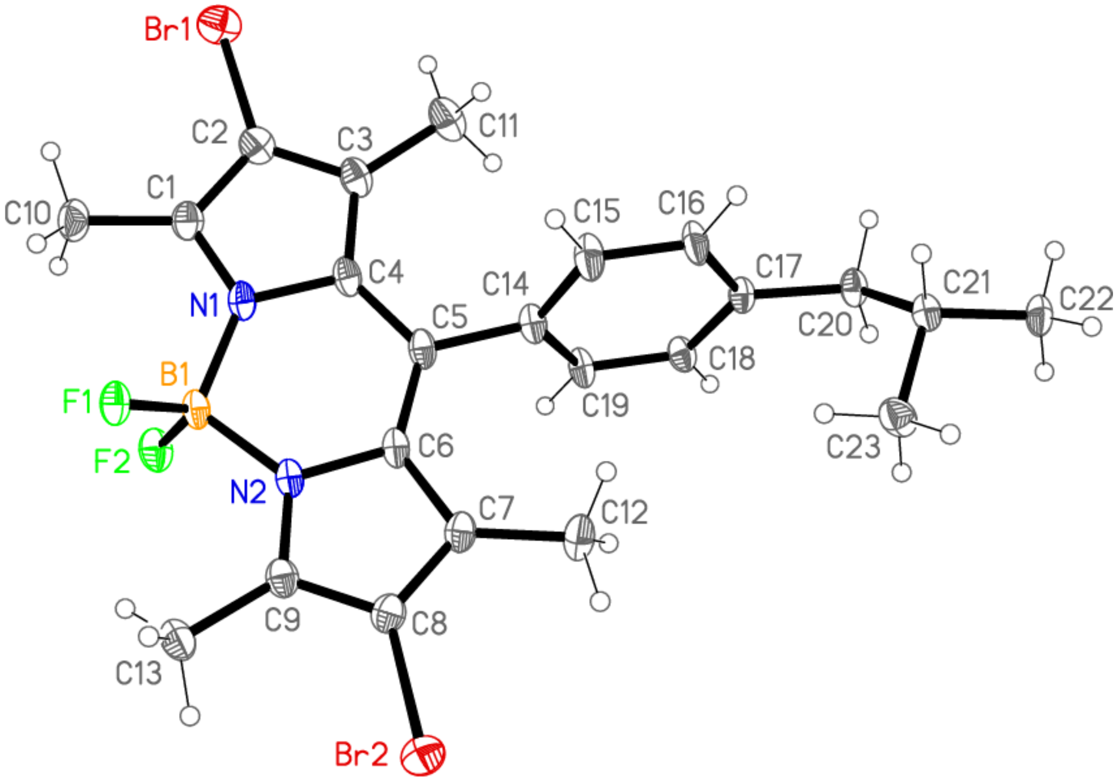

Figure S50: The molecule of compound **1b-*Br*** in the crystal. Ellipsoids correspond to 50% probability levels. Only the main position of the disordered *iso*-butylphenyl group is shown.

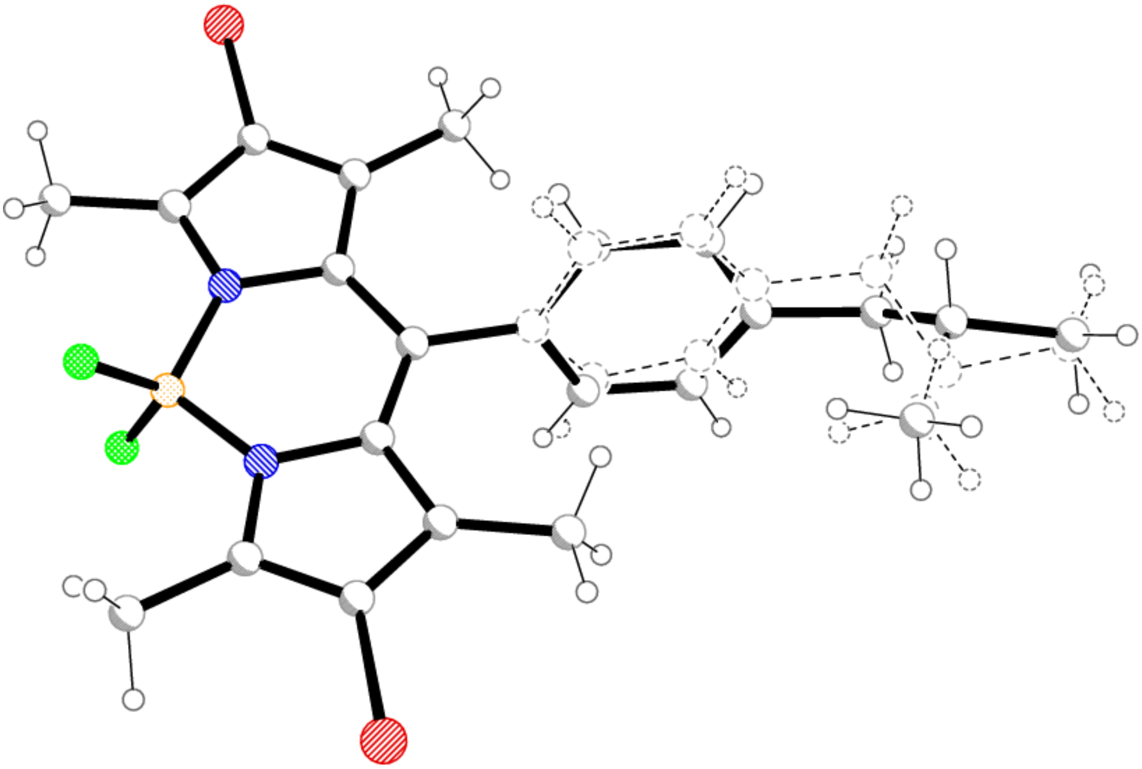

Figure S51: The molecule of compound **1b-*Br*** in the crystal, showing both positions of the disordered *iso*-butylphenyl group. Radii are arbitrary.

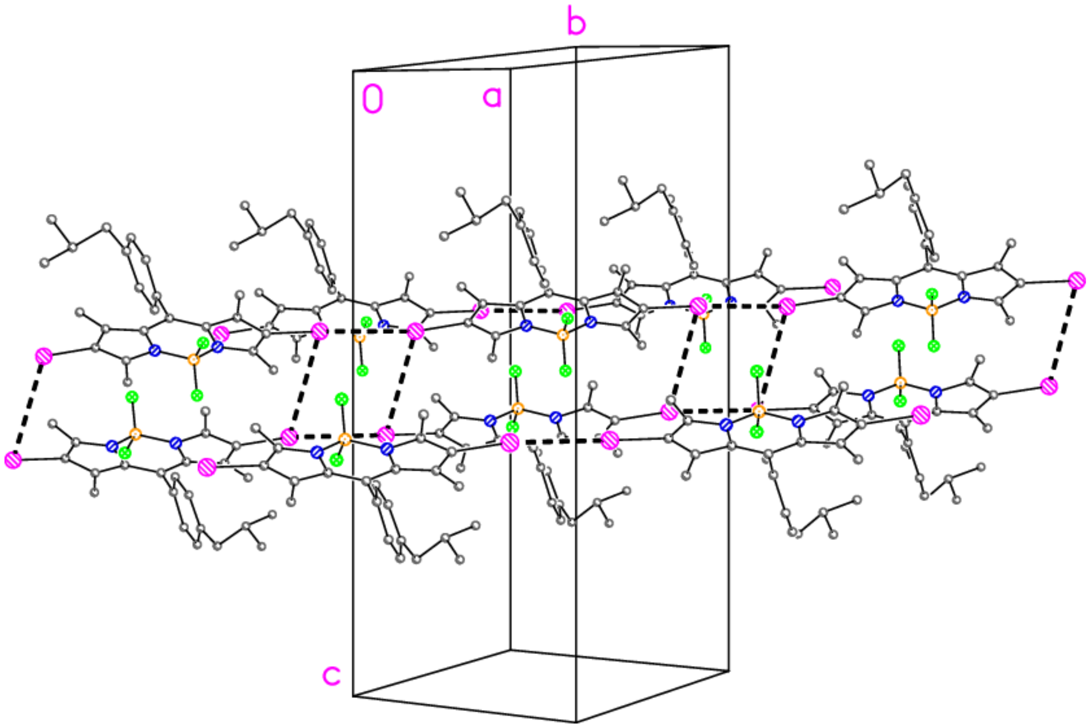

Figure S52: Packing diagram of compound **1b-*Br*** in the crystal, showing the formation of a one-dimensional array involving the contacts I1…I2 (−1+*x*, −1+*y*, *z*) 3.8915(1) and I1…I2 (1−*x*, 1−*y*, 1−*z*) 4.1081(1) Å, shown by thick dashes. Hydrogen atoms are omitted; radii are arbitrary. The array propagates parallel to [1 1 0], and the view direction is perpendicular to (−1 1 0).

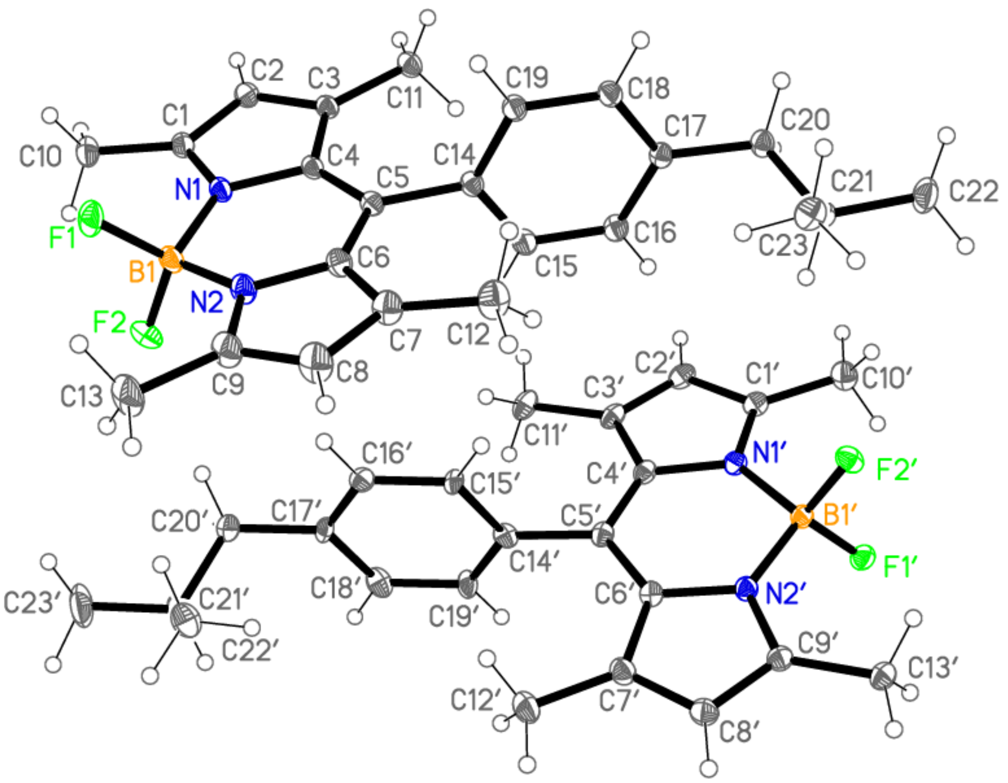

Figure S53: The structure of compound **1b-*H*** in the crystal. The asymmetric unit contains two independent molecules. Ellipsoids correspond to 50% probability levels. Only the main position of the disordered *iso*-butylphenyl group is shown.

9 Computational Studies

9.1 Methods

All calculations were performed with the ORCA program (versions 5.0.4 or 6.0.1).[11] Structures were pre-optimized by Grimme's tight-binding method XTB[12] as implemented in the ORCA program suite. In order to identify the global minimum, the pre-optimized structures were subjected to CREST.[13] The global minimum geometry was further optimized by r^{2}scan-3c,[14] followed by M062X/Def2-SVP/D3.[15] Single point calculations were performed with the hybrid functional M062X, Def2-TZVP as basis set and D3 as dispersion correction.[16] All structures were confirmed as minima by frequencies analyses. For thermodynamic calculations the temperature was set to 298 K and the quasi-RRHO approach for low energy frequencies (below 35 cm^{-1}) was chosen.[17] All calculations were performed in the gas phase. TDDFT computations were run on optimized structures for 10 excited states with the M062X functional and the Def2-TZVP basis set. For excited state analysis and CDD plots the wavefunction analyzer *multiwfn* was used.[18] The *iso*-butyl group of the *meso*-substituent was replaced by hydrogen for simplification.

The SCF convergence criteria were set to tight (Keyword: TightSCF) and DefGrid3 was used as integration grid in ORCA.

9.2 Results

Calculation and Comparison of the Strain Energies

The strain energies of both truncated BODIPY-doped CPPs **4a** and **4b** were calculated by applying the homodesmotic reaction shown in Scheme S1. With 55.3 kcal/mol the strain energy of **4b** is slightly higher than of **4a**, for which a strain energy of 51.6 kcal/mol was calculated.

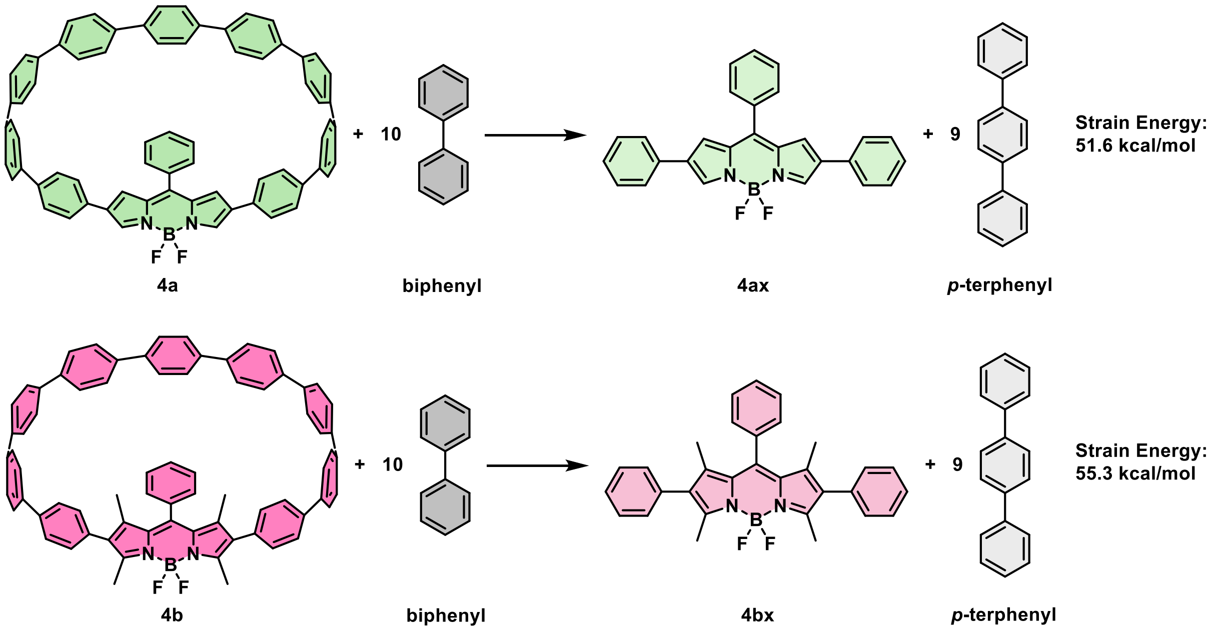

Scheme S1: Simulated homodesmotic reaction of truncated BODIPY-CPPs **4a** (top) and **4b** (bottom) with biphenyl to arylated BODIPYs **4ax**, **4bx** and *p*-terphenyl for ring strain calculation.

Table S3: Calculated electronic energies.

Species	*E* (M062X/Def2-SVP) [Eh]	*E* (M062X/Def2-TZVP) [Eh]	G^{RRHO}_{298} [kcal/mol]
**4a**	−2987.33094402	−2990.57476372	546.3
**4b**	−3144.40665450	−3147.82184716	613.0
**biphenyl**	−462.76439879	−463.26811869	94.1
**4ax**	-	−1374.53000489	217.9
**4bx**	−1530.10073251	−1531.78293799	284.9
***p*-terphenyl**	−693.55985719	−694.31201788	141.5

[a] Due to an imaginary frequency in the optimization run with M062X/Def2-SVP, geometry optimization was performed with M062X/Def2-TZVP.

Analysis of Optimized Geometries and Molecular Orbitals

The additional methyl groups in the α- and the β'-position of the BODIPY unit of molecule **4b** resulted in a rotation of the BODIPY unit out of plane of 44°, whereas the unsubstituted BODIPY unit is only twisted by 17°.

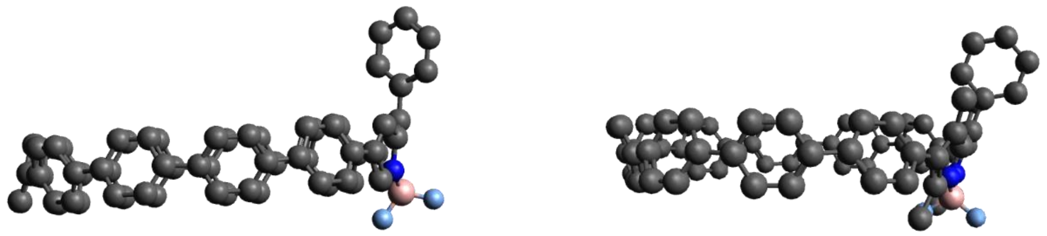

4a **4b**

Figure S54: Optimized geometries of **4a** and **4b.**

Another result of this twist was observed in the contribution to the orbitals. In the case of **4a** the HOMO, mainly centered at the BODIPY unit, showed considerable coefficients at the phenyl rings of the CPP. In contrast, the phenyl rings of **4b** only contribute minorly to the HOMO. The LUMOs of both CPPs **4a** and **4b** are only centered at the BODIPY unit.

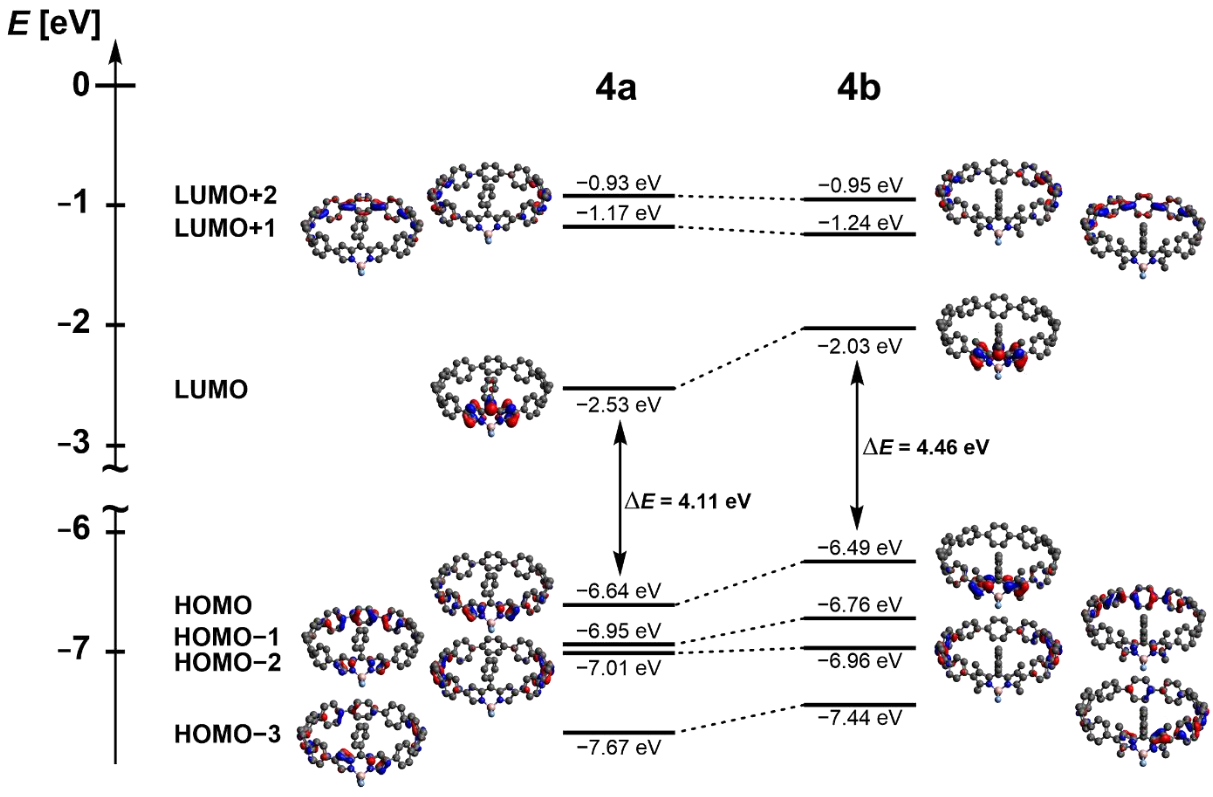

Figure S55: Calculated energies and pictorial presentation of frontier orbitals of BODIPY-doped nanohoops **4a** and **4b**. The *iso*-butyl groups of the *meso*-substituents were replaced by hydrogens, which were all omitted for simplification. Geometry (DFT): M062X/Def2-TZVP. All computations *in vacuo*.

TDDFT Calculations

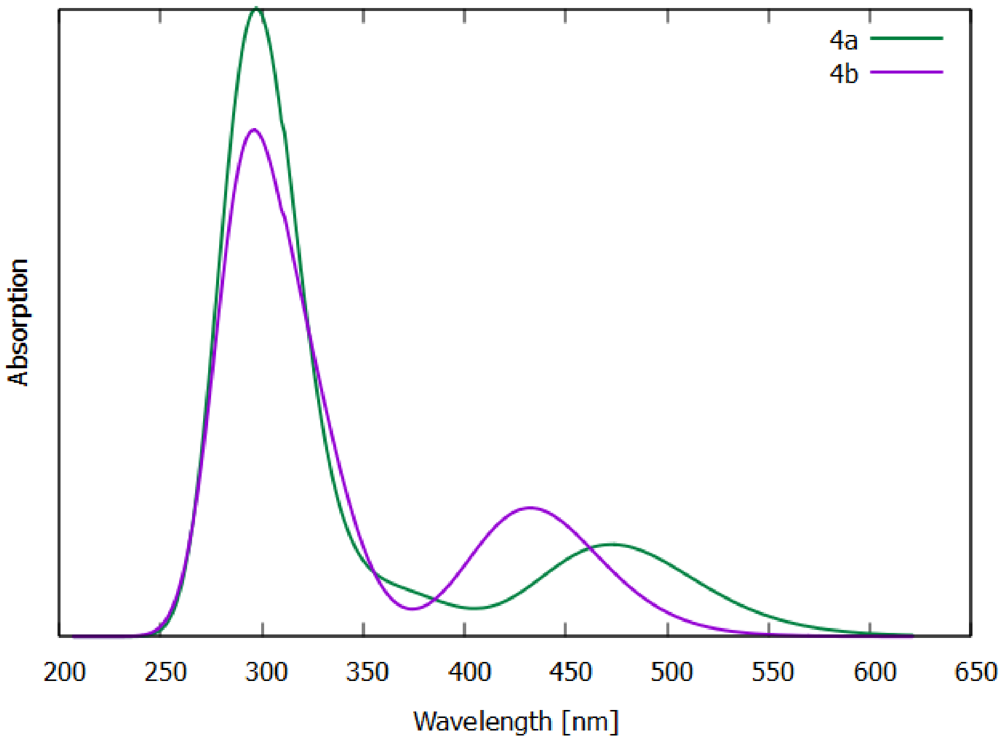

Figure S56: Calculated absorption spectra of compounds **4a** and **4b** (M062X/Def2-TZVP/D3, gas phase).

BODIPY-CPP 4a

Table S4: Excitation energies *E*, respective wavelengths λ, oscillator strengths *f* and main transitions of **4a**.

State	*E* [eV]	λ [nm]	*f*	Main transition
**S_1**	2.629	471.7	0.647869162	230 → 248: 0.010547<br>245 → 250: 0.010932<br>246 → 248: 0.209170<br>247 → 248: 0.693667 HOMO → LUMO
**S_2**	3.328	372.5	0.249458846	241 → 248: 0.026814<br>243 → 248: 0.137714<br>244 → 248: 0.072814<br>245 → 248: 0.715942
**S_3**	3.665	338.3	0.183141742	242 → 248: 0.042454<br>243 → 248: 0.054764<br>244 → 248: 0.110979<br>246 → 248: 0.541930<br>247 → 248: 0.219519
**S_4**	3.774	328.5	0.047734155	230 → 248: 0.035636<br>242 → 248: 0.178433<br>243 → 248: 0.018488<br>243 → 252: 0.010347<br>244 → 248: 0.042021<br>245 → 250: 0.124740<br>246 → 248: 0.082545<br>246 → 249: 0.109104<br>246 → 251: 0.035821<br>247 → 249: 0.273068<br>247 → 251: 0.013212
**S_5**	3.979	311.6	1.189802918	230 → 248: 0.023954<br>242 → 248: 0.216691<br>243 → 248: 0.055667<br>244 → 248: 0.160057<br>245 → 250: 0.034515<br>246 → 248: 0.060452

				246 → 249: 0.139453<br>246 → 251: 0.024304<br>247 → 248: 0.048335<br>247 → 249: 0.163620
**S_6**	4.120	300.9	0.520687490	226 → 248: 0.013602<br>228 → 248: 0.026140<br>231 → 248: 0.015697<br>235 → 248: 0.013114<br>241 → 248: 0.081580<br>243 → 248: 0.342752<br>244 → 248: 0.133162<br>245 → 248: 0.209578<br>245 → 249: 0.053062<br>245 → 251: 0.011082<br>247 → 250: 0.051772
**S_7**	4.160	298.0	1.791509937	231 → 248: 0.293687<br>233 → 248: 0.011290<br>241 → 248: 0.025537<br>243 → 248: 0.033856<br>244 → 248: 0.024141<br>245 → 248: 0.046222<br>245 → 249: 0.159801<br>245 → 251: 0.028715<br>246 → 252: 0.019262<br>247 → 250: 0.265228
**S_8**	4.280	289.7	0.821781510	231 → 248: 0.537716<br>232 → 248: 0.011999<br>233 → 248: 0.021628<br>243 → 248: 0.022982<br>245 → 248: 0.013131<br>245 → 249: 0.119481<br>247 → 250: 0.149392
**S_9**	4.349	285.1	0.525821860	225 → 248: 0.010708<br>227 → 248: 0.038129<br>230 → 248: 0.120939<br>231 → 248: 0.013953<br>232 → 248: 0.011939<br>242 → 248: 0.104420<br>243 → 248: 0.116455<br>244 → 248: 0.275514<br>245 → 250: 0.019210<br>246 → 248: 0.058680<br>246 → 249: 0.081603<br>247 → 251: 0.058351
**S_{10}**	4.453	278.5	0.565477227	230 → 248: 0.036211<br>232 → 248: 0.011071<br>233 → 248: 0.012967<br>236 → 248: 0.012830<br>242 → 248: 0.097715<br>243 → 250: 0.014844<br>244 → 248: 0.016973<br>244 → 249: 0.014113<br>245 → 250: 0.065205<br>245 → 252: 0.013048<br>246 → 248: 0.021987<br>246 → 249: 0.235693<br>247 → 249: 0.046162<br>247 → 251: 0.256544

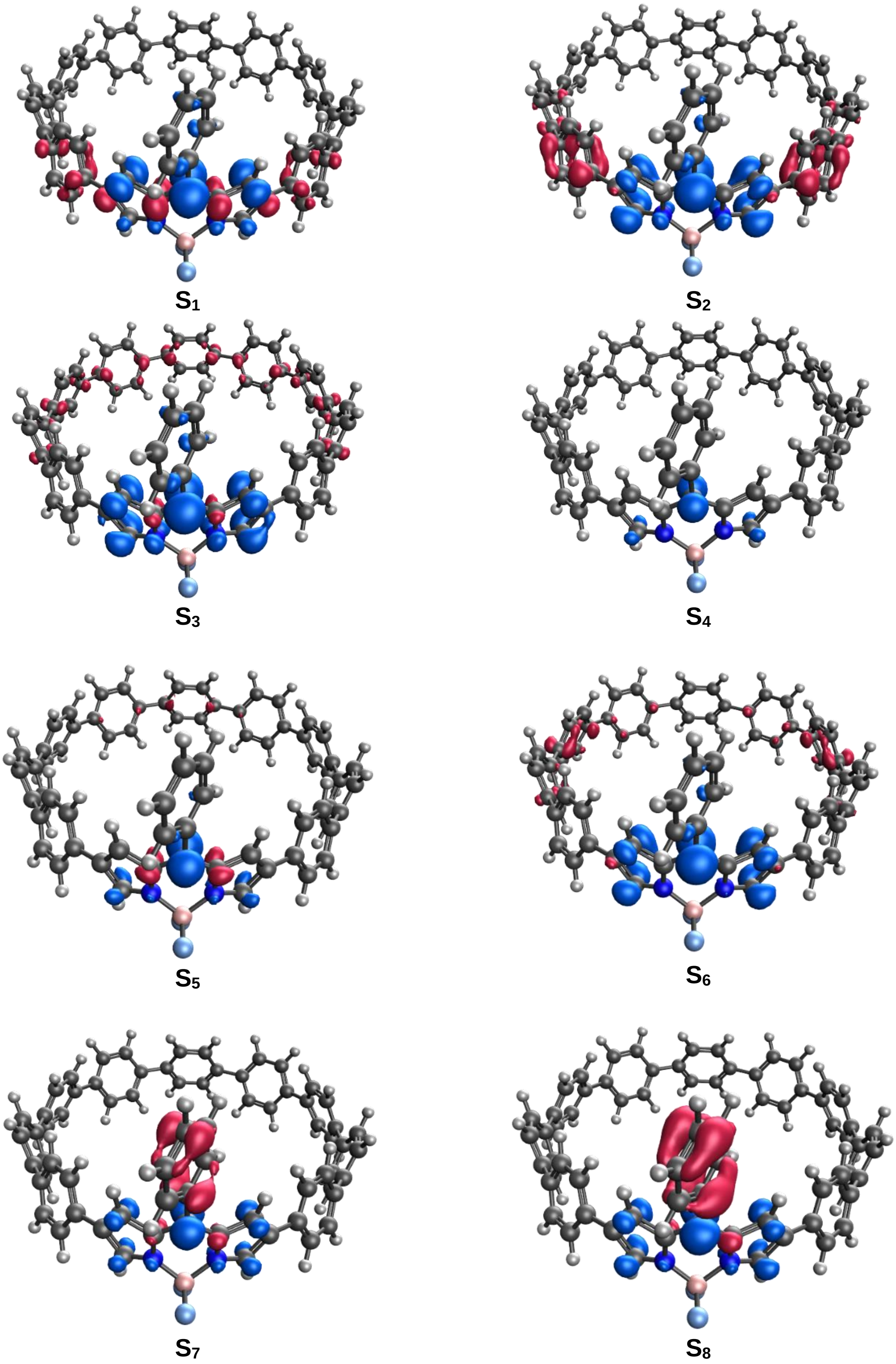

Figure S57: CDD plots of the relevant excited states of **4a** (isovalue = 0.002; red: negative, blue: positive).

Methylated BODIPY-CPP 4b

Table S5: Excitation energies *E*, respective wavelengths *λ*, oscillator strengths *f* and main transitions of **4b**.

State	*E* [eV]	*λ* [nm]	*f*	Main transition
S_1	2.874	431.4	0.905706635	258 → 264: 0.018135<br>261 → 266: 0.015443<br>262 → 264: 0.039180<br>263 → 264: 0.855363 HOMO → LUMO
S_2	3.725	332.8	0.354509358	244 → 264: 0.013335<br>257 → 264: 0.052817<br>259 → 264: 0.285083<br>261 → 264: 0.594859
S_3	3.801	326.2	0.029446867	247 → 264: 0.021581<br>258 → 264: 0.286503<br>260 → 264: 0.379199<br>260 → 267: 0.016663<br>261 → 266: 0.036263<br>262 → 264: 0.092892<br>262 → 265: 0.071064<br>263 → 265: 0.034863
S_4	3.889	318.8	1.183837456	259 → 266: 0.010043<br>260 → 264: 0.068075<br>260 → 267: 0.020369<br>261 → 266: 0.114930<br>262 → 264: 0.177120<br>262 → 265: 0.391693<br>262 → 267: 0.015074<br>263 → 265: 0.133503
S_5	4.178	296.8	0.230488762	247 → 264: 0.031848<br>254 → 264: 0.013131<br>258 → 264: 0.157861<br>260 → 264: 0.017509<br>262 → 264: 0.584764<br>262 → 265: 0.049555<br>263 → 264: 0.082037<br>263 → 265: 0.018329
S_6	4.211	294.4	2.516649875	257 → 264: 0.016668<br>259 → 264: 0.039828<br>259 → 267: 0.010878<br>260 → 266: 0.034004<br>261 → 265: 0.308522<br>261 → 267: 0.055177<br>262 → 266: 0.141417<br>263 → 266: 0.294502
S_7	4.423	280.3	0.038170252	244 → 264: 0.068401<br>250 → 264: 0.011585<br>257 → 264: 0.162580<br>259 → 264: 0.274430<br>261 → 264: 0.353188<br>263 → 266: 0.076627
S_8	4.450	278.6	0.721458511	259 → 266: 0.025633<br>260 → 264: 0.029594<br>260 → 265: 0.068796<br>261 → 266: 0.063410<br>262 → 265: 0.197722<br>262 → 267: 0.016802<br>263 → 265: 0.229311<br>263 → 267: 0.249911
S_9	4.530	273.7	0.002116998	246 → 264: 0.891408<br>247 → 264: 0.011234<br>254 → 264: 0.018377<br>260 → 264: 0.010277
S_{10}	4.663	265.9	0.071059158	257 → 264: 0.010275<br>259 → 264: 0.015505<br>259 → 265: 0.069652<br>261 → 264: 0.012748<br>261 → 265: 0.093158

				262 → 266: 0.233598<br>263 → 266: 0.274845<br>263 -> 268: 0.131266<br>263 -> 270: 0.016185

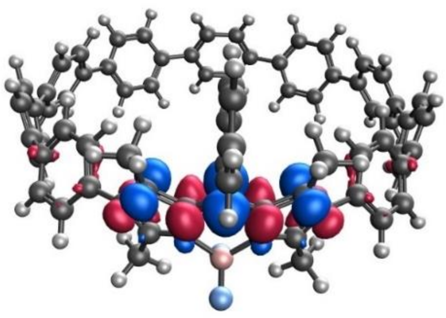

S_1

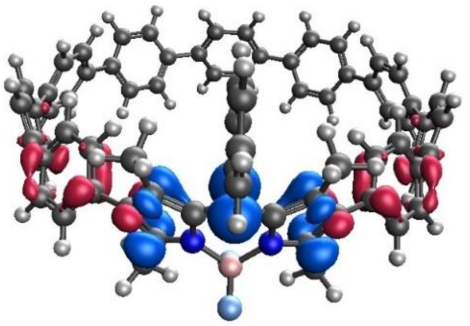

S_2

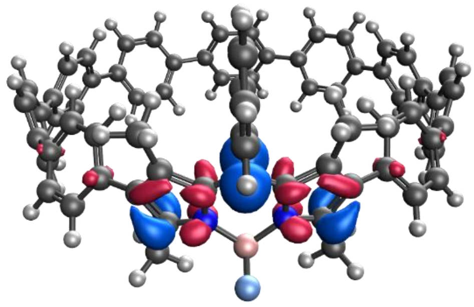

S_3

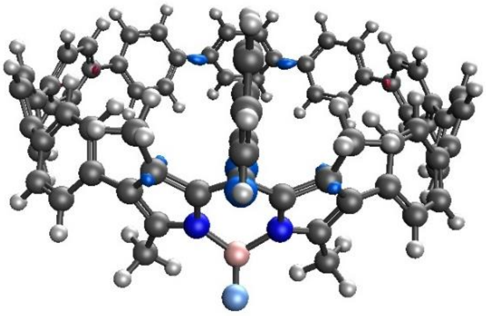

S_4

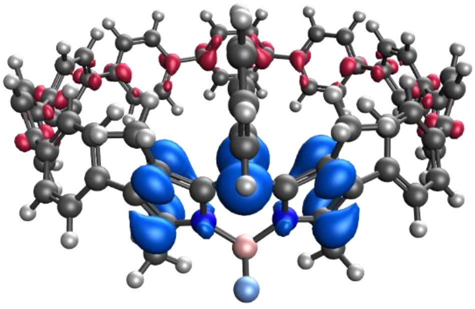

S_5

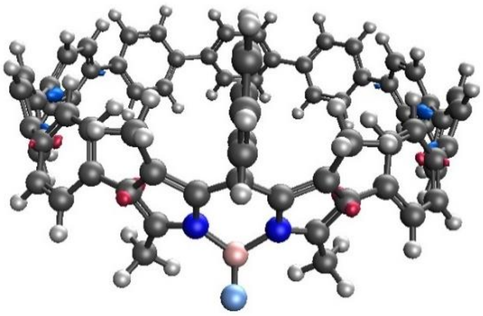

S_6

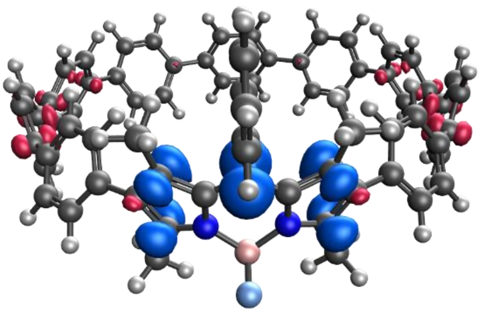

S_7

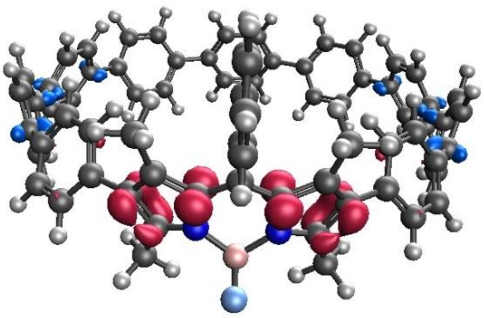

S_8

Figure S58: CDD plots of the relevant excited states of **4b** (isovalue = 0.002 unless otherwise noted; red: negative, blue: positive).

DFT-optimized cartesian coordinates (M062X/def2-SVP/D3)

4a

E (M062X/Def2-SVP) = −2987.33094402 Eh

Lowest Freq. = 12.35 cm^{-1}

C	−6.70167263	−1.342282202	0.000937809
N	−6.404680747	−1.336557735	−1.353466865
N	−6.575776379	1.110018303	−1.383332771
C	−6.848894824	1.08813701	−0.027056623
C	−6.989749143	−0.133060235	0.662566577
C	−6.059038786	2.293154825	−1.691504618
C	−5.958575876	3.111403869	−0.533436407
C	−6.496319868	2.354725882	0.507757042
C	−6.330943127	−2.611711249	0.517716748
C	−5.73759428	−3.327356903	−0.522199604
C	−5.844960439	−2.495558455	−1.67047242
B	−6.73954629	−0.139294805	−2.315139513
F	−8.033091558	−0.243180736	−2.758544103
F	−5.821774571	−0.086365981	−3.333846066
C	8.552452351	1.576503057	0.335417307
C	9.091710055	0.865292164	−0.748695904
C	9.125925882	−0.525482388	−0.746456347
C	8.622293011	−1.258950844	0.339967627
C	8.244241336	−0.543245005	1.484831918
C	8.209972469	0.846683115	1.482571777
C	8.085311851	2.981456437	0.212015326
C	−3.423075734	5.549832431	0.776955613
C	−4.474367181	4.642293919	0.751162366
C	−5.121861286	4.32120377	−0.450699403
C	−4.771035199	5.054206114	−1.595155449
C	−3.716798397	5.961769696	−1.56843377
C	−2.973743839	6.174053367	−0.396910823
C	0.569284333	6.876322714	−1.439839312
C	−0.794163883	6.617383051	−1.518684721
C	−1.633808838	6.814808898	−0.413042337
C	−1.076494556	7.420544723	0.724702568
C	0.288769214	7.676690511	0.804967456
C	1.150284369	7.340540169	−0.251177433
C	4.815460722	6.854604039	−1.056156439
C	3.519818156	7.353673699	−1.142441155
C	2.61971497	7.215498778	−0.073720694
C	3.118186064	6.703249305	1.132314761
C	4.412262348	6.201858358	1.217475138
C	5.260344538	6.194753909	0.100858792
C	7.509007702	3.398180711	−0.996369949
C	6.701319411	4.528095544	−1.053656101
C	6.437328495	5.287833454	0.094783628
C	7.145938555	4.964245089	1.263180596
C	7.954031392	3.832972193	1.320747534
C	8.133250408	−3.538782842	1.332221348
C	7.379388969	−4.707055193	1.278048973
C	6.686944774	−5.067180672	0.110630082
C	6.91613892	−4.300137887	−1.040452368
C	7.669658737	−3.133301331	−0.986547439
C	5.551403388	−6.025460414	0.119664166
C	4.70389615	−6.065943544	1.236030494
C	3.431472091	−6.620148635	1.150962277
C	2.955024113	−7.153480234	−0.054820663
C	3.86126369	−7.258052074	−1.122161377
C	5.135368594	−6.706442588	−1.035853185
C	1.491569175	−7.332741864	−0.235083375
C	0.63985716	−7.699813398	0.81876386
C	−0.733280171	−7.490557079	0.735807988

C	−1.30854882	−6.902251312	−0.402220904
C	−0.473933034	−6.675990753	−1.506128679
C	0.897281741	−6.888456329	−1.424789228
C	−2.668209314	−6.304315849	−0.3879041
C	−3.410811704	−6.105865018	−1.562375485
C	−4.490064058	−5.228478366	−1.589805911
C	−4.86872645	−4.513838255	−0.44250461
C	−4.21988375	−4.825406637	0.761107988
C	−3.142943227	−5.702504466	0.787613205
C	8.224185111	−2.685378136	0.220830148
C	−7.263912059	−0.091942908	2.117132869
C	−6.385352464	−0.667105492	3.046405384
C	−6.660773067	−0.591825846	4.409700123
C	−7.814943101	0.050768106	4.858217972
C	−8.687474405	0.633934391	3.939636815
C	−8.410295546	0.57284515	2.575860855
H	−5.718218107	2.494206106	−2.706368098
H	−6.548650094	2.617737312	1.562074393
H	−6.438399356	-2.92708555	1.552187702
H	−5.494977603	−2.676741892	−2.686030228
H	9.43474154	1.408024703	−1.631870639
H	9.494946645	−1.053591975	−1.628019087
H	7.846522305	−1.079316368	2.348124793
H	7.786399939	1.365356541	2.344188353
H	−2.86911365	5.689600738	1.7066165
H	−4.723314383	4.095213894	1.661863386
H	−5.296830652	4.873852835	−2.534937843
H	−3.434682759	6.479075378	−2.487411797
H	1.209563596	6.59848049	−2.278725594
H	−1.18929452	6.142480955	−2.418058534
H	−1.714893885	7.650202875	1.58012544
H	0.701897793	8.100596189	1.722531954
H	5.47632679	6.928063269	−1.922154532
H	3.181952226	7.810796414	−2.074717944
H	2.445830846	6.579190484	1.982972825
H	4.722953655	5.695284131	2.132687259
H	7.587275331	2.760334074	−1.878277475
H	6.166942615	4.747619423	−1.979355969
H	7.017502313	5.576580065	2.15803207
H	8.446804783	3.57406972	2.260111546
H	8.61260016	−3.253757045	2.270955455
H	7.279334889	−5.321637595	2.174990969
H	6.393243449	−4.547544474	−1.965659846
H	7.718201352	−2.495510518	−1.870611273
H	4.992636308	−5.545036852	2.150400502
H	2.753930952	−6.521831394	2.000880045
H	3.543100858	−7.73024111	-2.05386321
H	5.799322996	−6.754696644	−1.901240145
H	1.065362433	−8.109943562	1.736936032
H	−1.365004459	−7.743081986	1.589740701
H	−0.882693879	−6.213790792	−2.405981198
H	1.52929239	−6.588169075	−2.262185229
H	−3.108722849	−6.608531342	−2.483068371
H	−5.015258567	−5.056618383	−2.53148835
H	−4.490411108	−4.291442393	1.673665476
H	−2.590634909	−5.832492752	1.719643941
H	−5.466907602	−1.140264298	2.696156133
H	−5.964936645	−1.030180802	5.126357038
H	−9.589153065	1.140872858	4.285646834
H	−9.088835143	1.027075754	1.851674121
H	−8.030241476	0.104051928	5.926323143

4b

E (M062X/Def2-SVP) = −3144.40665450 Eh

Lowest Freq. = 12.13 cm^{-1}

C	−6.180496924	−1.236423166	0.235239102
N	−5.620387872	−1.261395609	1.50914191
N	−5.626496732	1.237472538	1.508949028
C	−6.186255204	1.209561842	0.234955351
C	−6.512046352	−0.014272489	−0.369846156
C	−5.158680448	2.46451654	1.750186165
C	−5.36921515	3.28620334	0.600754419
C	−6.063399294	2.515974058	−0.335689714
C	−6.051880888	−2.542457371	−0.334997673
C	−5.354110017	−3.309255156	0.60158436
C	−5.147052584	−2.486269027	1.750714166
B	-5.52460905	−0.011645158	2.45239852
F	−4.281312672	−0.008387638	3.067340284
F	−6.545287038	−0.014181828	3.375249772
C	8.682756336	1.4378567	−0.295975105
C	9.156385048	0.715634174	0.811082795
C	9.159399077	−0.675897329	0.811032604
C	8.688852819	−1.400092925	−0.296043302
C	8.36639492	−0.676839791	−1.453213996
C	8.363423359	0.71327316	−1.453185561
C	8.268587952	2.861745658	−0.202403847
C	−2.874948488	5.613189955	−0.942144524
C	-3.87868577	4.661925431	−0.817509624
C	−4.625679685	4.542304591	0.365920328
C	−4.426393382	5.518748081	1.355645291
C	−3.407571155	6.462909944	1.238165282
C	−2.564668761	6.478787933	0.117500097
C	0.732842876	7.839278778	−1.132195427
C	−0.638529539	7.61560842	−1.067370888
C	−1.238183145	7.147694586	0.112471797
C	−0.438906496	7.072866237	1.26162374
C	0.933486881	7.288501086	1.194129851
C	1.559057941	7.594342382	−0.023056293
C	4.784867372	6.219509913	−1.366251885
C	3.518590009	6.79138521	−1.330186644
C	3.024232976	7.385367273	−0.159957569
C	3.910940788	7.521565563	0.920614024
C	5.177167576	6.945138778	0.886481187
C	5.608856419	6.214517979	−0.231925812
C	8.219151733	3.704171367	-1.324838903
C	7.466218506	4.873841957	-1.309639696
C	6.735282284	5.247870268	-0.170687772
C	6.9185641	4.491037263	0.995186894
C	7.669788293	3.32097691	0.979365438
C	7.684438253	−3.287722123	0.979407831
C	6.938364111	−4.461077745	0.995372646
C	6.757962188	−5.218569319	−0.1705321
C	7.486866679	−4.841214956	−1.30968789
C	8.234683428	−3.668272613	−1.325014222
C	5.635794068	−6.190183469	−0.231522822
C	5.20759647	−6.92271295	0.886982511
C	3.94394362	−7.504753746	0.921399016
C	3.056397775	−7.372482957	−0.158972787
C	3.54786769	−6.776323663	−1.329320026
C	4.811576175	−6.198823615	−1.365664878
C	1.592185796	−7.587935818	−0.021775584
C	0.9654863	−7.284636507	1.195461363
C	−0.407818959	−7.074944578	1.263150077
C	−1.206960321	−7.153514295	0.114158454
C	−0.605471824	−7.619047091	−1.065692558
C	0.766855398	−7.836691975	−1.130731073
C	−2.536259272	−6.490224925	0.119202581
C	−2.850433714	−5.626487453	−0.940805822
C	−3.858097663	−4.679351158	−0.816401691
C	−4.605276479	−4.562257387	0.36715887
C	−4.401641468	−5.537329069	1.357349567
C	−3.378925404	−6.47728538	1.240089914
C	8.280827711	−2.825749763	−0.20251164
C	−7.096170246	−0.015803226	−1.740453051
C	−8.484114413	−0.018803845	−1.906415795
C	−9.033568977	−0.020204583	−3.188465877
C	−8.200441288	−0.018596974	−4.30734215
C	−6.814792058	−0.015572829	−4.143145832
C	−6.263724897	−0.014182316	−2.863228761
C	−4.525844121	2.872407587	3.038960581
C	−6.541519946	3.005492923	−1.669631342
C	−6.528198794	−3.034592176	−1.668622521
C	−4.512288316	−2.890941219	3.039564125
H	9.477438779	1.251519817	1.706591188
H	9.482770001	−1.210447581	1.706508496
H	7.991976191	−1.205178811	−2.331494232
H	7.986746679	1.240049663	−2.331440149
H	−2.237217227	5.598952029	−1.827735989
H	−4.007562966	3.920724368	−1.607665067
H	−5.037095576	5.501702052	2.259265794
H	−3.229562964	7.165226595	2.055347854
H	1.179067291	8.151329702	−2.078453442
H	−1.24813082	7.74918854	−1.963514578
H	−0.874130222	6.712836886	2.195698628
H	1.541374313	7.090173702	2.078271638
H	5.086082135	5.658309986	−2.252375369
H	2.858880894	6.662510896	−2.189511184
H	3.584141132	8.039302159	1.824564164
H	5.822791436	7.017094095	1.764166385
H	8.733115116	3.409818087	−2.242084911
H	7.399050417	5.480232738	−2.215211431
H	6.363342935	4.750992598	1.89808795
H	7.685055293	2.691499221	1.870618991
H	7.697276158	−2.658312062	1.870749317
H	6.384643902	−4.723573189	1.898465721
H	7.422091941	−5.447817038	−2.215292632
H	8.747097183	−3.371623417	−2.242385618
H	5.853731873	−6.991839122	1.764518125
H	3.619660393	−8.023949029	1.825416925
H	2.887405408	−6.650362079	−2.188497283
H	5.110092076	−5.636280715	−2.251851044
H	1.572651513	−7.083469045	2.079451379
H	−0.844418128	−6.716597833	2.197229832
H	−1.214627757	−7.755421462	−1.961720428
H	1.214270068	−8.146951563	−2.077012319
H	−2.213009825	−5.610037727	−1.826580865
H	−3.990332723	−3.93910507	−1.606909778
H	−5.012166757	−5.522332489	2.261129526
H	−3.19776526	−7.178423301	2.057595603
H	−9.129258426	−0.020036904	−1.025758094
H	−10.11733575	−0.022566699	−3.313284764
H	−6.160542218	−0.014286744	−5.01628463
H	−5.179913447	−0.011779566	−2.7244077
H	−8.63167108	−0.019710146	−5.309346267
H	−7.562858059	2.66308659	−1.881899269
H	−5.910141394	2.646917605	−2.496459455
H	−6.526973563	4.103401661	−1.683073942
H	−6.508989802	−4.132438767	−1.681573821
H	−5.898593342	−2.673692226	−2.495775256
H	−7.551075768	−2.696680825	−1.880742413
H	−4.243340718	−2.020469533	3.641320329
H	−3.613863323	−3.491044462	2.84057686
H	−5.207435443	−3.518932818	3.617344643
H	−5.22342742	3.498355888	3.616009785
H	−3.629530356	3.475622581	2.839823727
H	−4.253872212	2.003373448	3.641451894

Biphenyl

E (M062X/Def2-SVP) = −462.76439879 Eh

Lowest Freq. = 71.27 cm^{-1}

C	−1.186537471	2.854255458	0.208657130
C	−1.186187453	1.461058304	0.208749535
C	−0.000001793	0.743318583	0.000002603
C	1.186182709	1.461062901	−0.208749973
C	1.186538400	2.854261784	−0.208656922
C	−0.000001724	3.556656341	0.000000628
C	0.000004121	−0.743331091	0.000002141
C	1.063663356	−1.461086335	0.565010542
C	1.064010854	−2.854278443	0.565046521
C	−0.000001795	−3.556682091	−0.000003944
C	−1.064012217	−2.854273861	−0.565051799
C	−1.063656368	−1.461080906	−0.565006003
H	−2.119280899	3.393485081	0.381538155
H	−2.114955292	0.919638871	0.398318089
H	2.114948657	0.919643557	−0.398322410
H	2.119286018	3.393485704	−0.381534727
H	−0.000009009	4.647598631	0.000004439
H	1.888497866	−0.919607187	1.032069558
H	1.897812356	−3.393459799	1.017524516
H	−0.000001109	−4.647624859	−0.000005932
H	−1.897814949	−3.393451986	−1.017530252
H	−1.888484258	−0.919588659	−1.032061896

CS2a

E (M062X/Def2-TZVP) = −1374.53000489 Eh Eh

Lowest Freq. = 13.78 cm^{-1}

C	−0.156120656	1.211329171	0.042052792
N	−1.540063619	1.247885287	0.041784745
B	−2.50066168	0.01508414	−0.018951359
N	−1.554336981	−1.2293796	−0.064026213
C	−0.170177759	−1.209608985	−0.04198208
C	0.533808127	-0.00339486	0.005834082
C	−1.949869864	−2.495219841	−0.059004475
C	−0.837407175	−3.365885733	−0.034165996
C	0.281209025	−2.545835571	−0.017553487
C	0.311801901	2.541953396	0.024173575
C	−0.796937851	3.375542438	0.022377094
C	−1.920096504	2.518410668	0.029826727
F	−3.286286484	−0.036190215	1.10957408
F	−3.266855207	0.076060244	−1.160273351
C	2.009264395	−0.012227485	0.017647326
C	2.710817654	0.676096608	1.008409666
C	4.096899442	0.660960943	1.021326279
C	4.796105614	−0.02842137	0.039515685
C	4.104435742	−0.709781504	−0.953145899
C	2.718239227	−0.708823485	−0.961977669
C	−0.887109462	−4.831311949	−0.021333778
C	−0.828649098	4.841456718	0.007155706
C	0.185006448	−5.583872384	−0.50338362
C	0.144215961	−6.968899693	−0.485251415
C	−0.973305978	−7.628858523	0.008723913
C	−2.047577984	−6.891100953	0.487324381
C	−2.00386853	−5.505702182	0.475272207
C	−1.929815801	5.52864213	−0.506466689
C	−1.95641828	6.914439448	−0.521217367
C	−0.880178459	7.63985189	−0.028422607
C	0.221949698	6.967102264	0.482622142
C	0.245541673	5.581720948	0.503426404
H	−3.001824173	−2.737976128	−0.083398382
H	1.312537447	−2.852445579	0.048004177
H	1.34769199	2.835972645	−0.025064707
H	−2.969302548	2.773833829	0.036967006
H	2.161141156	1.200612191	1.779488972
H	4.631679898	1.185578397	1.802041788
H	5.878105396	−0.034693056	0.048010608
H	4.645276264	−1.240665258	−1.725405645
H	2.174675687	−1.226982081	−1.741639376
H	1.049786409	−5.07622413	−0.912388322
H	0.984059228	−7.535559603	−0.866023331
H	−1.006920667	−8.710232088	0.019801602
H	−2.921011035	−7.396062222	0.878945587
H	−2.837259851	−4.940682064	0.874055656
H	−2.764267225	4.973243146	−0.916377676
H	−2.817875865	7.429338051	−0.926194899
H	−0.900361622	8.721537989	−0.041687514
H	1.063150794	7.524095158	0.874483481
H	1.098161181	5.064288844	0.925490172

CS2b

E (M062X/Def2-SVP) = −1530.10073251 Eh

Lowest Freq. = 19.88 cm^{-1}

C	0.097314303	1.212484351	0.113769151
N	−1.282386613	1.300229981	0.253726907
B	−2.290283744	0.111686157	0.19509781
N	−1.426788064	−1.168679642	−0.021170662
C	−0.040617876	−1.217030152	−0.106955548
C	0.714566682	−0.036100818	−0.052447631
C	−1.912469301	−2.410029867	−0.066045632
C	−0.835576749	−3.332857772	−0.188434255
C	0.349059352	−2.589848219	−0.207647616
C	0.629894079	2.540216748	0.135771311
C	−0.464359451	3.395277151	0.300100598
C	−1.631361847	2.582789757	0.363350719
F	−2.988644491	−0.000230972	1.386661822
F	−3.163349471	0.302171329	−0.863635487
C	2.194605634	−0.108210427	−0.175333145
C	2.784367371	−0.34254792	−1.421394794
C	4.172092616	−0.402476863	−1.537272452
C	4.975971203	−0.241476558	−0.408252061
C	4.389493386	−0.013914165	0.837181745
C	3.002421678	0.059159396	0.953712856
C	−0.973071555	−4.800231721	−0.271112783
C	−0.444533026	4.868880226	0.384381705
C	−0.307356966	−5.528703591	−1.268697818
C	−0.434015802	−6.914042906	−1.34610469
C	−1.235800119	−7.596872781	−0.431662437
C	−1.906920235	−6.885116957	0.562572098
C	−1.77392559	−5.500491593	0.644083751
C	−1.307811308	5.64498668	−0.404194865
C	−1.292478263	7.035846718	−0.322462728
C	−0.408450038	7.677565566	0.544803328
C	0.457216852	6.918713491	1.332321098
C	0.436431487	5.527651406	1.255438983
H	2.146950115	−0.471888417	−2.298636517
H	4.627984553	−0.577554395	−2.512861082
H	6.061810021	−0.293534223	−0.499111044
H	5.015507686	0.109325949	1.722159483
H	2.534601412	0.241993845	1.923401466

H	0.302439829	−4.993836263	−1.999496284
H	0.089236806	−7.462615209	−2.130931828
H	−1.338099511	−8.681241395	−0.49413486
H	−2.532184577	−7.412077288	1.285038053
H	−2.283689804	−4.949383531	1.436984333
H	−1.986320988	5.146961555	−1.099817434
H	−1.969640634	7.622014158	−0.945726217
H	−0.394681274	8.766655752	0.607393492
H	1.147429991	7.412867448	2.017963289
H	1.099173649	4.935106351	1.88924592
C	2.049995056	2.982964719	−0.048775785
H	2.557605426	2.407193731	−0.833317853
H	2.071888635	4.046078915	−0.322248967
H	2.641271182	2.858608646	0.871201929
C	−3.05325407	3.001869476	0.528294939
H	−3.57400463	2.296728721	1.189046787
H	−3.109606817	4.015879644	0.94156353
H	−3.571396895	2.981934055	−0.442630232
C	−3.376526575	−2.686126037	0.003978848
H	−3.925441982	−1.925615542	−0.566319588
H	−3.599049014	−3.68472802	−0.390280706
H	−3.725689451	−2.627375522	1.046204369
C	1.730621586	−3.169588669	−0.252079618
H	2.411175654	−2.652730677	0.436666932
H	1.693442861	−4.232313442	0.021203735
H	2.175217628	−3.094370363	−1.256128371

***p*-Terphenyl**

E (M062X/Def2-SVP) = −693.55985719 Eh

Lowest Freq. = 40.34 cm^{-1}

C	−0.880577228	5.005690926	−0.876340431
C	−0.867719533	3.612794869	−0.874926771
C	−0.02805817	2.902803049	−0.004197719
C	0.79819456	3.629551123	0.86560519
C	0.785433804	5.022423874	0.864856118
C	−0.053990554	5.716311996	−0.006351515
C	−0.014006328	1.418178819	−0.002777058
C	1.180604171	0.706889381	0.178541721
C	1.193994178	−0.683769914	0.179497718
C	0.012990991	−1.418196267	0.000827927
C	−1.181723138	−0.706852625	−0.179834564
C	−1.194748445	0.683849614	−0.182533831
H	−1.534475258	5.540188828	−1.567239456
H	−1.49949902	3.064470842	−1.576249411
H	1.439843761	3.094168259	1.567943365
H	1.429194958	5.570091312	1.554959636
H	−0.064058498	6.807177351	−0.00716082
H	2.116610606	1.254107508	0.304881095
H	2.135622177	−1.212704796	0.337668007
H	−2.113103654	−1.254049557	−0.336607256
H	−2.140916418	1.212957578	−0.310412697
C	0.027380734	−2.902907287	0.003177884
C	−1.021019973	-3.63067647	0.585051437
C	−1.00726813	−5.023553644	0.587960593
C	0.055511715	−5.716365162	0.008535101
C	1.104201335	−5.004686669	−0.573709689
C	1.090086334	−3.611832329	−0.576133823
H	−1.844219368	−3.095969107	1.062571117
H	−1.82778189	−5.57186605	1.053511936
H	0.066448954	−6.807237862	0.010773485
H	1.935533196	−5.538287467	−1.037164786
H	1.90251413	−3.062700122	−1.055722503

10 References

[1] W. Wu, H. Guo, W. Wu, S. Ji, J. Zhao, *J. Org. Chem.* **2011**, *76*, 7056–7064.

[2] P. Seitz, M. Bhosale, L. Rzesny, A. Uhlmann, J. S. Wössner, R. Wessling, B. Esser, *Angew. Chem. Int. Ed.* **2023**, *62*, e202306184.

[3] S. H. Röttger, L. J. Patalag, F. Hasenmaile, L. Milbrandt, B. Butschke, P. G. Jones, D. B. Werz, *Org. Lett.* **2024**, *26*, 3020–3025.

[4] a) L. J. Patalag, L. P. Ho, P. G. Jones, D. B. Werz, *J. Am. Chem. Soc.* **2017**, *139*, 15104–15113; b) A. Patra, L. J. Patalag, P. G. Jones, D. B. Werz, *Angew. Chem. Int. Ed.* **2021**, *60*, 747–752; c) Í. A. O. Bozzi, L. A. Machado, E. B. T. Diogo, F. G. Delolo, L. O. F. Barros, G. A. P. Graça, M. H. Araujo, F. T. Martins, L. F. Pedrosa, L. C. Da Luz, E. S. Moraes, F. S. Rodembusch, J. S. F. Guimarães, A. G. Oliveira, S. H. Röttger, D. B. Werz, C. P. Souza, F. Fantuzzi, J. Han, T. B. Marder, H. Braunschweig, E. N. Da Silva Júnior, *Chem. Eur. J.* **2023**, e202303883.

[5] X. Zhou, C. Yu, Z. Feng, Y. Yu, J. Wang, E. Hao, Y. Wei, X. Mu, L. Jiao, *Org. Lett.* **2015**, *17*, 4632–4635.

[6] C. F. A. Gómez-Durán, I. Esnal, I. Valois-Escamilla, A. Urías-Benavides, J. Bañuelos, I. López Arbeloa, I. García-Moreno, E. Peña-Cabrera, *Chem. Eur. J.* **2016**, *22*, 1048–1061.

[7] M. Chen, K. S. Unikela, R. Ramalakshmi, B. Li, C. Darrigan, A. Chrostowska, S.-Y. Liu, *Angew. Chem. Int. Ed.* **2021**, *60*, 1556–1560.

[8] J. H. Griwatz, M. L. Kessler, H. A. Wegner, *Chem. Eur. J.* **2023**, *29*, e202302173.

[9] a) W. F. K. Wynne-Jones, H. Eyring, *J. Chem. Phys.* **1935**, *3*, 492–502; b) H. Günther, *NMR Spectroscopy*: Basic Principles, Concepts, and Applications in Chemistry, Basic Principles, Concepts, and Applications in Chemistry; Wiley-VCH, Weinheim, Germany, **2013**.

[10] G. M. Sheldrick, *Acta Cryst.* **2015**, *C71*, 3–8.

[11] a) F. Neese, *WIREs Comput. Mol. Sci.* **2022**, *12*, e1606; b) F. Neese, *WIREs Comput. Mol. Sci.* **2012**, *2*, 73–78; c) F. Neese, F. Wennmohs, U. Becker, C. Riplinger, *J. Chem. Phys.* **2020**, *152*, 224108–224125.

[12] a) C. Bannwarth, S. Ehlert, S. Grimme, *J. Chem. Theory Comput.* **2019**, *15*, 1652–1671; b) C. Bannwarth, E. Caldeweyher, S. Ehlert, A. Hansen, P. Pracht, J. Seibert, S. Spicher, S. Grimme, *WIREs Comput. Mol. Sci.* **2021**, *11*, e1493.

[13] P. Pracht, F. Bohle, S. Grimme, *Phys. Chem. Chem. Phys.* **2020**, *22*, 7169–7192.

[14] a) S. Grimme, A. Hansen, S. Ehlert, J.-M. Mewes, *J. Chem. Phys.* **2021**, *154*, 64103–64120; b) M. Bursch, J.-M. Mewes, A. Hansen, S. Grimme, *Angew. Chem. Int. Ed.* **2022**, *61*, e202205735.

[15] a) M. Walker, A. J. A. Harvey, A. Sen, C. E. H. Dessent, *J. Phys. Chem. A* **2013**, *117*, 12590–12600; b) A. Hellweg, D. Rappoport, *Phys. Chem. Chem. Phys.* **2015**, *17*, 1010–1017.

[16] S. Grimme, S. Ehrlich, L. Goerigk, *J. Comput. Chem.* **2011**, *32*, 1456–1465.

[17] S. Grimme, *Chem. Eur. J.* **2012**, *18*, 9955–9964.

[18] T. Lu, F. Chen, *J. Comput. Chem.* **2012**, *33*, 580–592.
